# Exploiting host–guest chemistry to manipulate magnetic interactions in metallosupramolecular M_4_L_6_ tetrahedral cages†

**DOI:** 10.1039/d1sc00647a

**Published:** 2021-03-01

**Authors:** Aaron J. Scott, Julia Vallejo, Arup Sarkar, Lucy Smythe, E. Regincós Martí, Gary S. Nichol, Wim T. Klooster, Simon J. Coles, Mark Murrie, Gopalan Rajaraman, Stergios Piligkos, Paul J. Lusby, Euan K. Brechin

**Affiliations:** EaStCHEM School of Chemistry, The University of Edinburgh David Brewster Road Edinburgh EH93FJ UK E.Brechin@ed.ac.uk Paul.Lusby@ed.ac.uk; Department of Chemistry, Indian Institute of Technology Bombay Powai Mumbai 400076 India rajaraman@chem.iitb.ac.in; WestCHEM, School of Chemistry, University of Glasgow, University Avenue Glasgow G12 8QQ UK; UK National Crystallographic Service, Chemistry, Faculty of Natural and Environmental Sciences, University of Southampton England SO17 1BJ UK; Department of Chemistry, University of Copenhagen, Universitetsparken 5 2100 Copenhagen Denmark piligkos@chem.ku.dk

## Abstract

Reaction of Ni(OTf)_2_ with the bisbidentate quaterpyridine ligand **L** results in the self-assembly of a tetrahedral, paramagnetic cage [Ni^II^_4_**L**_6_]^8+^. By selectively exchanging the bound triflate from [OTf⊂Ni^II^_4_**L**_6_](OTf)_7_ (**1**), we have been able to prepare a series of host–guest complexes that feature an encapsulated paramagnetic tetrahalometallate ion inside this paramagnetic host giving [M^II^X_4_⊂Ni^II^_4_**L**_6_](OTf)_6_, where M^II^X_4_^2−^ = MnCl_4_^2−^ (**2**), CoCl_4_^2−^ (**5**), CoBr_4_^2−^ (**6**), NiCl_4_^2−^ (**7**), and CuBr_4_^2−^ (**8**) or [M^III^X_4_⊂Ni^II^_4_**L**_6_](OTf)_7_, where M^III^X_4_^−^ = FeCl_4_^−^ (**3**) and FeBr_4_^−^ (**4**). Triflate-to-tetrahalometallate exchange occurs in solution and can also be accomplished through single-crystal-to-single-crystal transformations. Host–guest complexes **1–8** all crystallise as homochiral racemates in monoclinic space groups, wherein the four {NiN_6_} vertexes within a single Ni_4_L_6_ unit possess the same *Δ* or *Λ* stereochemistry. Magnetic susceptibility and magnetisation data show that the magnetic exchange between metal ions in the host [Ni^II^_4_] complex, and between the host and the MX_4_^*n*−^ guest, are of comparable magnitude and antiferromagnetic in nature. Theoretically derived values for the magnetic exchange are in close agreement with experiment, revealing that large spin densities on the electronegative X-atoms of particular MX_4_^*n*−^ guest molecules lead to stronger host–guest magnetic exchange interactions.

## Abstract 2

The tetrahedral [Ni^II^_4_**L**_6_]^8+^ cage can reversibly bind paramagnetic MX_4_^1/2−^ guests, inducing magnetic exchange interactions between host and guest.

## Graphical abstract

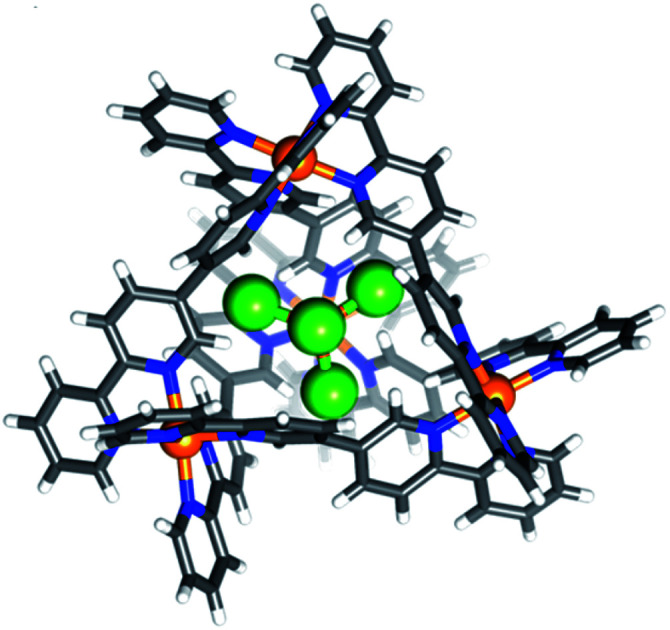

## Introduction

The inherent ability of metallosupramolecular cages to encapsulate different chemical species within their cavity can be exploited for a myriad of applications, including the stabilisation of reactive species,^1^ catalysis,^2,3^ and drug-delivery.^4,5^ In all but a few cases, these cages are constructed from diamagnetic metal ions (most commonly Pd^II^, Pt^II^, Fe^II^, Ru^II^, Ga^III^),^6^ and even when paramagnetic ions (*e.g.* Co^II^) are employed, characterising magnetic properties has not been a key focus.^7,8^ However, the exploitation of (reversible) guest encapsulation to induce magnetic exchange interactions with the host could be used in a variety of potential applications including magnetic sensing and switching,^9^ the construction of single-molecule magnets,^10^ the encapsulation and stabilisation of highly anisotropic single ion magnets with specific geometries,^11,12^ dilution of magnetic molecules in the solid-state,^13^ and the organisation of electron spin based qubits within ordered structural frameworks and/or on surfaces.^14,15^ The latter has proven to be extremely difficult since the magnetic properties of molecules are often changed upon deposition.^15^

Introducing a magnetic guest into the cavity of a magnetic host could result in a number of potential outcomes. (1) Guest encapsulation has no effect, *i.e.* there is no magnetic interaction between host and guest and/or there is no geometrical change in either component. (2) There is no magnetic interaction between host and guest, but binding induces structural changes, altering the geometries of the metal ions in the cage and/or the encapsulated guest, modifying magnetic anisotropy. (3) There is a magnetic interaction between host and guest, which may or may not also change the magnetic exchange between metals ions in the host. (4) There is a combination of points (2) and (3).

In the chemistry of porous coordination polymers, or metal–organic frameworks (MOFs), the ingress of (non-magnetic) guest molecules into the pores of 3D frameworks built from paramagnetic metal ions, such as Co^II^, has shown that even simple solvent molecules can modify the magnetic properties of the metal ions *via* geometry changes induced by intermolecular interactions. The resulting changes in metal anisotropies can lead to significant changes in magnetisation relaxation dynamics.^16^ In spin crossover (SCO) MOFs the high spin – low spin transition temperature is well known to be highly guest-dependent, proffering potential application in molecular recognition.^17,18^ Studies of coordination cages and capsules incorporating a paramagnetic component are limited to the examination of magnetic exchange interactions between metal ions in the cage,^19^ SCO (of the cage and guest),^20–23^ and the interaction of organic-radicals in the cavity (with themselves or the cage)^24–26^ or in the host framework.^27,28^ The ability to understand, and ultimately control, host–guest magnetic exchange interactions and single ion magnetoanisotropies in such molecular species would represent an important step toward making coordination cages with tuneable, and potentially useful, magnetic properties. Herein, we discuss the construction of a tetrahedral cage [Ni^II^_4_**L**_6_]^8+^ (**L** = quaterpyridine) that can (reversibly) bind a range of tetrahedral, paramagnetic MX_4_^1/2−^ guests, inducing magnetic exchange interactions between host and guest.

## Results and discussion

The tetrahedral Ni^II^_4_**L**_6_ cage was synthesised by combining Ni(OTf)_2_ (4 equivalents) with quaterpyridine (**L**, 6 equivalents) in acetonitrile, followed by heating for 24 h (see ESI,† Section 4). The ESI-MS of the isolated complex confirmed the presence of the Ni^II^_4_**L**_6_ cage, with the +2 to +7 *m*/*z* cations being present. The absence of the +8-state strongly suggests that in solution a single triflate anion is bound within the cavity of the cage (Fig. S2–S3†). X-ray crystallography confirms that the structure of **1** is [OTf⊂Ni^II^_4_**L**_6_](OTf)_7_ (see below). To generate the [MX_4_⊂Ni^II^_4_**L**_6_]^*n*+^ host–guest complexes, the solution obtained after 24 h of heating Ni(OTf)_2_ and **L** was treated directly with one equivalent of tetraethylammonium tetrahalometallate salt, giving [M^II^X_4_⊂Ni_4_**L**_6_](OTf)_6_ where M^II^X_4_^2−^ = MnCl_4_^2−^ (**2**), CoCl_4_^2−^ (**5**), CoBr_4_^2−^ (**6**), NiCl_4_^2−^ (**7**) and CuBr_4_^2−^ (**8**); [M^III^X_4_⊂**1**](OTf)_7_ where M^III^X_4_^−^ = FeCl_4_^−^ (**3**) and FeBr_4_^−^ (**4**). The displacement of the encapsulated triflate is confirmed both by X-ray crystallography (see below) and also by ESI-MS. In this case, ESI-MS (ESI,† Section 4) shows that the highest charged species correspond to [M^II^X_4_⊂Ni_4_**L**_6_]^6+^ when M is a divalent metal ion, and [M^III^X_4_⊂Ni_4_**L**_6_]^7+^ when M is trivalent. The selectivity of the anion exchange process, wherein a single equivalent of tetrahalometallate displaces the encapsulated triflate rather than any of the external counteranions, can partly be explained by the higher charge of MX_4_^2−^ (*e.g.* where M = Mn^II^, Co^II^, Ni^II^ and Co^II^). However, as singly charged FeX_4_^−^ (X = Cl, Br) also displaces the bound triflate, this selectivity is not purely a Coulombic effect, and is likely caused by the shape complementarity of the tetrahedral tetrahalometallate guest for the cage's pseudo-tetrahedral cavity.

Host–guest complexes **2–8** can also be reversibly formed through single crystal to single crystal transformations. For example, when orange crystals of **1** are soaked in an EtOH solution of (Et_4_N)_2_NiCl_4_ for 2 hours, green crystals of **7** are formed. The process is reversed by soaking crystals of **7** in an EtOH solution of ^*n*^Bu_4_NOTf (Fig. S1†).

### Crystal structure descriptions

Single crystals of [OTf⊂Ni^II^_4_**L**_6_](OTf)_7_ (**1**) and [MX_4_⊂Ni^II^_4_**L**_6_](OTf)_6/7_ (**2–8**) were obtained from vapour diffusion of THF and/or Et_2_O into the MeCN mother liquor. Synchrotron radiation was required to obtain single crystal data for complexes **2** (MX_4_ = MnCl_4_^2−^), **7** (MX_4_ = NiCl_4_^2−^) and **8** (MX_4_ = CuCl_4_^2−^).^29^ All eight complexes crystallise in monoclinic cells, with **1–4**, **7** and **8** being in the space group *C*2/*c*, and **5** and **6** in the *P*2_1_/*n* and *P*2_1_/*c* space groups, respectively (Tables S1 and S2†).

**Fig. 1:**
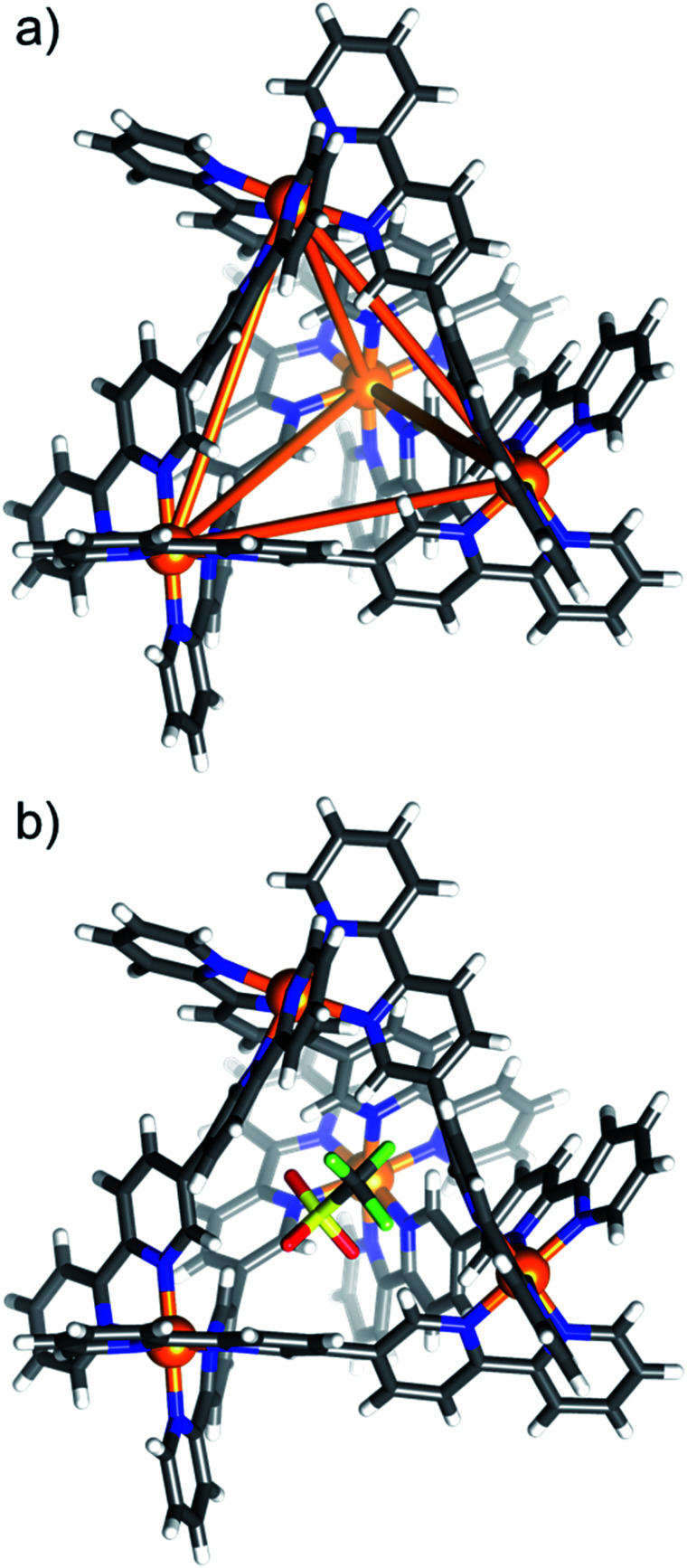
(a) Framework of the empty [Ni^II^_4_**L**_6_]^8+^ tetrahedron emphasising the connectivity of the assembly. (b) Structure of the [OTf⊂Ni^II^_4_**L**_6_]^7+^ host–guest tetrahedron of **1**. Non-encapsulated triflate anions and solvent of crystallisation are removed for clarity, highlighting the connectivity between the bisbidentate quaterpyridine ligands **L** and the Ni^II^ ions. Colour code: Ni = orange, N = blue, C = grey, H = white.

The structures of compounds **1–8** are very similar, and so for the sake of brevity, a generic description is provided. Pertinent bond lengths and angles given in Table S3.† The cationic cage describes a [Ni^II^_4_**L**_6_]^8+^ tetrahedron, which, akin to other M_4_L_6_ assemblies, has the Ni^II^ ions occupying the four vertices linked by bisbidentate **L** ligands lying on each of the six edges (Fig. 1a). The approximate dimensions of the tetrahedron in **1–8** are Ni^II^⋯Ni^II^ = 9.2–9.6 Å, with the internal cage volumes ranging from 62–81 Å^3^. The volumes, pore diameters and average window diameters for each compound are given in Table S4.†^30^ The Ni^II^ ions are six-coordinate and in distorted NiN_6_ octahedral geometries, with Ni–N distances between 2.03(3)–2.17(3) Å and *cis*/*trans* angles in the range 76.4(12)–102.28(13)°/170.3(11)–176.6(6)°, respectively. Each tetrahedron has T-symmetry, possessing four metallic vertices with the same stereochemistry (*Δ* or *Λ*). All compounds crystallise as racemic mixtures of the homochiral cage (*i.e.* an equal mixture of *ΔΔΔΔ* and *ΛΛΛΛ* stereoisomers).

Consistent with the ESI-MS observations, **1** has a positionally disordered triflate anion occupying the cavity (Fig. 1b). There are interactions between the O and F atoms of the anion and the inward facing *ortho*-pyridyl H atoms of **L** (O/F⋯H−Ar ≈ 2.52 Å). The remaining seven triflate anions surround the exterior of the tetrahedron, maintaining charge balance. These, and the solvent of crystallisation, are involved in a number of intermolecular interactions that connect neighbouring cages.

**Fig. 2:**
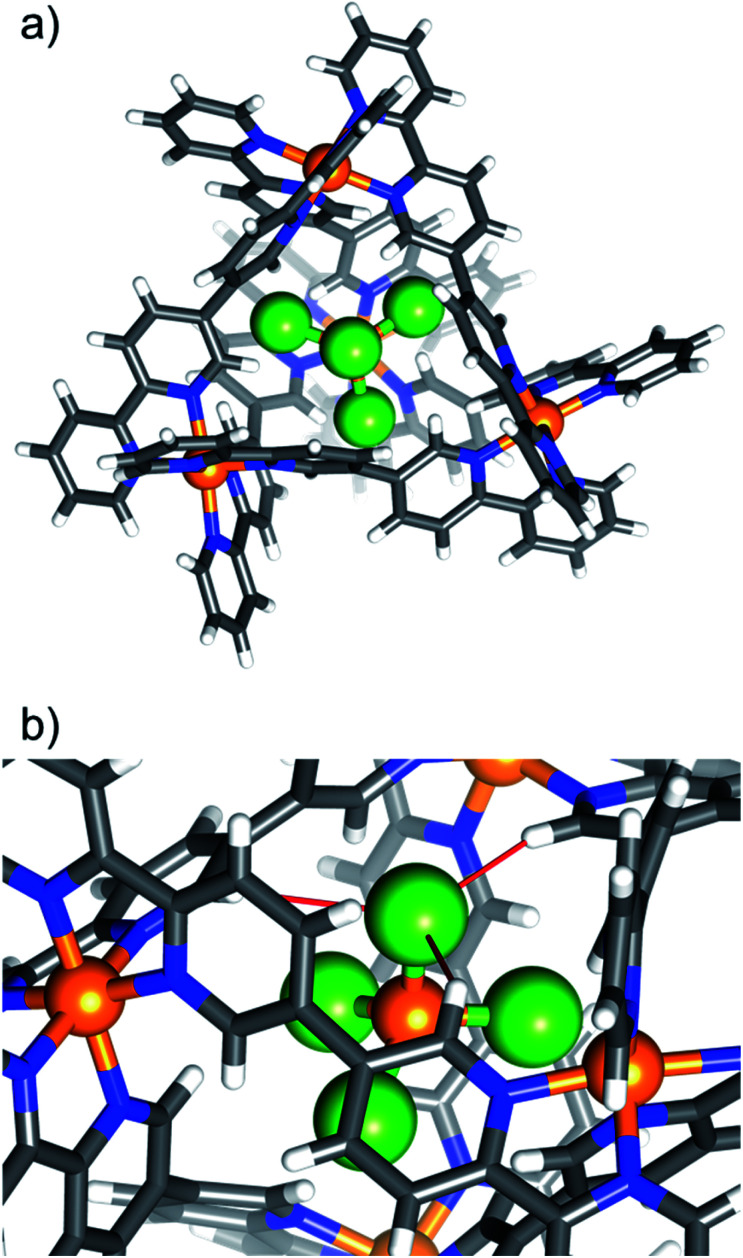
(a) Portal-view of the [NiCl_4_⊂Ni^II^_4_**L**_6_]^6+^ host–guest tetrahedron of **7**, illustrating the position of the encapsulated [NiCl_4_]^2−^. The tetrahalometallate guest sits with the halide ions pointing towards the cage portals. (b) A close-up of the guest in the host cage highlighting the closest intermolecular interactions (red bonds). Colour code as Fig. 1. Cl = green.

Complex **7**, [NiCl_4_⊂Ni^II^_4_**L**_6_](OTf)_6_, is shown in Fig. 2 as a representative tetrahalometallate-cage structure (for depictions of **2–6** and **8**, along with pertinent bond lengths and angles, see ESI†). With the exception of **4**, which shows a 1 : 1 partial occupancy of FeBr_4_^−^ and OTf^−^, the tetrahalometallate guests are positionally ordered with full occupancy, showing regular tetrahedral geometry. In each case the guest anion is positioned such that the MX_4_^*n*−^ tetrahedron is inverted with respect to the cage's [Ni_4_**L**_6_]^8+^ tetrahedron, *i.e.* the halide atoms point towards of the portals of the tetrahedron. The host–guest interactions are similar to **1**, with the closest contacts between the tetrahalometallate halide atoms and the *ortho*-pyridyl positions of the cage's ligand. For example, in **7**, the distances between the host and guest are: Cl⋯H−Ar ≈ 2.86 Å (Fig. 2b). As with **1**, the external triflate anions and solvent of crystallisation connect neighbouring cages through a network of interactions with the host framework. In the extended structure this results in alternating layers of cages/anions and solvent molecules of crystallisation (Fig. S18–S34, Table S5†).

### SQUID magnetometry

**Fig. 3:**
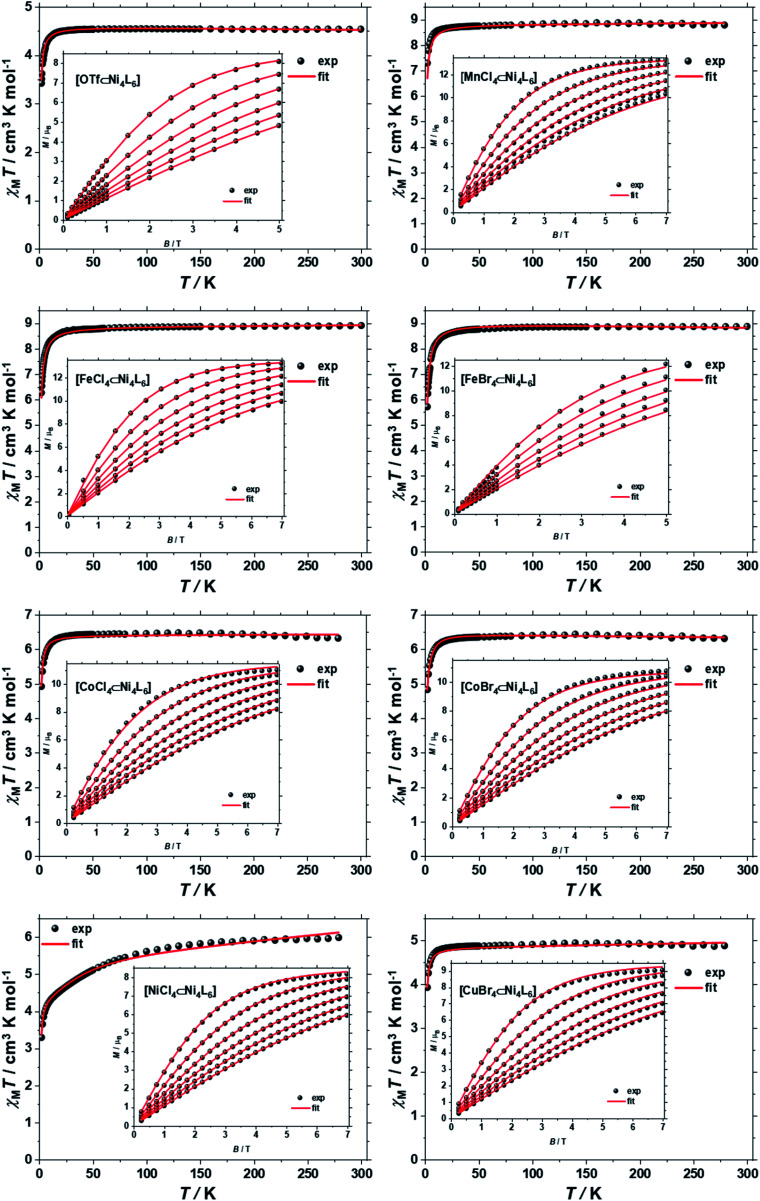
*χ*_M_ *T versus T* plots for [OTf⊂Ni^II^_4_**L**_6_](OTf)_7_ (**1**) and [M^II^X_4_⊂Ni^II^_4_**L**_6_](OTf)_6_, where M^II^X_4_^2−^ = MnCl_4_^2−^ (**2**), CoCl_4_^2−^ (**5**), CoBr_4_^2−^ (**6**), NiCl_4_^2−^ (**7**), CuBr_4_^2−^ (**8**) or [M^III^X_4_⊂Ni^II^_4_**L**_6_](OTf)_7_, where M^III^X_4_^−^ = FeCl_4_^−^ (**3**), FeBr_4_^−^ (**4**) in the range *T* = 2–300 K and *B* = 0.1 T. The insets show the field dependence of the magnetisation measured in the *T* = 2–7 K and *B* = 0–7.0 T temperature and field ranges. The black spheres are the experimental data and the red lines the fit of the experimental data using spin-Hamiltonian (1), as explained in the text.

The direct-current (d.c.) molar magnetic susceptibility, *χ*_M_, of polycrystalline samples of **1–8**, were measured in an applied magnetic field, *B*, of 0.1 T, over the 2–300 K temperature, *T*, range. The results are plotted in Fig. 3 in the form of the *χ*_M_*T* product, where *χ*_M_ = *M*/*B* with *M* the magnetisation. At room temperature the *χ*_M_*T* products of **1–8** are 4.52, 8.86, 8.87, 8.82, 6.42, 6.35, 6.12 and 4.92 cm^3^ K mol^−1^, respectively. These values are close to the Curie constants expected for uncorrelated paramagnetic centres (4.54, 8.92, 8.92, 6.42, 6.42, 5.67 and 4.92) with *g* = 2 for all metal ions, except for Ni^II^ where *g*_Ni_ = 2.13 (*vide infra*). With the exception of **7**, on lowering the temperature, the *χ*_M_*T* products of **1–8** are essentially constant down to the temperature range 50–20 K, whereupon a further decrease of temperature results in the gradual drop of the *χ*_M_*T* products of all complexes, to reach their respective minimum values at 2 K. This behaviour is indicative of weak antiferromagnetic interactions operating in **1–8**. The faster drop of the *χ*_M_*T* product of **7** from ∼100 K indicates that the tetrahedral Ni^II^ guest displays significant magnetic anisotropy, of the same order of magnitude as the temperature.

To better define the low temperature magnetic properties of **1–8**, we performed variable-temperature-variable-field (VTVB) dc magnetisation measurements on polycrystalline samples in the temperature range 2–7 K and in applied magnetic fields up to 7 T. The results of these VTVB measurements are given in the insets of Fig. 3 as the field dependent magnetisation, and as the magnetisation dependence against the reduced quantity *μ*_B_*B*/*kT* with μ_B_ and *k* the Bohr magneton and Boltzman constant, respectively, (Fig. S35–S42†) that expresses the ratio between Zeeman and thermal energies. Inspection of these reduced magnetisation traces reveals that the ground states of **1–8** are weakly anisotropic, as evidenced by the limited nesting of the curves. Thus, the magnetic anisotropy of the constitutive single ions is either very small, as expected for Cu^II^, Fe^III^ and Mn^II^, or very large (Ni^II^) with respect to the experimental conditions (*B*, *T*). For the quantitative interpretation of the magnetic properties of **1–8**, we used spin-Hamiltonian (1):

1 *Ĥ* = *Ĥ*_host_ + *Ĥ*_guest_

with2
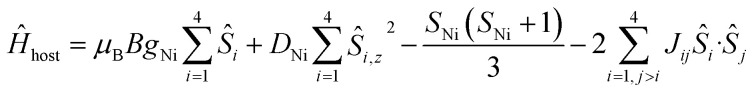
and3
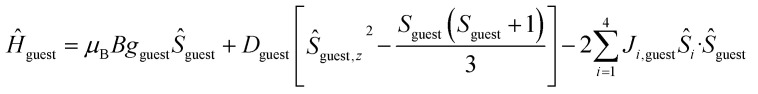
where *Ĥ*_host_ is the spin-Hamiltonian relative to **1**, *Ĥ*_guest_ is the Hamiltonian relative to the guests in **2–8** and their interaction with the host **1**, *i*, *j* are indices that run over the constitutive centres, *g*_Ni_ the *g*-value of Ni^II^, *Ŝ*_*i*_ the spin operator of the *i*^th^ paramagnetic centre, *D*_Ni_ the single-ion axial anisotropy parameter of Ni^II^, *S*_Ni_ = 1 the total spin of Ni^II^, *J*_*ij*_ the pairwise isotropic magnetic exchange interaction parameter between centres *i* and *j*, with the equivalent quantities for the guests.

The *χ*_M_*T* product and the VTVB data for **1–8** were simultaneously fitted to spin-Hamiltonian (1) by full matrix numerical diagonalisation of its matrix representation and by use of the Simplex algorithm.^31^ For **1**, fitting of the *χ*_M_*T* product and the VTVB data resulted in the best-fit parameters: *g*_Ni_ = 2.13, |*D*_Ni_| = 1.575 cm^−1^ and *J*_Ni–Ni_ = −0.078 cm^−1^ (Fig. 3). The relatively small uniaxial anisotropy parameter, *D*_Ni_, of the Ni^II^ centres of the host is in agreement with their approximate octahedral symmetry. These parameters were subsequently fixed for the quantitative interpretation of the magnetic properties of **2–8**. For simplicity, the *g*-values of all guests were fixed to 2, except for **7** where we set the *g*-value of the guest Ni^II^ ion equal to *g*_Ni_ = 2.13, as determined for **1**. Thus for **2** the model contained only one free parameter, namely *J*_Ni–Mn_. Simultaneous fitting of the *χ*_M_*T* and VTVB data of **2** resulted in the best-fit parameters: *J*_Ni–Mn_ = −0.041 cm^−1^. Analogously, the best fit parameters for **3** were: *J*_Ni–Fe_ = −0.068 cm^−1^ (*D*_Fe_ was neglected for Fe^III^); for **4**: *J*_Ni–Fe_ = −0.084 cm^−1^ (*D*_Fe_ was neglected for Fe^III^); for **5**: |*D*_Co_| = 2.37 cm^−1^ and *J*_Ni–Co_ = −0.005 cm^−1^; for **6**: |*D*_Co_| = 6.30 cm^−1^ and *J*_Ni–Co_ = −0.001 cm^−1^; for **7**: |*D*′_Ni_| = 85.5 cm^−1^ and *J*′_Ni_–_Ni_ = −0.476 cm^−1^; and for **8**: *J*_Ni–Cu_ = −0.062 cm^−1^ (*D*_Cu_ was neglected for Cu^II^). The values are tabulated in Table 1 for convenience. Note that the large *D*′_Ni_ value for the guest in **7** is of the magnitude expected for a tetrahedral Ni^II^ ion, and in agreement with the faster drop of the *χ*_M_*T* product with decreasing temperature, absent for all other compounds. For these other compounds, the guest magnetic anisotropy is approximately two orders of magnitude smaller, or entirely negligible.

**Table 1:** Exchange interactions (*J*) and axial zero-field splitting (*D*) parameters for **1–8** derived from a simultaneous fit of the susceptibility and magnetisation data employing spin-Hamiltonian (1)

	*J* (cm^−1^)	|*D*| (cm^−1^)
1	*J*_Ni–Ni_ = −0.078 (no MX_4_ guest – fixed for **2–8**)	|*D*_Ni_| = 1.575 (no MX_4_ guest – fixed for **2–8**)
2	*J*_Ni–Mn_ = −0.041	Neglected for Mn^II^
3	*J*_Ni–Fe_ = −0.068	Neglected for Fe^III^
4	*J*_Ni–Fe_ = −0.084	Neglected for Fe^III^
5	*J*_Ni–Co_ = −0.005	|*D*_Co_| = 2.37
6	*J*_Ni–Co_ = −0.001	|*D*_Co_| = 6.30
7	*J*′_Ni_–_Ni_ = −0.476	|*D*′_Ni_| = 85.5
8	*J*_Ni–Cu_ = −0.062	Neglected for Cu^II^

### Theoretical studies

The magnetic properties of all eight complexes have been investigated using both DFT and *ab initio* methods. DFT calculations have been employed to estimate the isotropic exchange coupling constants (*J*) using the Hamiltonian 
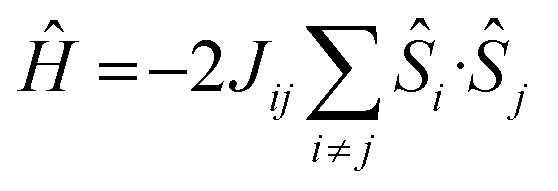
. On-site spin–orbit coupling/zero-field splitting (zfs) calculations require accurate estimation of excited state energies, and a single-determinant description of the wavefunction is not sufficient for systems having orbital degeneracies or those possessing low-lying excited states. For this reason, *ab initio* CASSCF/NEVPT2 calculations have been performed (see the Computational Details in the ESI for more information).

### Electronic structure of the empty Ni_4_L_6_ cage

**Fig. 4:**
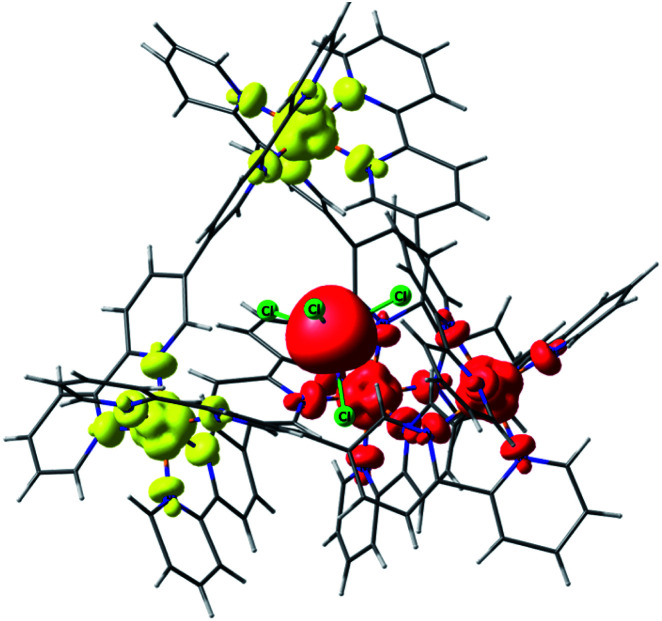
Lowest energy broken symmetry spin density plot for complex **2**. Iso-surface value, 0.005 e^−^/Bohr.^3^ The red and yellow colours represent “spin-up” and “spin-down”, respectively.

Calculations have been performed on the full X-ray structure without any geometry relaxation. The *J*_Ni–Ni_ interaction is estimated to be −0.062 cm^−1^, very close to the experimental value of −0.078 cm^−1^ (Table 2). The magnitude of the antiferromagnetic exchange suggests that the SOMOs of neighbouring Ni^II^ ions are weakly interacting. The distance between metal ions is not particularly long (∼9.5 Å), but significant twisting between the bipyridine units (Fig. 1 and 4; dihedral angle = 45–60°) hinders the spin polarisation mechanism. Spin density values on the Ni^II^ ions in **1** are found to be ∼1.64, which is as expected for octahedral Ni^II^ centres possessing strong spin delocalisation (Fig. S43†). The spin ground state is found to be an *S* = 0 state with two “spin-up” and two “spin-down” Ni^II^ ions. The axial zfs of the octahedral Ni^II^ ions in the cage is found to be *D* = −2.25 cm^−1^, in agreement with the experimental data. Such a value would be expected given the high symmetry and close-to-perfect octahedral geometry around the Ni^II^ centres.^32,33^ For the CASSCF/NEVPT2 calculations, the other Ni^II^ centres were substituted by diamagnetic Zn^II^ ions.

**Table 2:** Comparison of experimental and computational spin-Hamiltonian parameters for complexes **1–8**

	Exp *J* (cm^−1^)	Cal *J* (cm^−1^)	Exp |*D*| (cm^−1^)	Cal *D* (cm^−1^)	Exp *g*-factors/*g*_iso_	Calc. *g*-factors/*g*_iso_/*g*_*x*_, *g*_*y*_, *g*_*z*_
**1**	*J*_Ni–Ni_ = −0.078 (no MX_4_ guest – fixed for **2–8**)	*J*_Ni–Ni_ = −0.062	1.575 (fixed for **2–8**)	−2.25 (*E*/*D* = 0.15) for Ni^II^	2.13	*g*_eff_ = 2.165, 2.171, 2.184
**2**	*J*_Ni–Mn_ = −0.041	*J*_Ni–Mn_ = −0.011	Neglected for Mn^II^	−0.002 (*E*/*D* = 0.08) for Mn^II^		*g*_eff_ = 2.00 for Mn
	*J*_Ni–Ni_ = −0.078	*J*_Ni–Ni_ = −0.090				
**3**	*J*_Ni–Fe_ = −0.068	*J*_Ni–Fe_ = −0.073	Neglected for Fe^III^	0.01 (*E*/*D* = 0.26) for Fe^III^		*g*_eff_ = 2.00 for Fe
	*J*_Ni–Ni_ = −0.078	*J*_Ni–Ni_ = −0.074				
**4**	*J*_Ni–Fe_ = −0.084	*J*_Ni–Fe_ = −0.085	Neglected for Fe^III^	−0.07 (*E*/*D* = 0.16) for Fe^III^		*g*_eff_ = 2.00 for Fe
	*J*_Ni–Ni_ = −0.078	*J*_Ni–Ni_ = −0.073				
**5**	*J*_Ni–Co_ = −0.005	*J*_Ni–Co_ = +0.012	(Co) 2.37	−6.64 (*E*/*D* = 0.30) for Co^II^		Co: *g*_eff_ = 1.557, 2.086, 6.619
	*J*_Ni–Ni_ = −0.078	*J*_Ni–Ni_ = −0.079				
**6**	*J*_Ni–Co_ = −0.001	*J*_Ni–Co_ = +0.025	(Co) 6.30	−3.96 (*E*/*D* = 0.17) for Co^II^		Co: *g*_eff_ = 1.038, 1.234, 6.993
	*J*_Ni–Ni_ = −0.078	*J*_Ni–Ni_ = −0.076				
**7**	*J′*_Ni_–_Ni_ = −0.476	—	(Ni) 85.5	213.5 (*E*/*D* = 0.26) for Ni^II^_tet_		Ni_tet_: *g*_eff_ = 3.493, 2.864, 1.689
	*J*_Ni–Ni_ = −0.078					
**8**	*J*_Ni–Cu_ = −0.062	*J*_Ni–Cu_ = −0.066	Neglected for Cu^II^	—	(Cu) 2.00	Cu: *g*_iso_ = 2.43
	*J*_Ni–Ni_ = −0.078	*J*_Ni–Ni_ = −0.064				

### Electronic structure of the MX_4_⊂Ni_4_L_6_ cages (**2–8**)

For complex **2**, incorporation of the MnCl_4_^2−^ anion inside the [Ni_4_L_6_]^8+^ cage introduces an exchange interaction between host and guest (*J*_Mn–Ni_) in addition to the *J*_Ni–Ni_ exchange. The spin state energies of the host–guest molecules have been computed considering a pentametallic MnNi_4_ unit. The *J*_Ni–Ni_ and the *J*_Mn–Ni_ exchange interactions are estimated to be −0.09 cm^−1^ and −0.01 cm^−1^, respectively. Here the spin ground state is *S* = 5/2 (Fig. 4, S44 and Table S6† (BS3)). The weaker *J*_Mn–Ni_ exchange originates from the dipolar Cl⋯H−Ar interaction which mediates the coupling. The zfs of the Mn^II^ ion is estimated to be very small, *D* = −0.002 cm^−1^, in accordance with the isotropic nature of a tetrahedral d^5^ centre.^34^

Similar analyses were performed on the remaining host–guest complexes. For complex **3**, the *J*_Fe–Ni_ and *J*_Ni–Ni_ interactions are found to be −0.073 cm^−1^ and −0.074 cm^−1^, respectively (Table 2). Note that *J*_Fe–Ni_ is stronger than *J*_Mn–Ni_: a closer examination of the spin densities computed on the Cl atoms of **2** and **3** reveals stronger delocalisation of the spin density in **3** compared to that in **2** facilitating stronger exchange interactions. This is correlated to the shorter Fe–Cl distance (2.20 Å) in **3** compared to the Mn–Cl distance (2.38 Å) in **2**. The CASSCF/NEVPT2 computed *D* values for the guest ions in complexes **2–4** are small. Indeed, they are smaller than the energy separation between the spin state energies arising from the exchange interaction.

A similar situation is observed for complex **4** with [FeBr_4_]^−^ as the guest. In this case the host–guest antiferromagnetic exchange is larger (−0.085 cm^−1^) than for **2** and **3**. The spin density on the Fe^III^ ion is significantly reduced due to strong spin delocalisation on to the electronegative Cl^−^ and Br^−^ ions in **3** and **4** (Table S6†). Interestingly, in complexes **5** and **6**, where [CoCl_4_]^2−^ and [CoBr_4_]^2−^ are the guest molecules, *J*_Co–Ni_ was found to be weakly ferromagnetic from DFT calculations (+0.012 and +0.025 cm^−1^, respectively; Table 2). The sign of *J*_Co–Ni_ is contrary to experiment, albeit both the magnitude of the exchange and the absolute difference in the exchange is extremely small. Thus, both experiment and theory point to the presence of extremely weak exchange in this instance, and we note that these particular *J* values are at the limit of what DFT can accurately reproduce. More importantly, the *D*_Co_ values determined from *ab initio* methods are −6.64 cm^−1^ (**5**) and −3.96 cm^−1^ (**6**), three orders of magnitude higher than the energy separation between the exchange-coupled spin states, *i.e.*, |*D*| > *J*. The *M*_S_ level separations, *i.e.*, the gap between the ±3/2 and ±1/2 microstates for Co^II^ are much larger (∼2|*D*|) and close to 13 cm^−1^ and 8 cm^−1^, respectively. Deviation from ideal *T*_d_ symmetry is well-known to result in a significant *D* value for tetrahedral Co^II^ ions.^35–38^

**Fig. 5:**
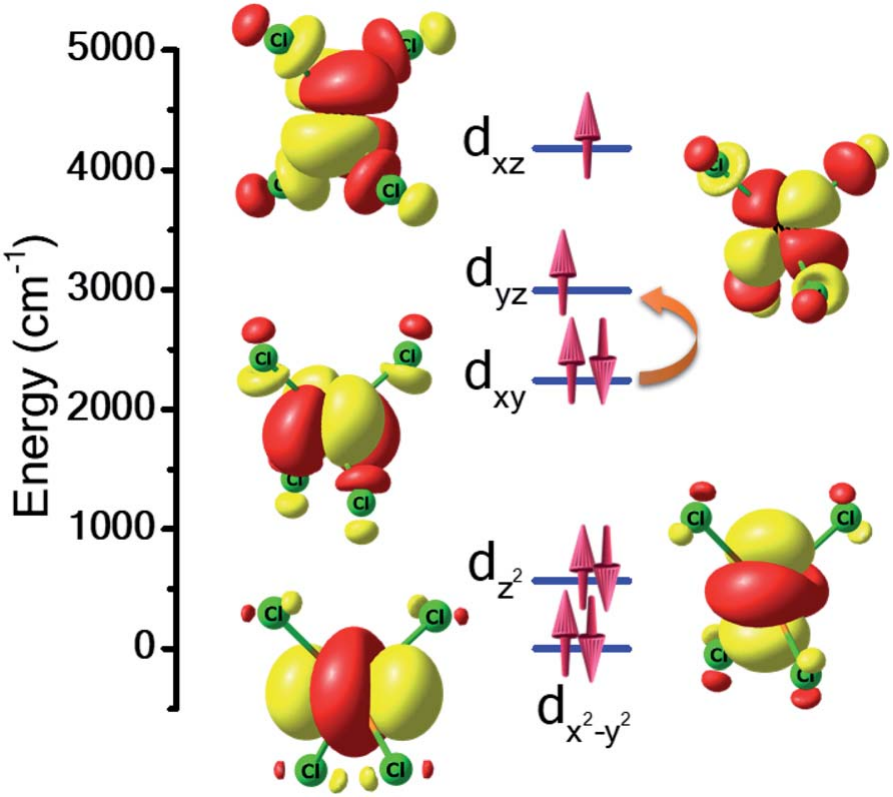
NEVPT2-LFT computed d-orbital splitting diagram for the [NiCl_4_]^2−^ guest molecule in **7**. The orange curly arrow represents the most dominant electronic excited state contribution to the zfs/very large *D* value.

For complex **7**, the orbital degeneracy of the tetrahedral Ni^II^ ion precluded convergence of the DFT calculations and thus no host–guest *J*_Ni–Ni_ exchange coupling could be estimated. NEVPT2 calculations yield a *D* value of +214 cm^−1^ for [NiCl_4_]^2−^, a value much larger than that estimated from experimental susceptibility and magnetisation data.^39^ The origin of this very large anisotropy can be explained from the NEVPT2-LFT orbital splitting pattern of the pseudo-*T*_d_ [NiCl_4_]^2−^ complex shown in Fig. 5. Here the first four excited states contribute strongly to the large positive *D* value, and arise primarily from the d_*xy*_ → d_*yz*_/d_*xz*_ and d_*x*^2^−*y*^2^_ → d_*yz*_/d_*xz*_ electronic excitations (Table S7†). CASSCF/NEVPT2 calculations carried out on the DFT optimised [NiCl_4_]^2−^ geometry yield a similar value, *D* = +210 cm^−1^. We have also computed the deformation energy of [NiCl_4_]^2−^ inside the cage. This is calculated as the difference in energy between the DFT optimised structure and the single-point energy calculated on the guest [NiCl_4_]^2−^. This is estimated to be 18.6 kJ mol^−1^, which indicates a small structural distortion upon encapsulation.

In complex **8**, the exchange interactions between Ni–Ni and Ni–Cu ions are rather similar, *J*_Ni–Ni_ = −0.064 cm^−1^ and *J*_Ni–Cu_ = −0.066 cm^−1^. The spin ground state is *S* = 1/2 (BS3, Fig. S45†). Due to strong spin delocalisation from the Cu^II^ ion onto its four Br^−^ ions the spin density value on the metal ion reduces to just 0.39.

The theoretically determined spin-Hamiltonian parameters have been used to simulate the experimental susceptibility and magnetisation data (Fig. S46–S47†). During simulation, we adopted three different spin-Hamiltonians to describe the magnetic properties for the eight complexes.

For complexes **1–4** and **8** both the metal ions in the host cage and in the guest anions are found to be completely isotropic – the zfs/*D* values of the Mn^II^, Fe^III^ and Ni^II^(cage) centres are very small and comparable to the *J* values (*i.e. D* ≈ *J*). For these five species, spin-Hamiltonian (4) was employed for simulation. All give very good agreement with the experimental data (Fig. S46†).

For complex **8**, the *g*-factors for the Cu^II^ ion from the NEVPT2 level of theory, *g*_*x*_ = 1.633, *g*_*y*_ = 2.126 and *g*_*z*_ = 4.078 are overestimated. This is a well-known problem in the literature for Cu^II^. Higher electron correlation, higher reference space and/or ligand orbital inclusion, should be taken into consideration.^40^ We have therefore performed multi-reference CI (MRCI) calculations in combination with the CASSCF wavefunction to obtain the final *g*-factors, *g*_*x*_ = 1.945, *g*_*y*_ = 2.470 and *g*_*z*_ = 2.879 (*g*_iso_ = 2.43), which remain anisotropic due to mixing with the bromide orbitals. Simulation of the susceptibility and magnetisation data shows excellent agreement with the experimental data (Fig. S47†).4
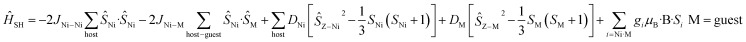


For complexes **5** and **6**, the *D* parameters of Co^II^ are small but still much larger than the spin state energies/*J* values (*i.e.* |*D*| > *J*). In these two cases, we have used spin-Hamiltonian (5) to simulate the experimental susceptibility and magnetisation data. Instead of using *D* values for the Co^II^ ion, ground state effective *g*-factors for individual Kramers pairs are used as *S̃*= 1/2 pseudo-spins (Ising Hamiltonian; Table 2).^41^ This produces a very nice simulation of the experimental data, given the simplicity of the model (Fig. S46–S47†). Note that the simulations are unaffected by the sign of the *J*_Ni–Co_ exchange.5
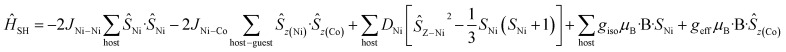
6
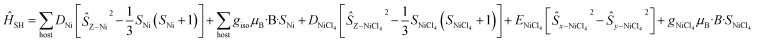


For complex **7**, which contains the highly anisotropic [NiCl_4_]^2−^ guest anion, we have used spin-Hamiltonian (6) in which all exchange interactions are neglected, since *D*_Ni_ ⋙ *J* [the inclusion of any reasonable *J*_host–guest_ value does not affect the simulation]. Note that the *g*-factors obtained from the NEVPT2 method for [NiCl_4_]^2−^ are overestimated, as expected for the highly anisotropic Ni^II^ ion.^42^ Simulation of susceptibility and magnetisation data is given in Fig. S47† and shows good agreement with the experimental data, albeit of a slightly larger magnitude.

## Conclusions

The tetrahedral cage [OTf⊂Ni^II^_4_**L**_6_](OTf)_7_ (**1**) can be synthesised from the one pot reaction of Ni(OTf)_2_ and quaterpyridine (**L**) in acetonitrile. The analogous host–guest complexes, [M^II^X_4_⊂Ni_4_**L**_6_](OTf)_6_ = MnCl_4_^2−^ (**2**), CoCl_4_^2−^ (**5**), CoBr_4_^2−^ (**6**), NiCl_4_^2−^ (**7**) and CuBr_4_^2−^ (**8**), [M^III^X_4_⊂Ni_4_**L**_6_](OTf)_7_ = FeCl_4_^−^ (**3**) and FeBr_4_^−^ (**4**) are formed from **1** by the selective exchange of the encapsulated triflate anion. The complexes can also be formed and interconverted through single-crystal-to-single-crystal transformations.

Magnetic susceptibility and magnetisation data show that the magnetic exchange interactions between metal ions in the host complex, and between host and guest, are of comparable magnitude and antiferromagnetic in nature. Theoretically derived values for the exchange are in close agreement with experiment and reveal that large spin densities on the electronegative X-atoms of certain MX_4_^*n*−^ guest molecules leads to stronger host–guest magnetic exchange interactions. For the tetrahedral Co^II^ guests, the anisotropy is small but still much larger than the magnitude of exchange coupling between host–host and host–guest. The orbital degeneracy of the tetrahedral Ni^II^ ion and the very large zfs that results makes accurate estimation of *J*_Ni–Ni_ and *D*_Ni(tet)_ in (**7**) rather difficult, as reflected in the large differences in the results obtained between experiment and theory.

What is clear, however, is that the encapsulation of paramagnetic guests inside dia/paramagnetic cages can be very useful in an array of potential applications. These include sensing and switching, the encapsulation and stabilisation of highly anisotropic (and/or air- and moisture-sensitive) magnetic molecules and the organisation (and/or dilution) of magnetic molecules within ordered, solution-stable structural matrices. To date, surface deposition of magnetic molecules has proved problematic, since in the vast majority of cases structural/magnetic integrity is compromised hindering application. Encapsulation of metal complexes such as spin crossover species, single-ion magnets (SIMs) or electron spin based qubits within a dia/paramagnetic cage whose exohedral organic skeleton is easily derivatised may prove to be an interesting option. The cage acting both as a surface anchor and a protective coating for the magnetic molecule.^43,44^

Although only relatively small changes to the geometries of the MX_4_^*n*−^ guests were observed here, this work also suggests that the deliberate distortion/construction of magnetic molecules through encapsulation within the confines of a sterically restricted cavity of a coordination cage may offer an alternative route to producing highly unusual/anisotropic SIMs with specific geometries, tailored ligand fields, and targeted symmetries. In turn, such species may display a breadth of fascinating structures and magnetisation relaxation dynamics that may not exist outwith the cage.

## Author contributions

AJS and JV performed the experimental work. AS and GP performed the theoretical studes. AJS, JV, LS, ERM, MM and SP measured the magnetic data. SP and AJS fitted the magentic data. WTK and SJC collected and solved the single crystal X-ray diffraction data. PJL and EKB conceived the idea. AJS, GR, SP, PJL and EKB wrote, reviewed and edited the manuscript.

## Conflicts of interest

There are no conflicts to declare.

## Supplementary Material

SC-012-D1SC00647A-s001

SC-012-D1SC00647A-s002
