# Supplementary material for: Exploiting host–guest chemistry to manipulate magnetic interactions in metallosupramolecular M4L6 tetrahedral cages
Source: Chem Sci. 2021 Mar 1;12(14):5134–42. doi: 10.1039/d1sc00647a (PMC8179613; doi:10.1039/d1sc00647a)
Supplement: SC-012-D1SC00647A-s001 [file SC-012-D1SC00647A-s001.pdf]

Electronic Supplementary Material (ESI) for Chemical Science.
This journal is © The Royal Society of Chemistry 2021

Supporting Information

Exploiting Host-Guest Chemistry to Manipulate Magnetic Interactions in Metallosupramolecular M_4L_6 Tetrahedral Cages

Aaron J. Scott,[a] Julia Vallejo,[a] Arup Sarkar,[b] Lucy Smythe,[c] E. Regincós Martí,[c] Gary S. Nichol,[a] Wim T. Klooster,[d] Simon J. Coles,[d] Mark Murrie,[c] Gopalan Rajaraman,*[b] Stergios Piligkos,*[e] Paul J. Lusby*[a] and Euan K. Brechin*[a]

[a.] EaStCHEM School of Chemistry, The University of Edinburgh, David Brewster Road, Edinburgh, EH93FJ, UK. E.Brechin@ed.ac.uk; Paul.Lusby@ed.ac.uk.
[b.] Department of Chemistry, Indian Institute of Technology Bombay, Powai, Mumbai 400076, India. E-mail: rajaraman@chem.iitb.ac.in
[c.] WestCHEM, School of Chemistry, University of Glasgow, University Avenue, Glasgow, G12 8QQ, UK.
[d.] UK National Crystallographic Service, Chemistry, Faculty of Natural and Environmental Sciences, University of Southampton, England SO17 1BJ, UK
[e.] Department of Chemistry, University of Copenhagen, Universitetsparken 5, 2100 Copenhagen, Denmark. E-mail: piligkos@chem.ku.dk

Contents

1 Materials and Methods 3

2 Synthesis 5

3 Single-Crystal-to-Single-Crystal Transformation of 1↔5 9

4 Mass Spectrometry 10

5 Crystallography 18

6 Magnetometry 41

7 Computational Details 45

8 References 54

1 Materials and Methods

Unless stated otherwise, all reagents and solvents were purchased from Alfa Aesar, VWR, Fluorochem or Sigma Aldrich and used without further purification. Where the use of anhydrous solvent is stated, drying was carried out using a solvent purification system manufactured by Glass Contour. Column chromatography was carried out using Geduran Si60 (40–63 µm) as the stationary phase and TLC was performed on precoated Kieselgel 60 plates (0.20 mm thick, 60F254. Merck, Germany) and observed under UV light at 254 nm or 365 nm. All reactions were carried out under air, unless stated otherwise.

All ^{1}H and ^{13}C NMR spectra were recorded on either a 500 MHz Bruker AV III equipped with a DCH cryo-probe (Ava500), a 500 MHz Bruker AV IIIHD equipped with a Prodigy cryo-probe (Pro500), a 600 MHz Bruker AV IIIHD equipped with a TCI cryo-probe (Ava600) or a 400 MHz Bruker AV III equipped with BBFO+ probe (Ava400) at a constant temperature of 300 K. Chemical shifts are reported in parts per million (ppm). Coupling constants (J) are reported in hertz (Hz). Standard abbreviations indicating multiplicity were used as follows: m = multiplet, q = quartet, t = triplet, d = doublet, s = singlet, app. = apparent. Where required, peak assignments were confirmed through a range of two-dimensional techniques including, correlated spectroscopy (COSY), nuclear Overhauser effect spectroscopy (NOESY), heteronuclear single quantum correlation (HSQC) and heteronuclear multiple bond correlation (HMBC).

MS of the compounds was performed on a Synapt G2 (Waters, Manchester, UK) mass spectrometer or a Q-ToF (Micromass UK Ltd), using a nano-electrospray ionization source (ESI), controlled using Masslynx v4.1 software. All the scans in the experimental are for positive ions. Crystals of the samples were dissolved in acetonitrile at 50 µM. Prior to analysis, instruments were calibrated using a solution of sodium iodide (2 mg/mL) in 50:50 water:isopropanol. Capillary voltages were adjusted between 1.5 and 2.5 kV to optimize spray quality, while the sampling cone and the extraction cone voltage were minimised to reduce breakdown of the assemblies. Source temperature was set at 80 °C. The data was analysed using the MassLynx v4.1 software.

Magnetisation measurements were carried out on a Quantum Design SQUID MPMS-XL magnetometer at The University of Edinburgh, operating between 1.8 and 300 K for DC applied magnetic fields ranging from 0 to 5 T. Some measurements were made on the MPMS3 magnetometer at The University of Glasgow, operating between 1.8 and 300 K for DC applied magnetic fields ranging from 0 to 7 T.

2 Synthesis

Tetrahalometallates

All tetrahalometallates were prepared based on previously published methods.[1]

M = Mn, Fe, Co, Ni and Cu, X = Cl and Br.

Anion X matched in the synthesis i.e. MX_2 and Et_4NX = $CoCl_2$ and Et_4NCl. MX_2 (3 mmol) was dissolved in EtOH (30 mL) and stirred, Et_4NX (9 mmol) was then added and stirred at room temperature for 1 hour. The precipitate was then filtered and washed with cold EtOH (3 × 10 mL) and Et_2O (3 × 10 mL). The product was then dried under vacuum to yield the product. Yields in excess of 80%.

2,2':5',5'':2'',2'''-Quaterpyridine (**L**)

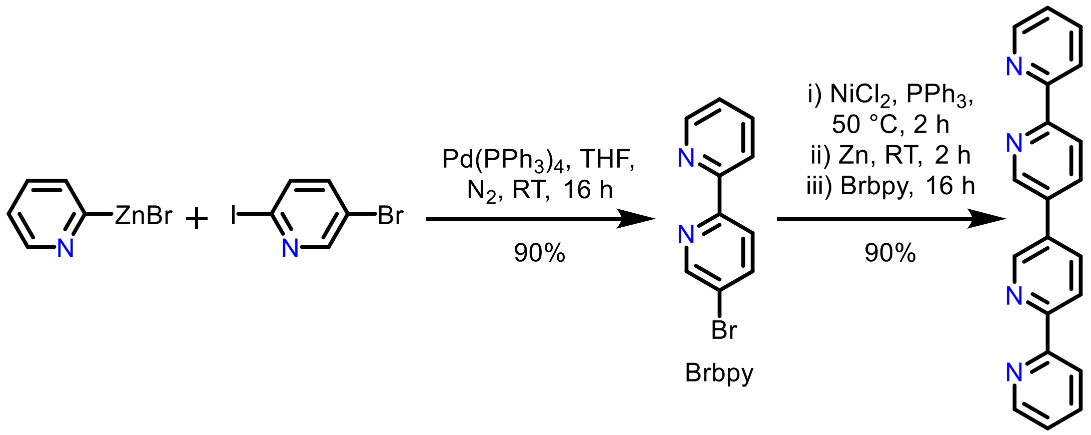

5-bromo-2,2'-bipyridine (Brbpy)

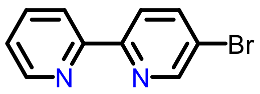

The following procedure is based on a previously published method.[2]

To a flame dried flask, 5-bromo-2-iodopyridine (11.36 g, 40 mmol) and $Pd(PPh_3)_4$ (0.462 g, 0.4 mmol) were added. Vacuum and N_2 was cycled three times before the addition 2-pyridyl zinc bromide in THF (0.5 M, 100 mL, 50.0 mmol). The resulting mixture was purged with N_2 for 15 minutes and then stirred at room temperature for 16 hours. A beige suspension formed to which 0.25 M EDTA/1 M NaOH (200 mL) and saturated Na_2CO_3 solution (100 mL) was added and stirred for 30 minutes. The resulting mixture was extracted with CH_2Cl_2 (4 × 200 mL). The combined organic phases were dried over anhydrous $MgSO_4$. Solvent was then removed *in vacuo* and

the crude product was purified by silica flash column (Hexane with 5−10% EtOAc) to give the title compound as a white solid. Yield = 8.46 g (90%). ^{1}H NMR (500 MHz, $CDCl_3$) δ (ppm): 8.72 (d, J = 2.3 Hz, 1H), 8.67 (d, J = 4.7 Hz, 1H), 8.37 (d, J = 8.0 Hz, 1H), 8.32 (d, J = 8.5 Hz, 1H), 7.94 (dd, J = 8.5, 2.4 Hz, 1H), 7.82 (td, J = 7.8, 1.8 Hz, 1 H), 7.33 (ddd, J = 7.6, 4.8, 1.1 Hz, 1H).

2,2':5',5'':2'',2'''-Quaterpyridine (**L**)

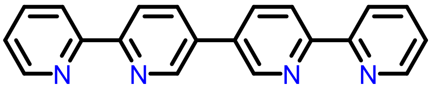

To a flame dried flask, $NiCl_2$ (2.33 g, 18 mmol) and PPh_3 (1.78 g, 6.8 mmol) were added and the vacuum and N_2 was cycled three times. Anhydrous DMF (100 mL) was then added and the mixture was heated at 50 °C until the mixture became dark blue (30 minutes−2 hours). Once cooled to room temperature Zn dust (1.21 g, 18.5 mmol) was added and stirred until dark brown (30 min−2 hours). A solution of dried Brbpy (4.00 g, 17 mmol) in anhydrous DMF (50 mL) was added to the reaction mixture via cannula and left to stir at room temperature for 16 h. The solvent was removed *in vacuo* and an EDTA (10 eq.)/NaOH (40 eq.) solution (400 mL) and CH_2Cl_2 (200 mL) was added and stirred for 16 h. The mixture was separated, and the aqueous phase was further extracted with CH_2Cl_2 (3 × 200 mL). The combined organic phases were dried over anhydrous $MgSO_4$, filtered and solvent removed *in vacuo*. The crude product was suspended in CH_3CN (50 mL) and heated to boil. Once cooled the yellow crystalline solid was filtered under vacuum and dried with Et_2O to yield the title product. Yield = 2.22 g (84%). ^{1}H NMR (500 MHz, $CDCl_3$) δ (ppm): 9.00 (dd, J = 2.4, 0.8 Hz, 2H, H_g), 8.72 (ddd, J = 4.8, 1.8, 0.9 Hz, 2H, H_a), 8.55 (dd, J = 8.2, 0.8 Hz, 2H, H_e), 8.47 (dt, J = 8.0, 1.0 Hz, 2H,H_d), 8.10 (dd, J = 8.2, 2.4 Hz, 2H, H_f), 7.86 (app. td, J = 7.8, 1.8 Hz, 2H, H_c), 7.35 (ddd, 7.6, 4.8, 1.2 Hz, 2H, H_b).

Host-Guest Complexes

[OTf⊂$Ni^{II}{}_4$**L**$_6$]$(OTf)_7$ (**1**)

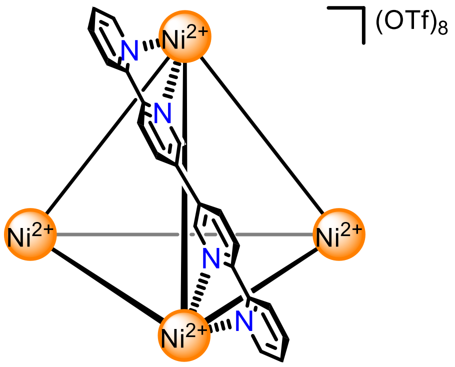

L (46.6 mg, 0.15 mmol) and $Ni(OTf)_2$ (35.7 mg, 0.1 mmol) were suspended in CH_3CN (6.0 mL) in a microwave vial. The vessel was sealed and heated at 90 °C for 1 day. The orange solution was filtered and crystallisations were set up by vapour diffusion in THF yielding dark orange X-ray quality crystals. Yields varied between 60–80%. ESI-MS (*m/z*): 1495 (2+), 947 (3+), 673 (4+), 509 (5+), 399 (6+), 321 (+7).

[$M^{II/III}X_4$⊂$Ni^{II}{}_4$**L**$_6$]$(OTf)_{6/7}$ (**2–8**)

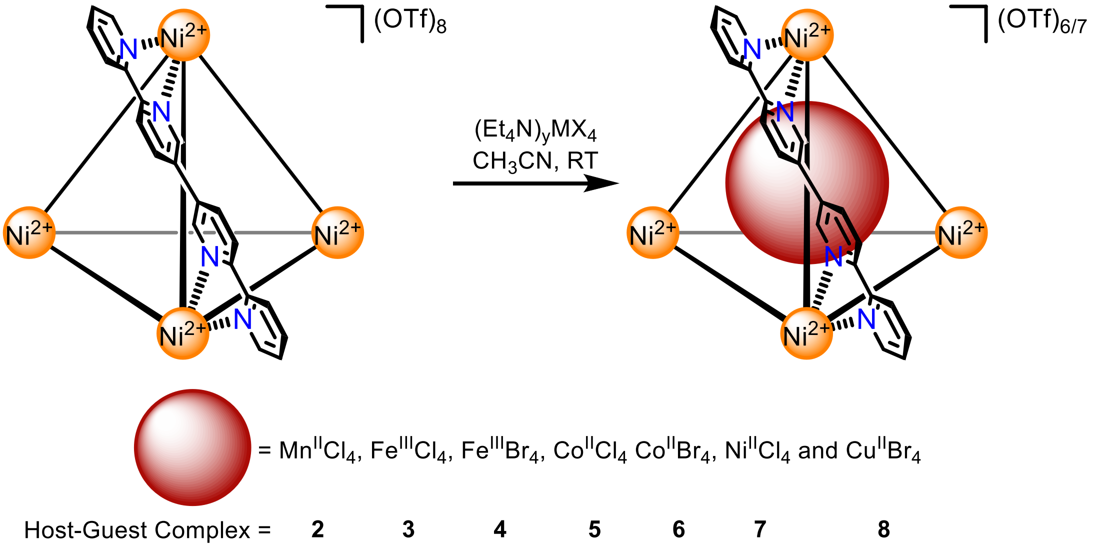

In preparation of the host-guest complexes, the synthesis of **1** was repeated, however, before crystallisations were set up the guests were added in CH_3CN (3 mL) and stirred for 5 minutes. Crystallisations via vapour diffusion were set up in Et_2O and THF (THF yielded larger crystals). Yields varied between 60–80%. Due to the large voids in the structure containing solvent of crystallisation, elemental analysis could not be performed accurately.

$[Mn^{II}Cl_4 \subset Ni^{II}_4\mathbf{L}_6](OTf)_6$ (**2**)

$[Et_4N]_2[Mn^{II}Cl_4]$ (12.0 mg, 0.025 mmol) was added and the resultant solution became light orange in colour. Yielded orange X-ray quality crystals. ESI-MS (*m/z*): 1445 (2+), 913 (3+), 648 (4+), 489 (5+), 382 (6+).

$[Fe^{III}Cl_4 \subset Ni^{II}_4\mathbf{L}_6](OTf)_7$ (**3**)

$[Et_4N][Fe^{III}Cl_4]$ (9.0 mg, 0.025 mmol) was added and the resultant solution became dark orange in colour. Yielded red X-ray quality crystals. ESI-MS (*m/z*): 1520 (2+), 963 (3+), 685 (4+), 518 (5+), 407 (6+), 328 (7+).

$[Fe^{III}Br_4 \subset Ni^{II}_4\mathbf{L}_6](OTf)_7$ (**4**)

$[Et_4N][Fe^{III}Br_4]$ (13.0 mg, 0.025 mmol) was added and the resultant solution became red in colour. Yielded dark red X-ray quality crystals. ESI-MS (*m/z*): 1608 (2+), 1023 (3+), 740 (4+), 554 (5+), 437 (6+), 353 (7+).

$[Co^{II}Cl_4 \subset Ni^{II}_4\mathbf{L}_6](OTf)_6$ (**5**)

$[Et_4N]_2[Co^{II}Cl_4]$ (12.0 mg, 0.025 mmol) was added and the resultant solution became green in colour. Yielded green X-ray quality crystals. ESI-MS (*m/z*): 1447 (2+), 915 (3+), 649 (4+), 489 (5+), 383 (6+).

$[Co^{II}Br_4 \subset Ni^{II}_4\mathbf{L}_6](OTf)_6$ (**6**)

$[Et_4N]_2[Co^{II}Br_4]$ (16.0 mg, 0.025 mmol) was added and the resultant solution became light orange in colour. Yielded light green X-ray quality crystals. ESI-MS (*m/z*): 1536 (2+), 974 (3+), 693 (4+), 525 (5+), 413(6+).

$[Ni^{II}Cl_4 \subset Ni^{II}_4\mathbf{L}_6](OTf)_6$ (**7**)

$[Et_4N]_2[Ni^{II}Cl_4]$ (12.0 mg, 0.025 mmol) was added and the resultant solution became green in colour. Yielded light green X-ray quality crystals. ESI-MS (*m/z*): 1446 (2+), 914(3+), 649 (4+), 489 (5+), 383 (6+).

$[Cu^{II}Br_4 \subset Ni^{II}_4\mathbf{L}_6](OTf)_6$ (**8**)

$[Et_4N]_2[Cu^{II}Br_4]$ (16.0 mg, 0.025 mmol) was added and the resultant solution became dark green in colour. Yielded dark green X-ray quality crystals. ESI-MS (*m/z*): 1537 (2+), 975 (3+), 694 (4+), 526 (5+), 413 (6+).

3 Single-Crystal-to-Single-Crystal Transformation of 1↔5

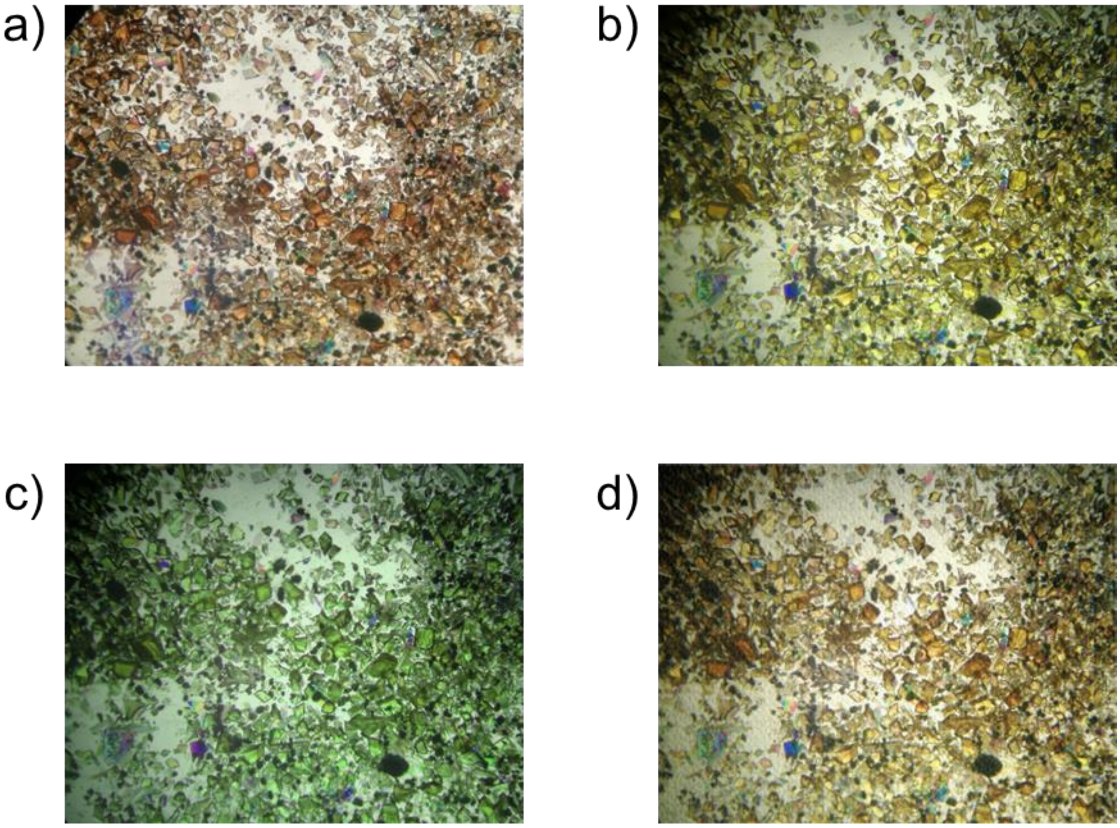

Fig. S1 - a) Crystals of **1** in EtOH. b) Crystals of **1** with 5 equivalents of $[Et_4N]_2CoCl_4$ in EtOH added. Picture taken after 30 minutes, with a colour change evident. c) After two hours at room temperature the crystals have changed colour from yellow-brown to green single crystal XRD confirms transformation to complex **5.** d) Crystals of **5** formed from the initial SCSC soaked in a saturated EtOH solution of $[nBu_4N][OTf]$ after 4 hours at 40 °C. Single crystal XRD confirms complete transformation to complex **1**, as also indicated from the colour change back to yellow-brown.

4 Mass Spectrometry

[Ni^{II}_4**L**$_6$]$(OTf)_8$ (**1**)

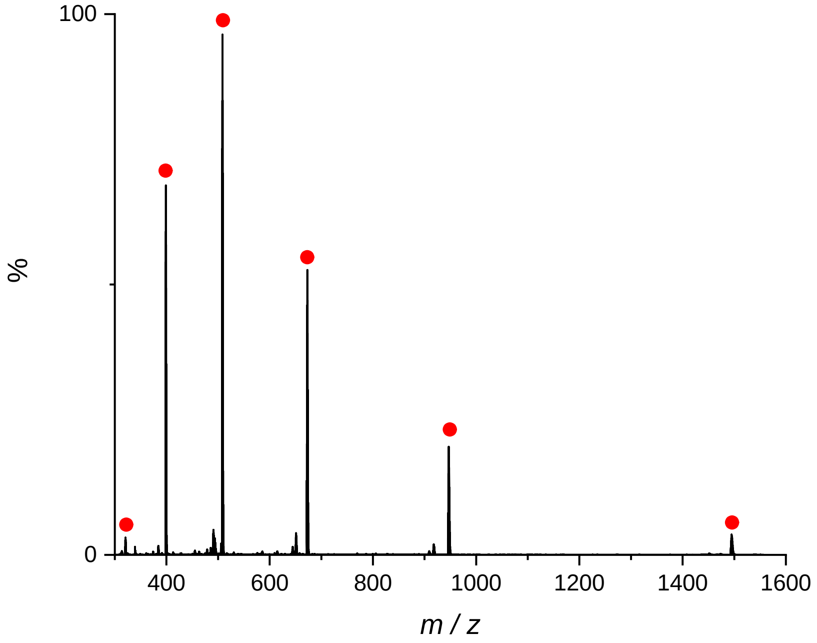

Fig. S2 - Mass spectrum of 1. Red dots indicating the charge states of the cage from +7 on the left to +2 on the right.

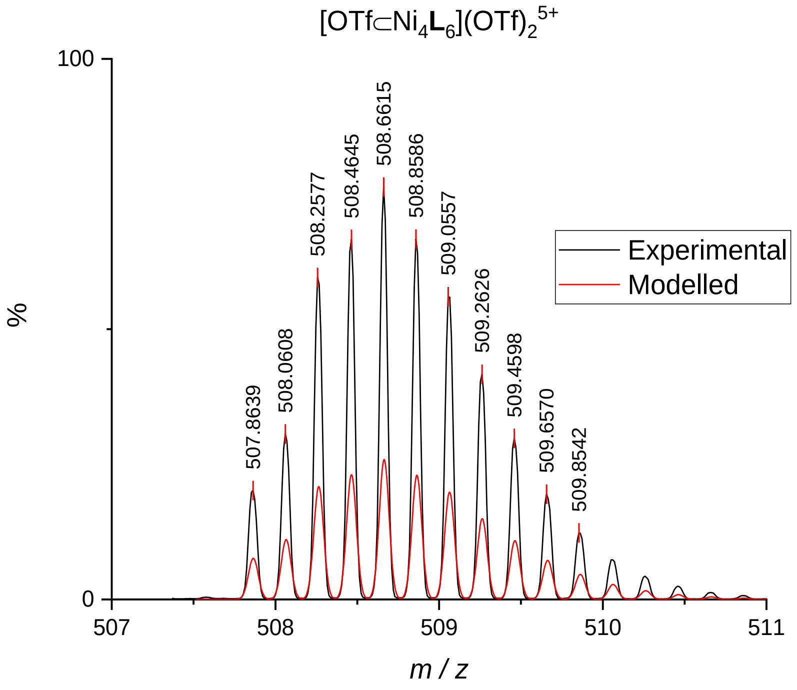

Fig. S3 - The observed +5 charge state for **1**. Black line indicates the experimental pattern and the red line indicating the modelled.

$[Mn^{II}Cl_4 \subset Ni^{II}{}_4\mathbf{L}_6](OTf)_6$ (**2**)

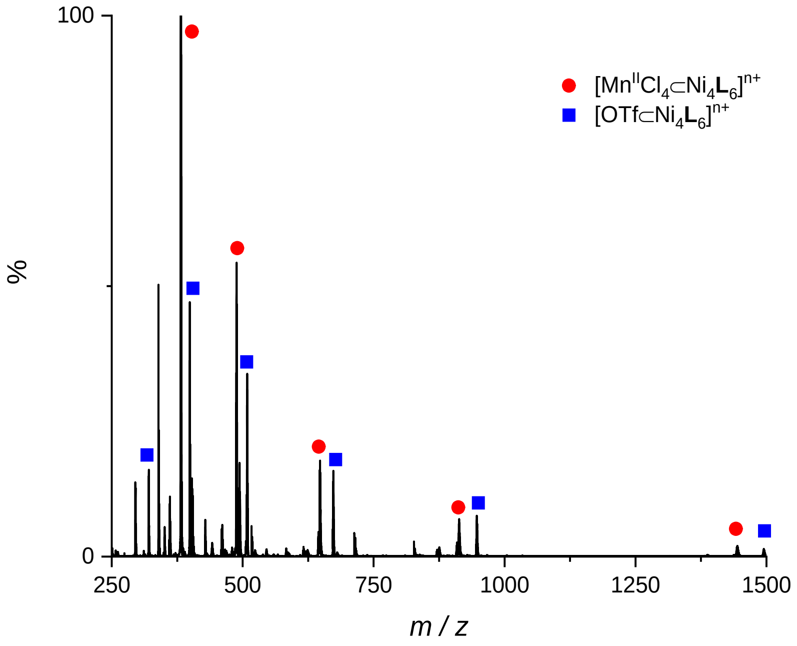

Fig. S4 - Mass spectrum of **2**. Red dots indicating the charge states of the cage from +6 on the left to +2 on the right, blue dots are of complex **1**.

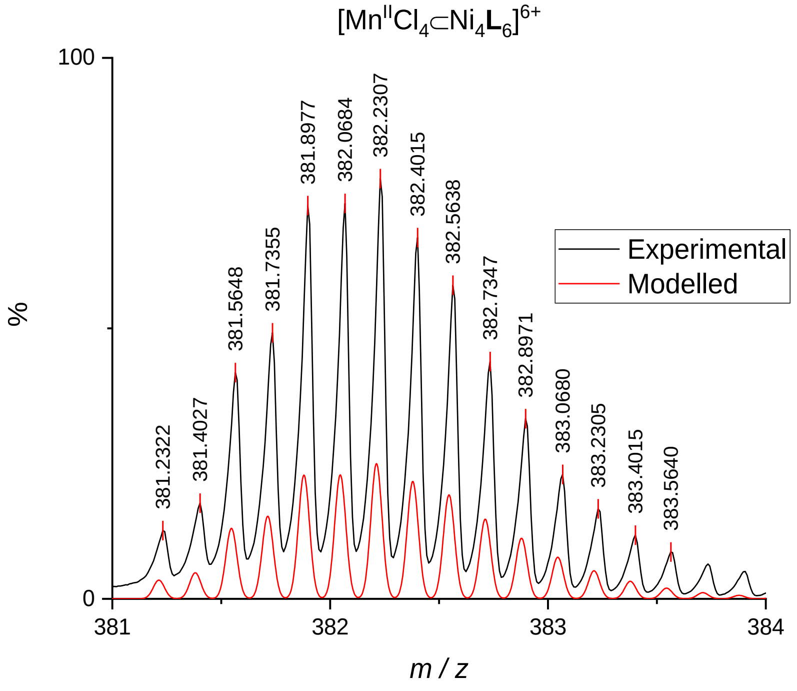

Fig. S5 - The observed +6 charge state for **2**. Black line indicates the experimental pattern and the red line indicating the modelled.

$[Fe^{III}Cl_4 \subset Ni^{II}_4\mathbf{L}_6](OTf)_7$ (**3**)

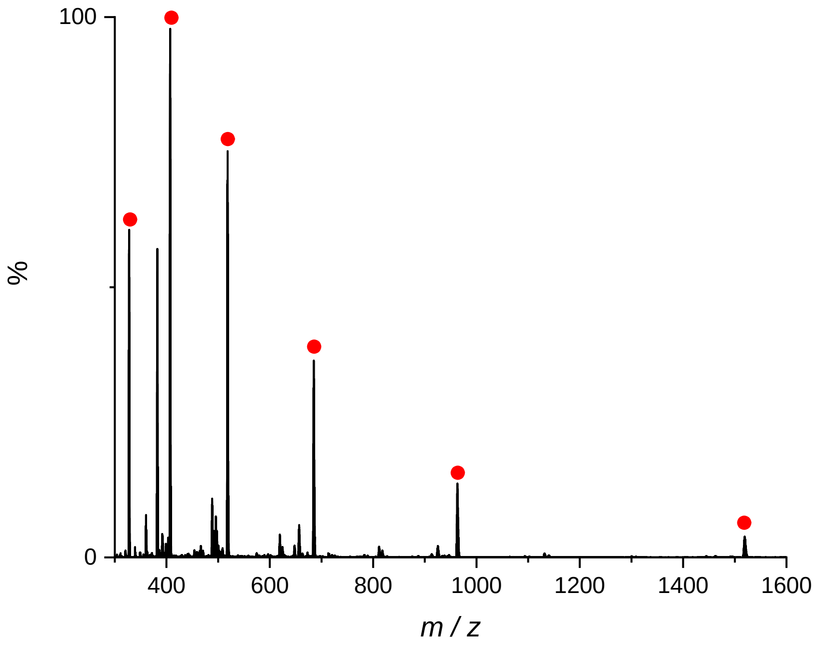

Fig. S6 - Mass spectrum of **3**. Red dots indicating the charge states of the cage from +7 on the left to +2 on the right.

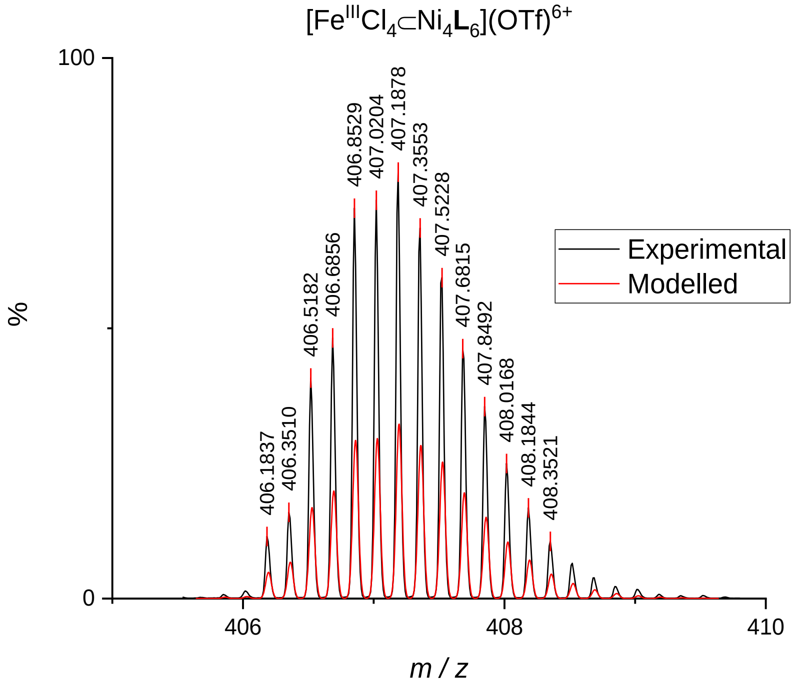

Fig. S7 - The observed +6 charge state for **3**. Black line indicates the experimental pattern and the red line indicating the modelled.

$[Fe^{III}Br_4 \subset Ni^{II}_4\mathbf{L}_6](OTf)_7$ (**4**)

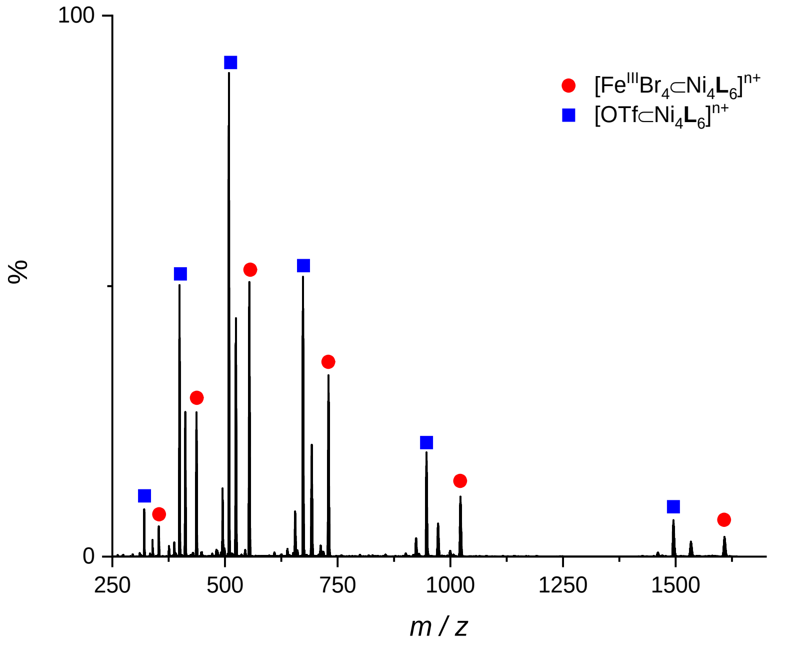

Fig. S8 - Mass spectrum of **4**. Red dots indicating the charge states of the cage from +7 on the left to +2 on the right, blue dots are of complex **1**.

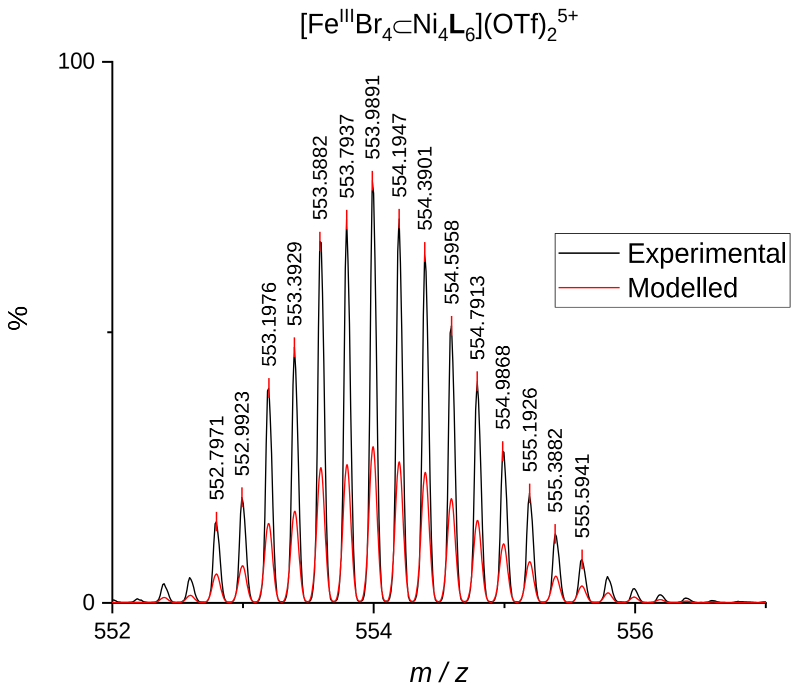

Fig. S9 - The observed +5 charge state for **4**. Black line indicates the experimental pattern and the red line indicating the modelled.

$[Co^{II}Cl_4 \subset Ni^{II}_4\mathbf{L}_6](OTf)_6$ (**5**)

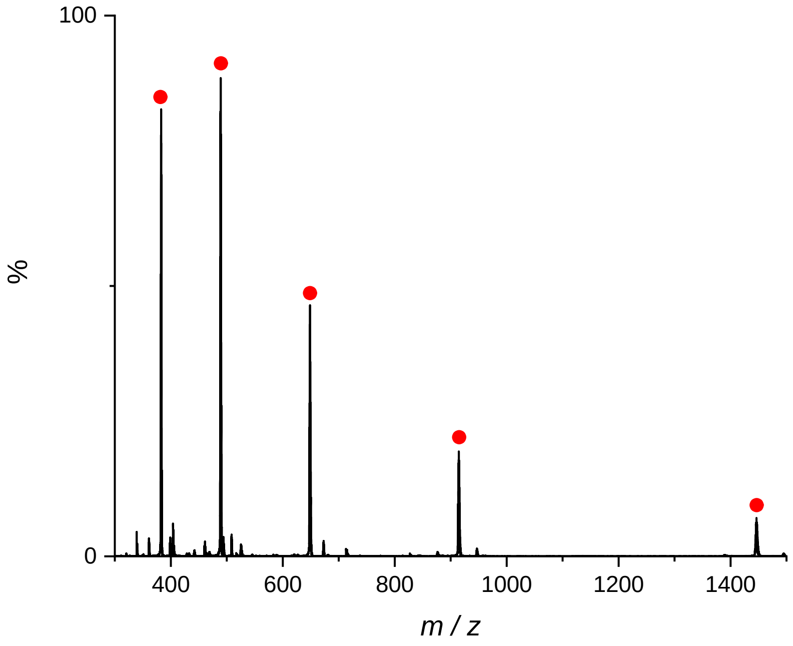

Fig. S10 - Mass spectrum of **5**. Red dots indicating the charge states of the cage from +6 on the left to +2 on the right.

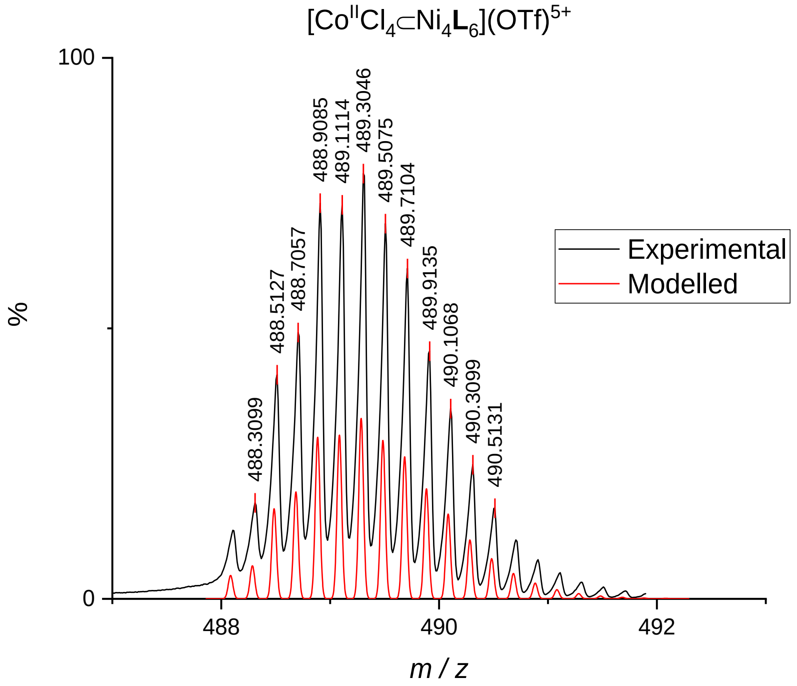

Fig. S11 - The observed +5 charge state for **5**. Black line indicates the experimental pattern and the red line indicating the modelled.

[$Co^{II}Br_4 \subset Ni^{II}{}_4$**L**$_6$]$(OTf)_6$ (**6**)

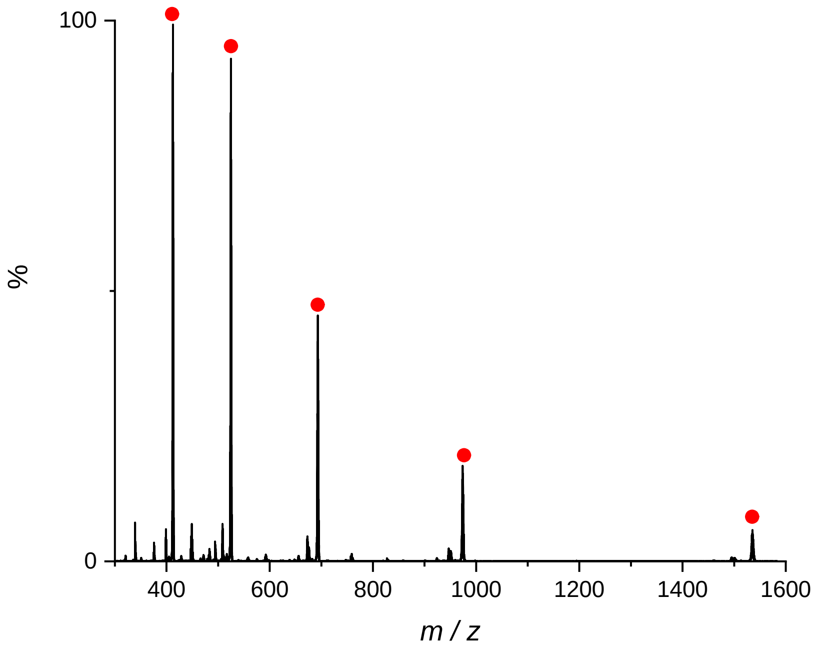

Fig. S12 - Mass spectrum of **6**. Red dots indicating the charge states of the cage from +6 on the left to +2 on the right.

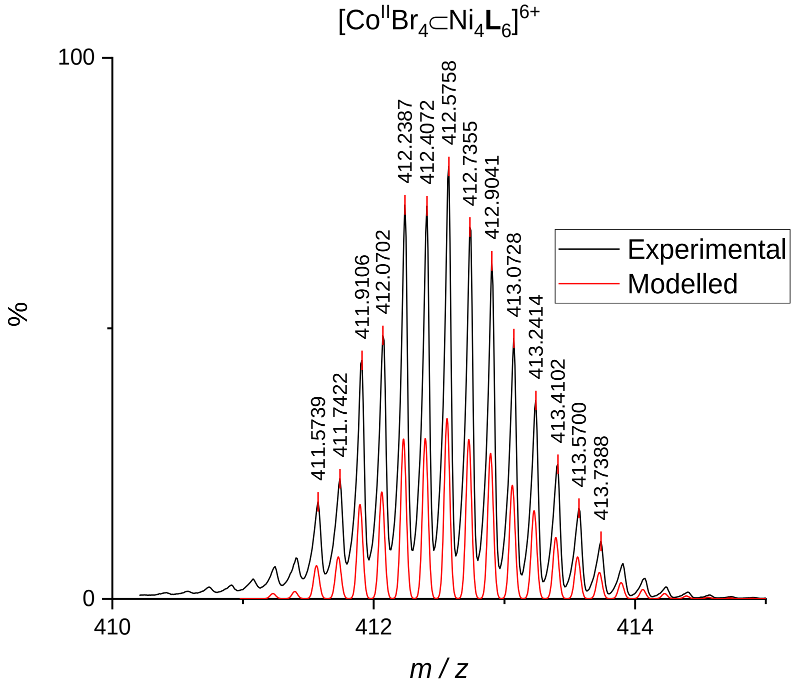

Fig. S13 - The observed +6 charge state for **6**. Black line indicates the experimental pattern and the red line indicating the modelled.

[$Ni^{II}Cl_4 \subset Ni^{II}{}_4$**L**$_6$]$(OTf)_6$ (**7**)

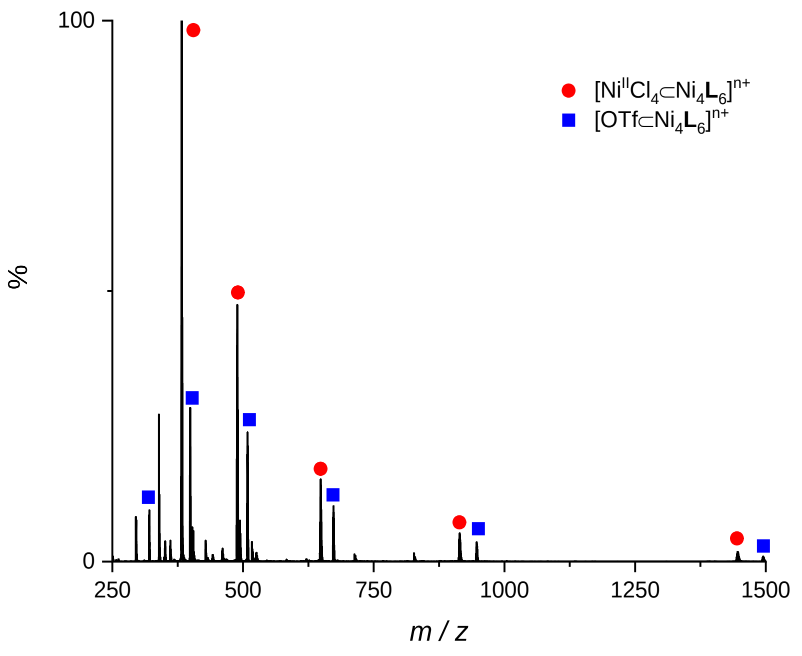

Fig. S14 - Mass spectrum of **7**. Red dots indicating the charge states of the cage from +6 on the left to +2 on the right, blue dots are of complex **1**.

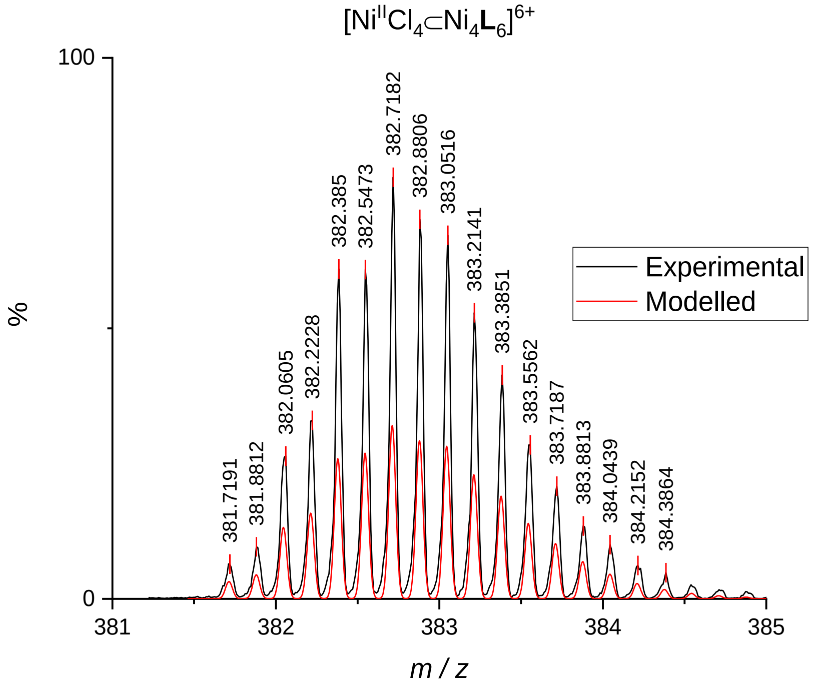

Fig. S15 - The observed +6 charge state for **7**. Black line indicates the experimental pattern and the red line indicating the modelled.

[$Cu^{II}Br_4 \subset Ni^{II}_4$**L**$_6$]$(OTf)_6$ (**8**)

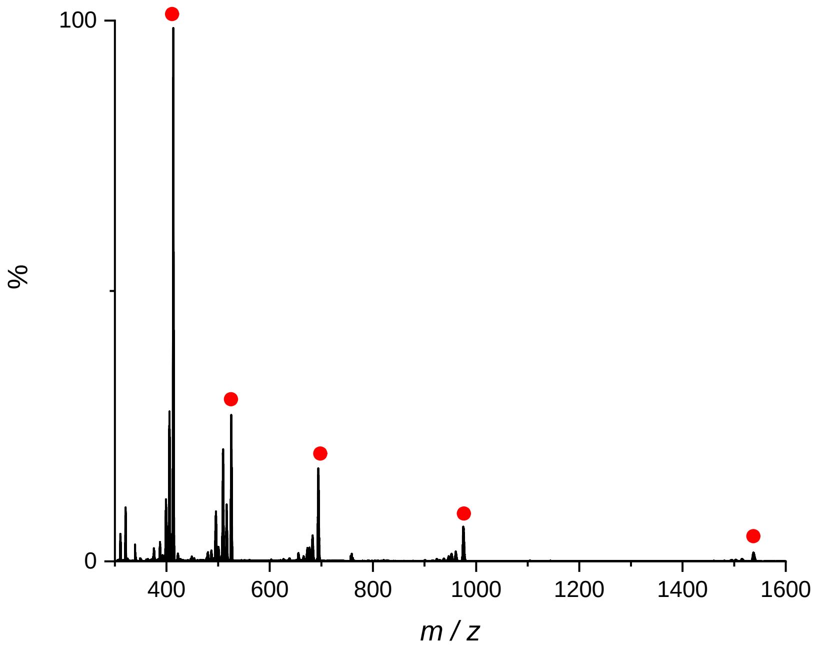

Fig. S16 - Mass spectrum of **8**. Red dots indicating the charge states of the cage from +6 on the left to +2 on the right.

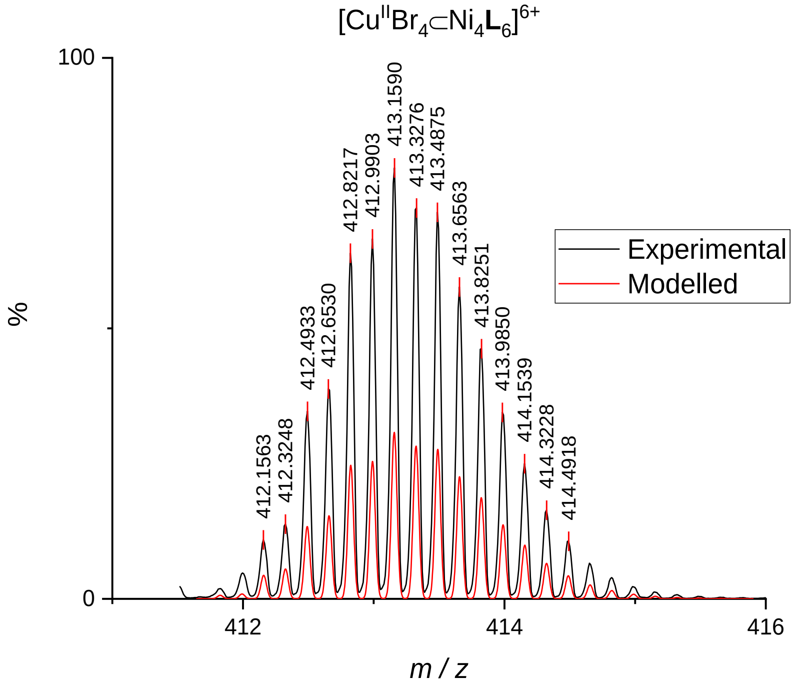

Fig. S17 - The observed +6 charge state for **8**. Black line indicates the experimental pattern and the red line indicating the modelled.

5 Crystallography

General Remarks

[$OTf\subset Ni^{II}_4$**L**$_6$]$(OTf)_7$ (**1**), [$Fe^{III}Cl_4\subset Ni^{II}_4$**L**$_6$]$(OTf)_7$ (**3**), [$Fe^{III}Br_4\subset Ni^{II}_4$**L**$_6$]$(OTf)_7$ (**4**) and [$Co^{II}Cl_4\subset Ni^{II}_4$**L**$_6$]$(OTf)_6$ (**5**).

Single crystal X-ray diffraction data for samples **1**, and **3–5** were collected using a Rigaku Oxford Diffraction SuperNova diffractometer with CuKα (**1** and **5**) and MoKα (**3** and **4**) radiation. An Oxford Cryosystems Cryostream 700+[3] low temperature device was used to maintain a crystal temperature of 120.0 K (**1, 3** and **4**) and 250.0 K (**5**). The CrysAlisPro software package was used for instrument control, unit cell determination and data reduction.

The structures were solved using ShelXT[4] employing the Intrinsic Phasing solution method through Olex2[5] as the graphical interface. The model was refined with ShelXL[6] using Least Squares minimisation. All non-hydrogen atoms were refined with anisotropic displacement parameters. Hydrogen atom positions were calculated geometrically and refined using the riding model. The RIGU restraint was applied to all triflate anions to appropriately model atomic displacement parameters.

All crystal structures contain large accessible voids that are filled with diffuse electron density belonging to disordered solvent, whose electron contribution was masked using the SQUEEZE[7] routine of PLATON[8]. This missing solvent is included in the total formula, triggering checkCIF alerts which should be ignored.

[$Mn^{II}Cl_4\subset Ni^{II}_4$**L**$_6$]$(OTf)_6$ (**2**), [$Ni^{II}Cl_4\subset Ni^{II}_4$**L**$_6$]$(OTf)_6$ (**7**) and [$Cu^{II}Br_4\subset Ni^{II}_4$**L**$_6$]$(OTf)_6$ (**8**).

Single crystal X-ray diffraction data for **2**, **7** and **8** were collected remotely[9] at Diamond Light Source, beamline I19-1,[10] under beam time award CY22240. An Oxford Cryosystems Cryostream 700+ low temperature device was used to maintain a crystal temperature of 100.0 K (**2** and **8**) and 120.0 K (**7**). The diffraction patterns were indexed with Xia2[11–13]. The structures were solved, refined, and disordered solvent masked as mentioned for **1** and **3–5**.

$[Co^{II}Br_4 \subset Ni^{II}_4$**L**$_6](OTf)_6$ (**6**)

Single crystal X-ray diffraction data for **6** were collected using a Rigaku FRE+ diffractometer with MoKα radiation. An Oxford Cryosystems Cryostream 700+ low temperature device was used to maintain a crystal temperature of 100.0 K.

The structures were solved, refined, and disordered solvent masked as mentioned for **1** and **3–5**.

Table S1 - Single crystal X-ray data for complexes **1−4**.

	**1**	**2**	**3**	**4**
**Formula**	$C_{132}H_{88}F_{24}N_{26}Ni_4O_{24}S_8$	$C_{126}H_{84}Cl_4F_{18}MnN_{24}Ni_4O_{18}S_6$	$C_{151}H_{120}Cl_4F_{21}FeN_{36}Ni_4O_{21}S_7$	$C_{147.5}H_{124}Br_2F_{24}Fe_{0.5}N_{24}Ni_4O_{29}S_{7.5}$
**M (g mol^{-1})**	3369.60	3188.11	3830.73	3815.73
**Crystal Colour**	Orange	Light brown	Dark red	Dark red
**Crystal system**	Monoclinic	Monoclinic	Monoclinic	Monoclinic
**Space group**	C2/*c*	C2/*c*	C2/*c*	C2/*c*
**a (Å)**	31.7631(4)	32.5915(13)	31.6627(5)	31.3026(3)
**b (Å)**	20.3760(3)	19.6893(7)	20.3859(2)	20.5660(2)
**c (Å)**	26.2656(4)	26.8651(12)	26.5585(4)	26.4213(3)
**α (°)**	90	90	90	90
**β (°)**	114.2627(18)	116.807(3)	114.491(2)	112.8120(10)
**γ (°)**	90	90	90	90
**V (Å^3)**	15497.7(4)	15386.7(11)	15600.4(4)	15678.8(3)
**Z**	4	4	4	4
**Z'**	0.5	0.5	0.5	0.5
**ρ_{calc} (g cm^{-3})**	1.444	1.376	1.631	1.616
**μ (mm^{-1})**	2.438	0.751	0.833	1.236
**T (K)**	120.0	100.0	120.0	120.0
**F(000)**	6832	6460.0	6932.0	7760.0
**Measured Reflections**	156152	29061	238777	242243
**Independent Reflections (R_{int})**	16138(0.0786)	3672 (0.1060)	14267(0.0552)	14855(0.0536)
**Reflections [$I > 2\sigma(I)$]**	13435	3008	12312	12951
**Data / Restraints / Parameters**	16138 / 216 / 911	3672 / 77 / 402	14267 / 197 / 1006	14855 / 656 / 1179
**$R_1{}^a$ [$I > 2\sigma(I)$] (all)**	0.1254(0.1363)	0.2397(0.2527)	0.0655(0.0749)	0.0729(0.0820)
**$wR_2{}^b$ [$I > 2\sigma(I)$] (all)**	0.3432(0.3525)	0.5481(0.5660)	0.1774(0.1864)	0.2032(0.2106)
**Goodness-of-fit**	2.759	5.029	1.042	1.068
**CCDC Number**	2024431	2024429	2024434	2024432

Table S2 - Single crystal X-ray data for complexes **5−8**.

	**5**	**6**	**7**	**8**
**Formula**	$C_{138}H_{105}Cl_4CoF_{18}N_{28}Ni_4O_{19}S_6$	$C_{130}H_{93}Br_4CoF_{12}N_{27}Ni_4O_{12}S_4$	$C_{154}H_{126}Cl_4F_{18}N_{38}Ni_5O_{18}S_6$	$C_{126}H_{84}Br_4CuF_{18}N_{24}Ni_4O_{18}S_6$
M **(g mol^{-1})**	3429.42	3194.96	3766.63	3374.55
**Crystal Colour**	Dark green	Light brown	Light green	Dark green
**Crystal System**	Monoclinic	Monoclinic	Monoclinic	Monoclinic
**Space Group**	$P2_1/n$	$P2_1/c$	C2/*c*	C2/*c*
a **(Å)**	20.4016(3)	31.1965(6)	31.8614(3)	33.6581(8)
b **(Å)**	29.7181(6)	18.5947(3)	20.2670(2)	19.3004(5)
c **(Å)**	26.8106(6)	28.7563(6)	26.4739(3)	27.7984(9)
**α (°)**	90	90	90	90
**β (°)**	91.1568(18)	110.013(2)	114.7730(10)	119.638(2)
**γ (°)**	90	90	90	90
V **(Å^3)**	16251.9(5)	15673.9(5)	15521.9(3)	15695.6(8)
Z	4	4	4	4
Z'	1	1	0.5	0.5
**ρ_{calc} (g cm^{-3})**	1.402	1.354	1.612	1.428
**μ (mm^{-1})**	3.379	1.723	0.781	1.643
T **(K)**	250.0(10)	100.0(2)	100.0	120.0
F**(000)**	6980.0	6436.0	7000.0	6764.0
**Measured Reflections**	294893	670814	94775	49607
**Independent Reflections (R_{int})**	17006(0.1547)	35971(0.0697)	14171(0.0404)	6192(0.0617)
**Reflections [$I > 2\sigma(I)$]**	14654	23303	11094	5165
**Data / Restraints / Parameters**	17006 / 2169 / 1962	35971 / 0 / 1750	14171 / 108 / 990	6192 / 747 / 678
**$R_1{}^a$ [$I > 2\sigma(I)$] (all)**	0.1312(0.1392)	0.0744(0.1120)	0.1274(0.1396)	0.1602(0.1669)
**$wR_2{}^b$ [$I > 2\sigma(I)$] (all)**	0.3722(0.3828)	0.1485(0.1673)	0.3680(0.3778)	0.4648(0.4780)
**Goodness-of-fit**	1.712	1.025	3.070	2.397
**CCDC Number**	2024435	2023466	2024430	2024433

Data Tables

Table S3 - NiN_6 and MX_4 bond length and angle ranges for complexes **1–8**

	**Ni–N Bond Lengths (Å)**	**N–Ni–N *cis* (°)**	**N–Ni–N *trans* (°)**	**M–X Bond Lengths (Å)**	**X–M–X (°)**
**T1**	2.052(4)–2.134(4)	77.87(14)–100.56(15)	170.75(16)–175.70(15)		
**T2**	2.03(3)–2.17(3)	76.4(12)–99.8(12)	170.3(11)–175.8(12)	2.378(12)–2.383(12)	105.9(6)–113.4(4)
**T3**	2.057(2)–2.128(3)	78.19(9)–99.89(9)	171.52(10)–175.37(10)	2.1946(8)–2.2060(7)	106.32(4)–112.33(3)
**T4**	2.055(4)–2.131(4)	77.85(16)–100.74(16)	171.37(16)–175.62(16)	2.3415(18)–2.3511(18)	105.29(11)–112.99(5)
**T5**	2.050(3)–2.130(3)	78.10(14)–102.28(13)	170.56(13)–176.60(13)	2.272(3)–2.281(3)	104.99(13)–114.28(11)
**T6**	2.053(5)–2.105(4)	78.20(18)–100.00(2)	171.59(19)–173.85(19)	2.4034(8)–2.4242(8)	107.05(3)–111.07(3)
**T7**	2.059(5)–2.131(5)	78.4(2)–100.3(2)	170.6(2)–175.4(2)	2.257(4)–2.262(4)	101.7(2)–117.66(12)
**T8**	2.051(9)–2.077(14)	77.3(6)–100.5(6)	171.0(5)–176.6(6)	2.584(7)–2.359(5)	107.25(10)–117.8(3)

Table S4 - Pore volumes, diameters and average window diameters of complexes **1–8**. Calculated using pywindow.[14]

	**Pore Volume /Å^3**	**Pore Diameter /Å**	**Average Window Diameter /Å**
**T1**	62.6	4.9	3.4
**T2**	67.9	5.1	3.6
**T3**	68.7	5.1	3.6
**T4**	68.0	5.1	3.6
**T5**	64.2	5.0	3.6
**T6**	80.8	5.4	3.3
**T7**	62.5	4.9	3.4
**T8**	70.6	5.1	3.5

Table S5 - Close intermolecular interactions of complexes **1–8** between the cages, ions and solvent of crystallisation.

	**Short Contacts /Å**		
	**Guest···Cage**	**External Ions**	**External Solvent**
**T1**	O/F···H−Ar ≈ 2.52	F···H−Ar ≈ 2.67 (OTf···Cage)<br>O···H−Ar ≈ 2.46(OTf···Cage)	H_2C−H···C−Ar ≈ 2.85 (MeCN···Cage)<br>N···H−Ar ≈ 2.56 (MeCN···Cage)
**T2**	Cl···H−Ar ≈ 2.95	F···H−Ar ≈ 2.37 (OTf···Cage)<br>O···H−Ar ≈ 2.50 (OTf···Cage)	–
**T3**	Cl···H−Ar ≈ 2.99	F···H−Ar ≈ 2.53 (OTf···Cage)<br>O···H−Ar ≈ 2.54 (OTf···Cage)	H_2C−H···C−Ar ≈ 2.85 (MeCN···Cage)<br>N···H−Ar ≈ 2.57 (MeCN···Cage)
**T4**	Br···H−Ar ≈ 3.03	F···H−Ar ≈ 2.50 (OTf···Cage)<br>O···H−Ar ≈ 2.53 (OTf···Cage)<br>O···H−CH ≈ 2.59 (OTf···THF)<br>F···H−CH ≈ 2.48 (OTf···THF)	HC−H···Br ≈ 2.78 (THF···Br)<br>O···H−Ar ≈ 2.60 (THF···Cage)<br>HC−H···H−Ar ≈ 2.59 (THF···Cage)
**T5**	Cl···H−Ar ≈ 2.99	F···H−Ar ≈ 2.54 (OTf···Cage)<br>O···H−Ar ≈ 2.53 (OTf···Cage)<br>O···H−CH ≈ 2.57 (OTf···THF)<br>F···H−CH ≈ 2.62 (OTf···THF)	HC−H···C−Ar ≈ 2.56 (THF···Cage)<br>HC−H···H−Ar ≈ 1.93 (THF···Cage)<br>H_2C−H···H−Ar ≈ 1.75 (MeCN···Cage)<br>N···H−Ar ≈ 2.63 (MeCN···Cage)
**T6**	Br···H−Ar ≈ 3.04	F···H−Ar ≈ 2.54 (OTf···Cage)<br>O···H−Ar ≈ 2.47 (OTf···Cage)<br>O···H−CH_2 ≈ 2.60 (OTf···MeCN)<br>F···H−CH_2 ≈ 2.45 (OTf···MeCN)	HC−H···Br ≈ 2.90 (THF···Br)<br>N···H−Ar ≈ 2.58 (MeCN···Cage)
**T7**	Cl···H−Ar ≈ 2.86	F···H−Ar ≈ 2.54 (OTf···Cage)<br>O···H−Ar ≈ 2.52 (OTf···Cage)	H_2C−H···H−Ar ≈ 2.18 (MeCN···Cage)<br>H_2C−H···C−Ar ≈ 2.83 (MeCN···Cage)<br>N···H−Ar ≈ 2.53 (MeCN···Cage)
**T8**	Br···H−Ar ≈ 3.00	F···H−Ar ≈ 2.11 (OTf···Cage)<br>O···H−Ar ≈ 2.48 (OTf···Cage)	–

Structures and Packing

[OTf⊂Ni^{II}_4**L**$_6$](OTf)$_7$ (**1**)

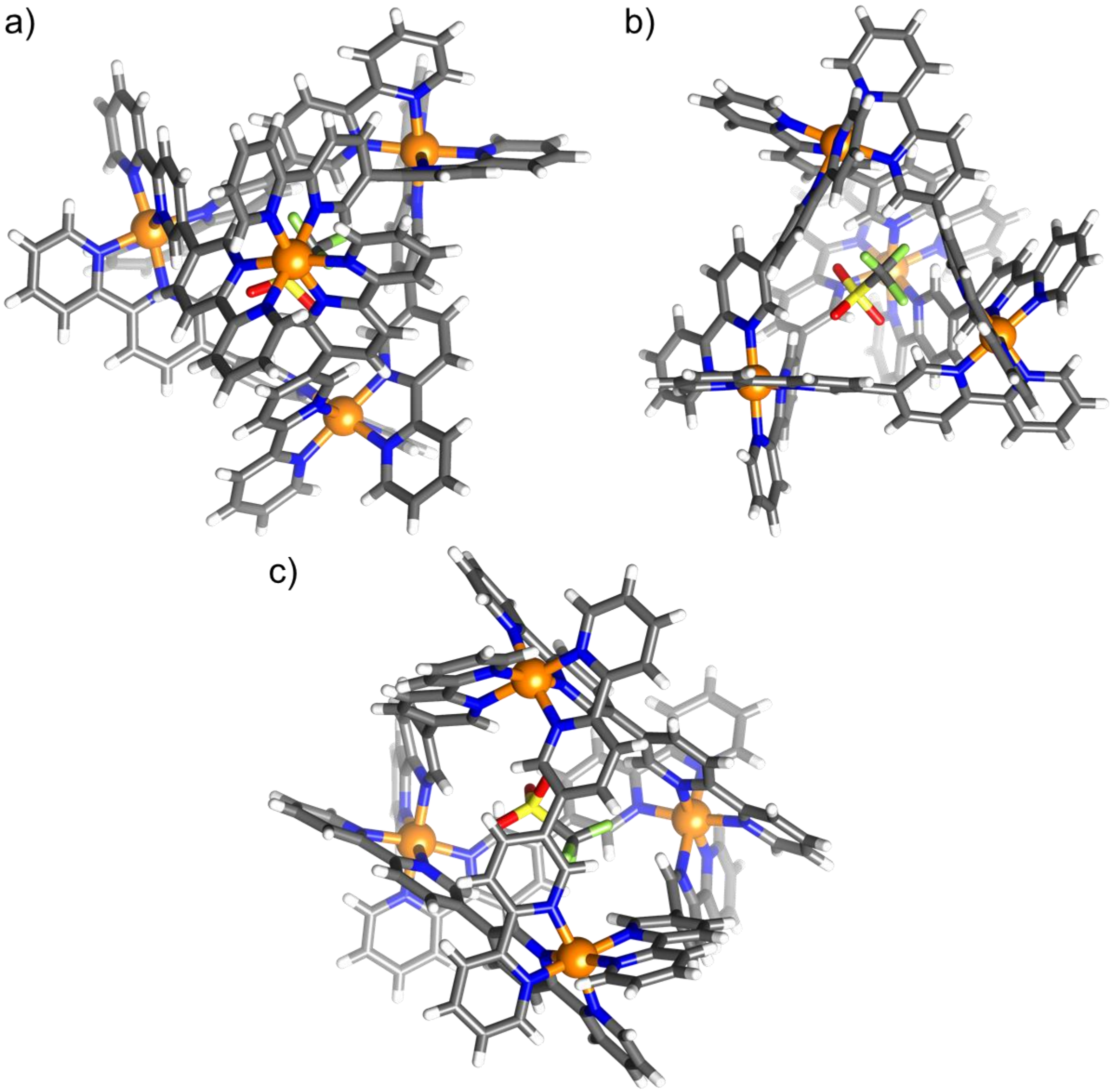

Fig. S18 - (a)-(b) Orthogonal views of complex **1** down a vertex of the cage and through the portal, the triflate anion guest is positionally disordered within the cage, with the O and F atoms point towards the portals/H atoms in the ligand framework.. (c) Side-view of **1** highlighting the connectivity of the ligand in the cage. Colour code: Colour code: Ni = orange, N = blue, C = grey, H = white, O = red, F = light green, S = yellow.

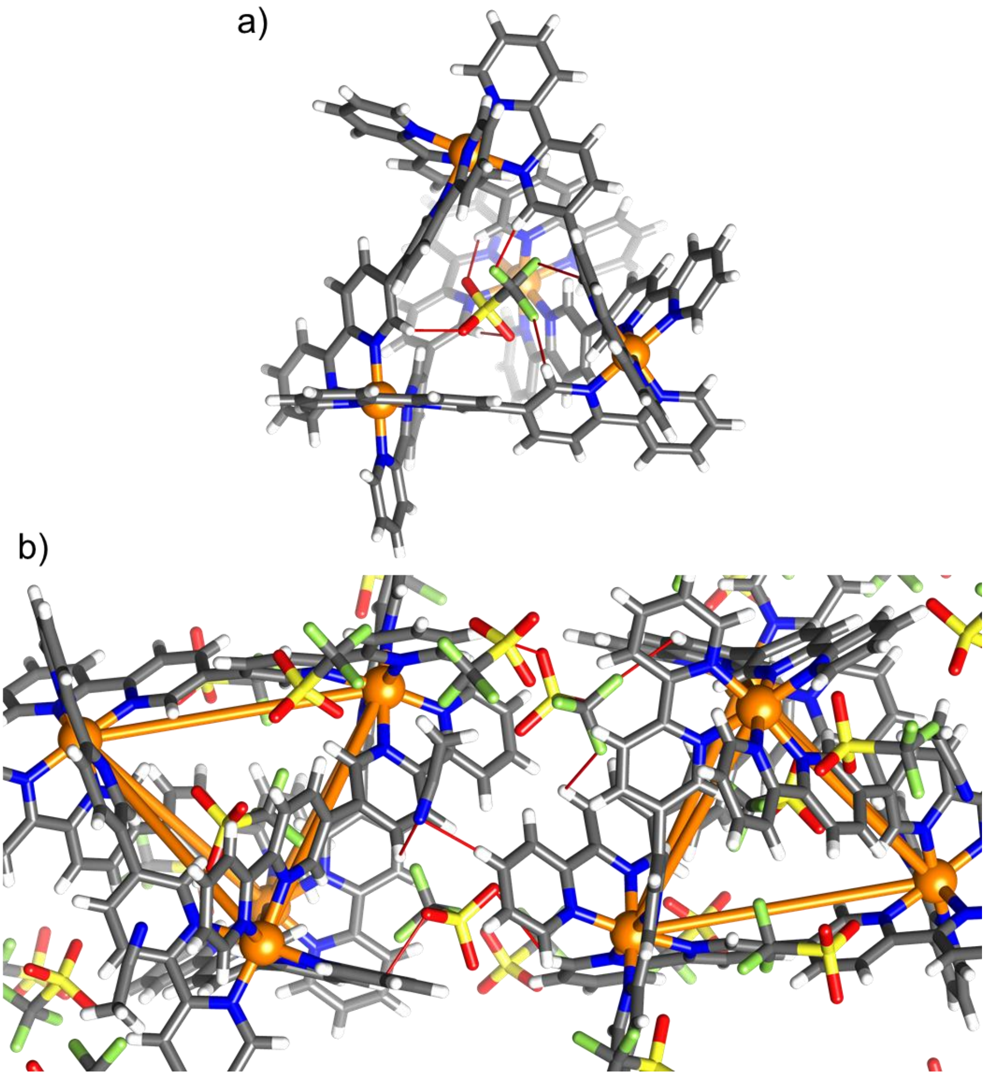

Fig. S19 - (a) Close intermolecular interactions (thin red bonds) between the O and F atoms of the encapsulated triflate anion and the H atoms of **L** in the cage framework. And (b) Two $[Ni_4(\mathbf{L1})_6]^{8+}$ tetrahedra of **1**, thin red bonds indicating some of the close intermolecular interactions from external triflate anions and solvent of crystallisation linking neighbouring cages. Orange bonds used to highlight the adjacent tetrahedra. Colour code as Fig.S18.

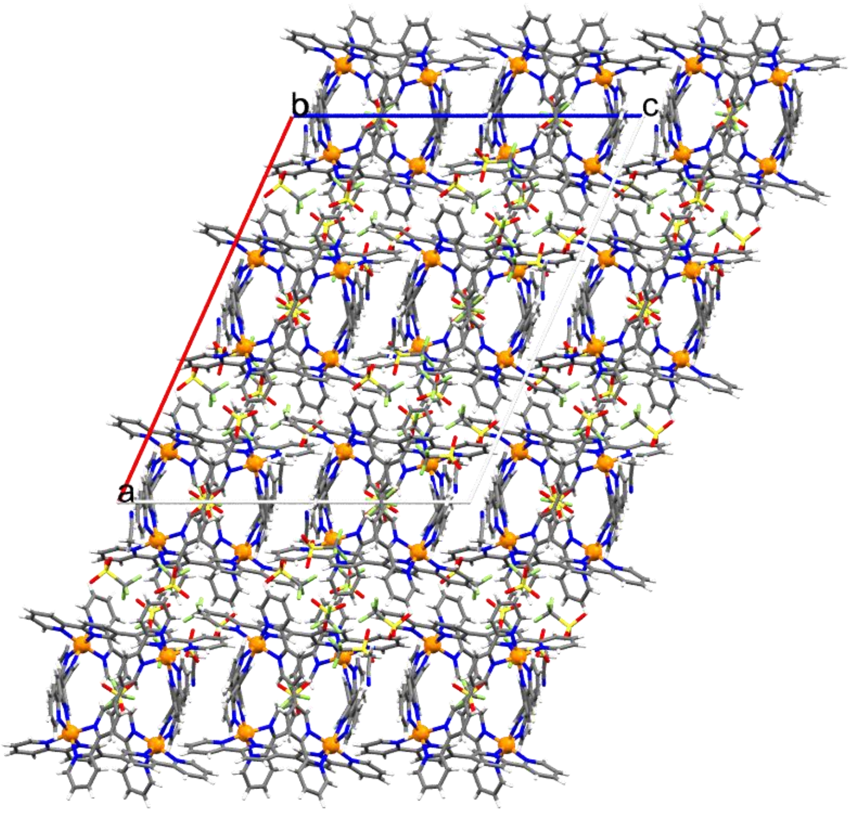

Fig. S20 - Packing of **1** (*C*2/*c*) viewed down the b-axis, illustrating the alternating rows of cages with an encapsulated triflate followed by a row of triflate anions connecting the cages. Colour code as Fig. S18.

[$Mn^{II}Cl_4 \subset Ni^{II}{}_4$**L**$_6$]$(OTf)_6$ (**2**)

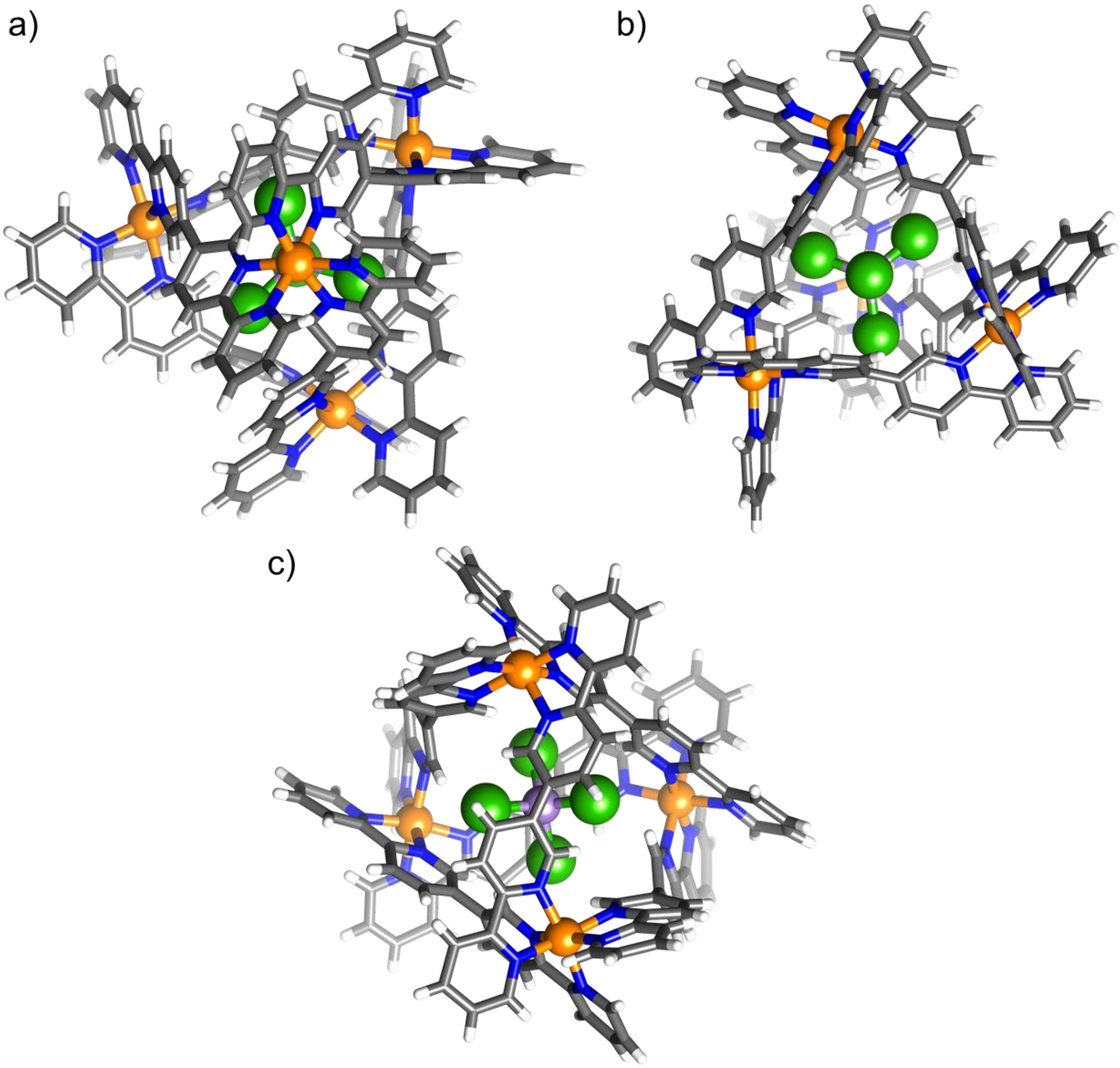

Fig. S21 - (a)-(b) Orthogonal views of complex **2** down a vertex of the cage and through the portal, illustrating the position of the $[MnCl_4]^{2-}$ guest which sits as an inverted tetrahedron with respect to the host cage, with the halide ions pointing out of the cage portals. (c) Side-view of **2** highlighting the connectivity of the ligand in the cage. Colour code: Same as Fig. S18, Cl = green, Mn = mauve.

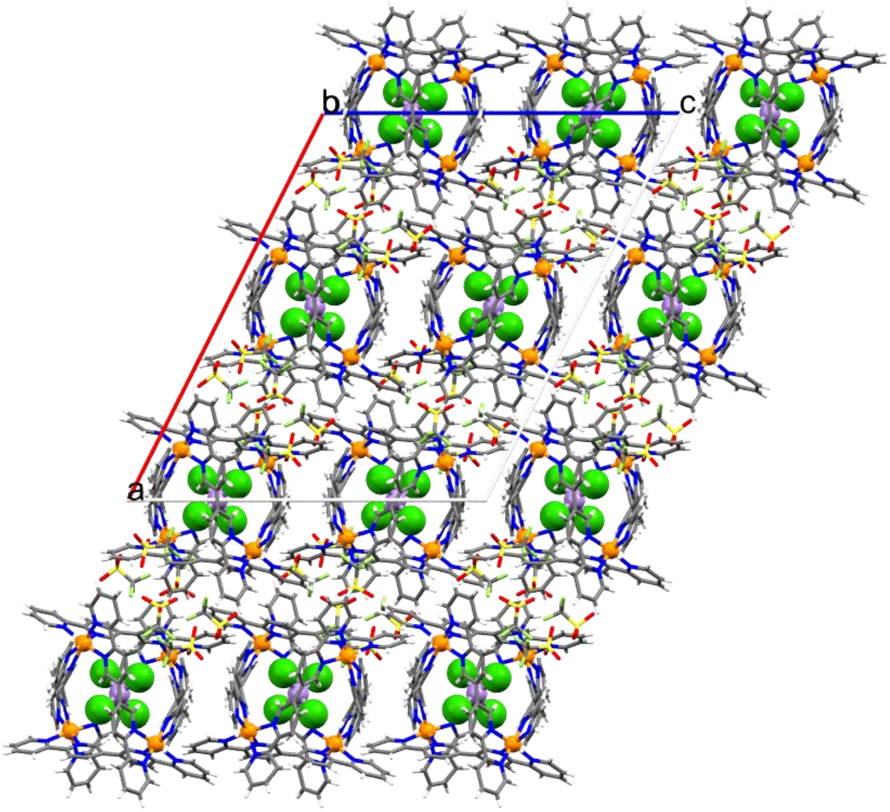

Fig. S22 - Packing of **2** (*C*2/*c*) viewed down the b-axis, illustrating the alternating rows of cages with an encapsulated $[MnCl_4]^{2-}$ followed by a row of triflate anions connecting the cages. Colour code as Fig. S21.

$[Fe^{III}Cl_4 \subset Ni^{II}_4\mathbf{L}_6](OTf)_7$ (**3**)

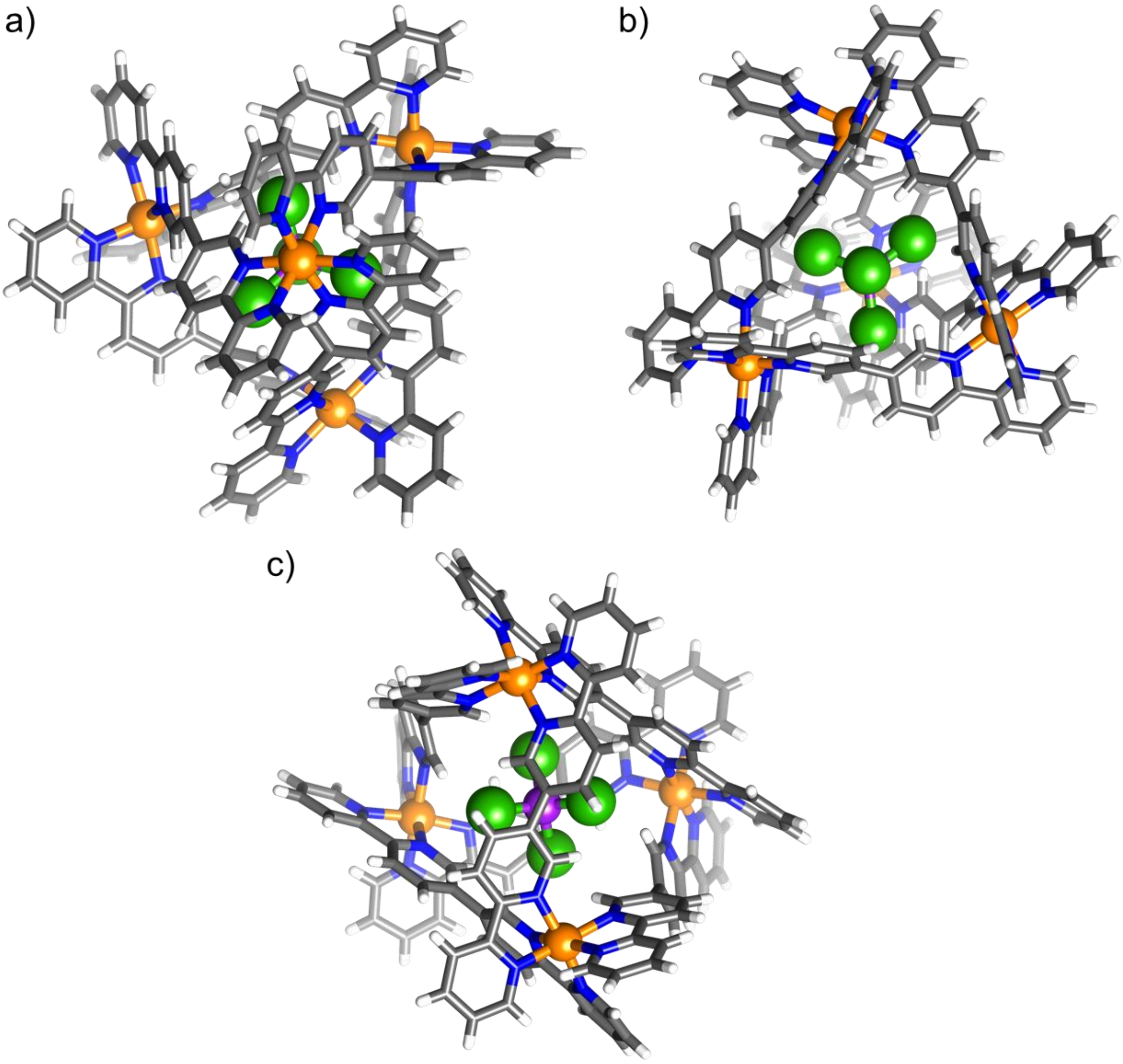

Fig. S23 - (a)-(b) Orthogonal views of complex **3** down a vertex of the cage and through the portal, illustrating the position of the $[FeCl_4]^-$ guest which sits in an inverted tetrahedron with respect to the host cage, with the halide ions pointing out of the cage portals. (c) Side-view of **3** highlighting the connectivity of the ligand in the cage. Colour code: Same as Fig. S18, Cl = green, Fe = purple.

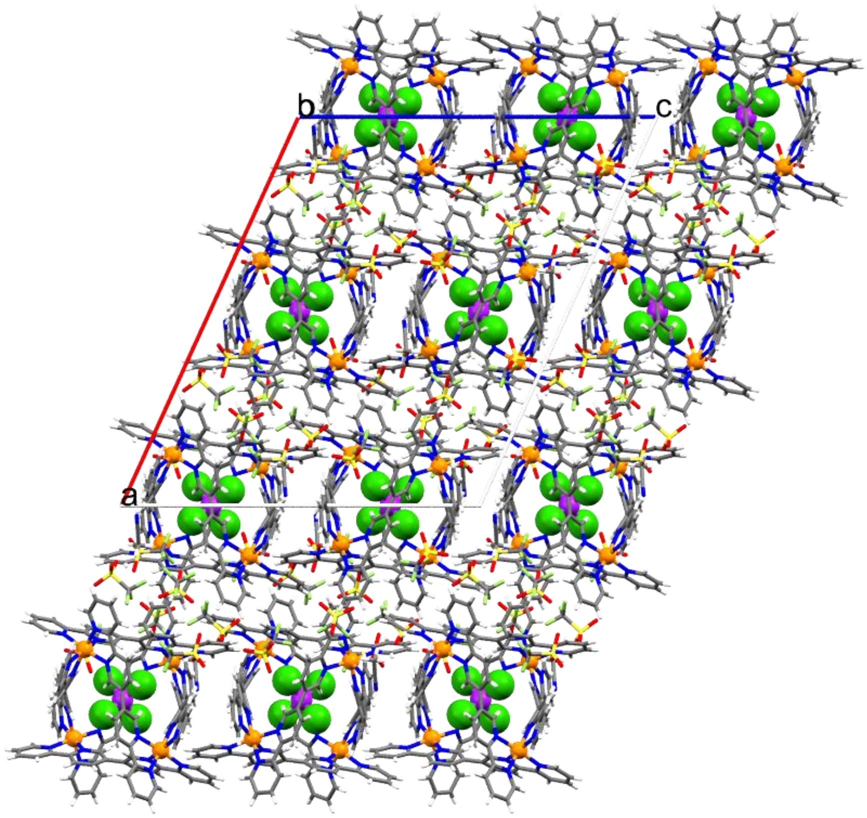

Fig. S24 - Packing of **3** ($C2/c$) viewed down the b-axis, illustrating the alternating rows of cages with an encapsulated $[FeCl_4]^-$ followed by a row of triflate anions connecting the cages. Colour code as Fig. S23.

[Fe^{III}Br$_4$⊂Ni$^{II}_4$**L**$_6$](OTf)$_7$ (**4**)

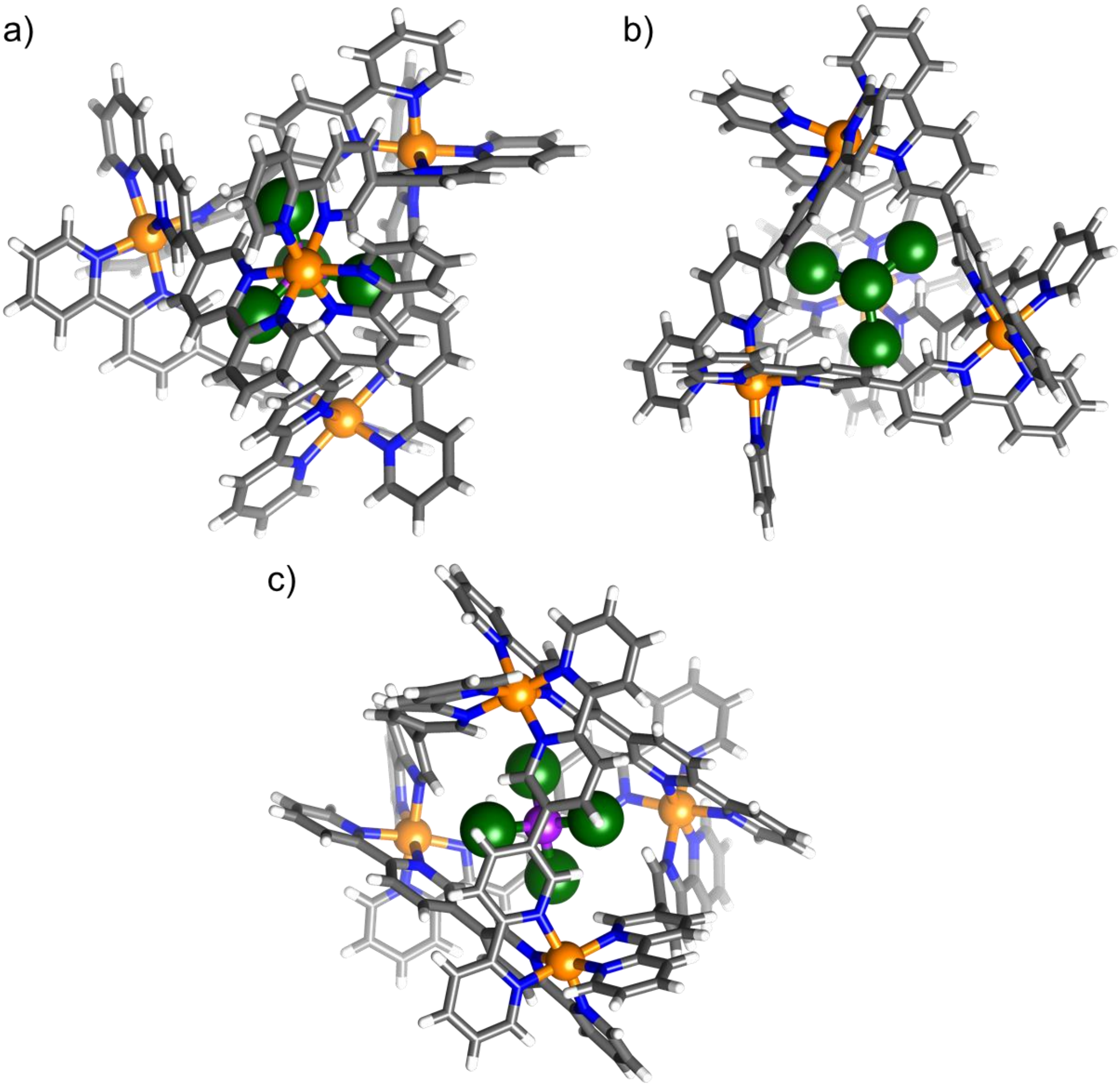

Fig. S25 - (a)-(b) Orthogonal views of complex **4** down a vertex of the cage and through the portal, illustrating the position of the $[FeBr_4]^-$ guest which sits in an inverted tetrahedron with respect to the host cage, with the halide ions pointing out of the cage portals. (c) Side-view of **4** highlighting the connectivity of the ligand in the cage. Colour code: Same as Fig. S23, Br = dark green.

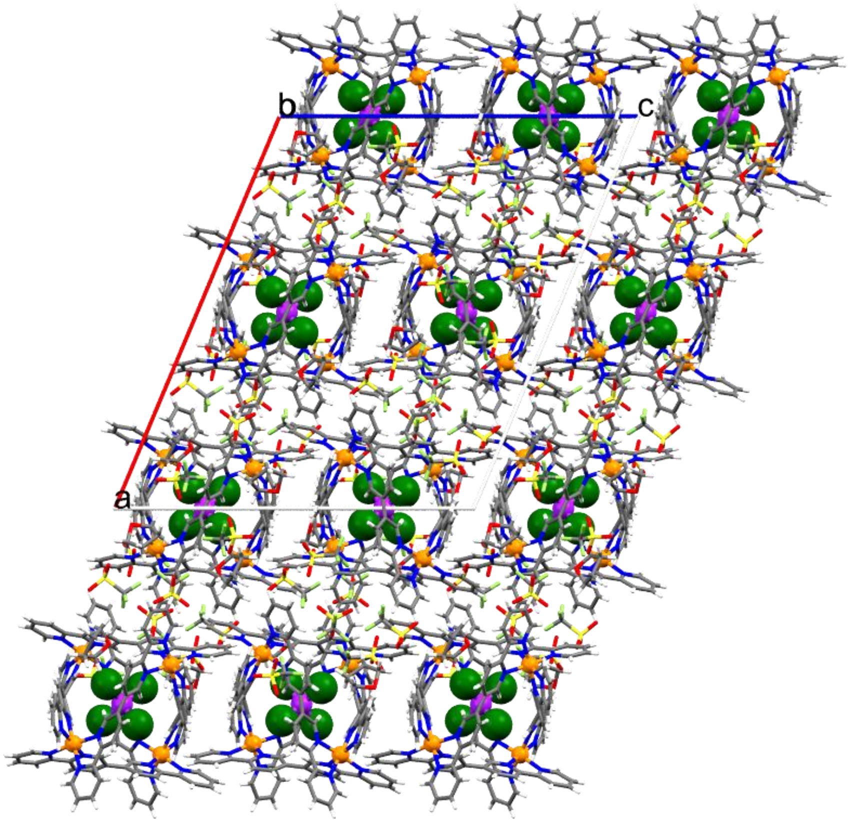

Fig. S26 - Packing of **4** (*C*2/*c*) viewed down the b-axis, illustrating the alternating rows of cages with an encapsulated $[FeBr_4]^-$ followed by a row of triflate anions connecting the cages. Colour code as Fig. S25.

$[Co^{II}Cl_4 \subset Ni^{II}{}_4\mathbf{L}_6](OTf)_6$ (**5**)

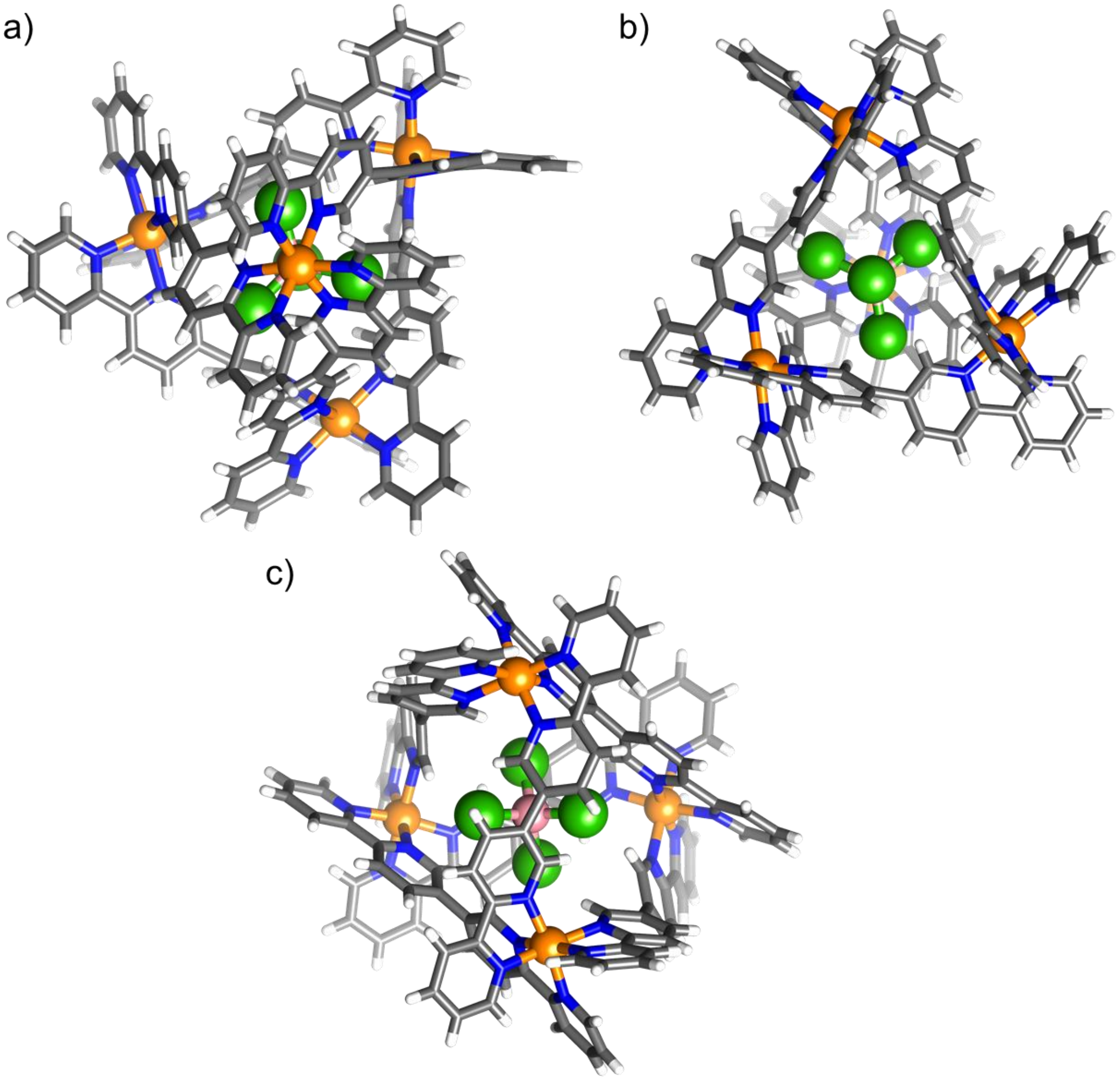

Fig. S27 - (a)-(b) Orthogonal views of complex **5** down a vertex of the cage and through the portal, illustrating the position of the $[CoCl_4]^{2-}$ guest which sits in an inverted tetrahedron with respect to the host cage, with the halide ions pointing out of the cage portals. (c) Side-view of **5** highlighting the connectivity of the ligand in the cage. Colour code: Same as Fig. S18, Cl = green, Co = pink.

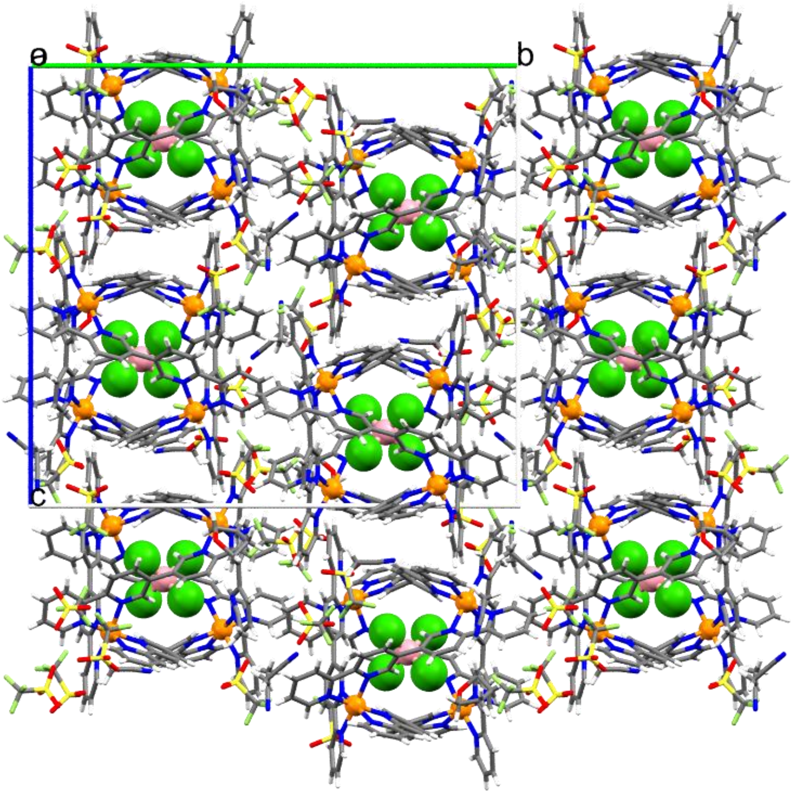

Fig. S28 - Packing of **5** ($P2_1/n$) viewed down the a-axis, illustrating the alternating rows of cages with an encapsulated $[CoCl_4]^{2-}$ followed by a row of triflate anions connecting the cages. Colour code as Fig. S27.

[$Co^{II}Br_4 \subset Ni^{II}{}_4\mathbf{L}_6$]$(OTf)_6$ (**6**)

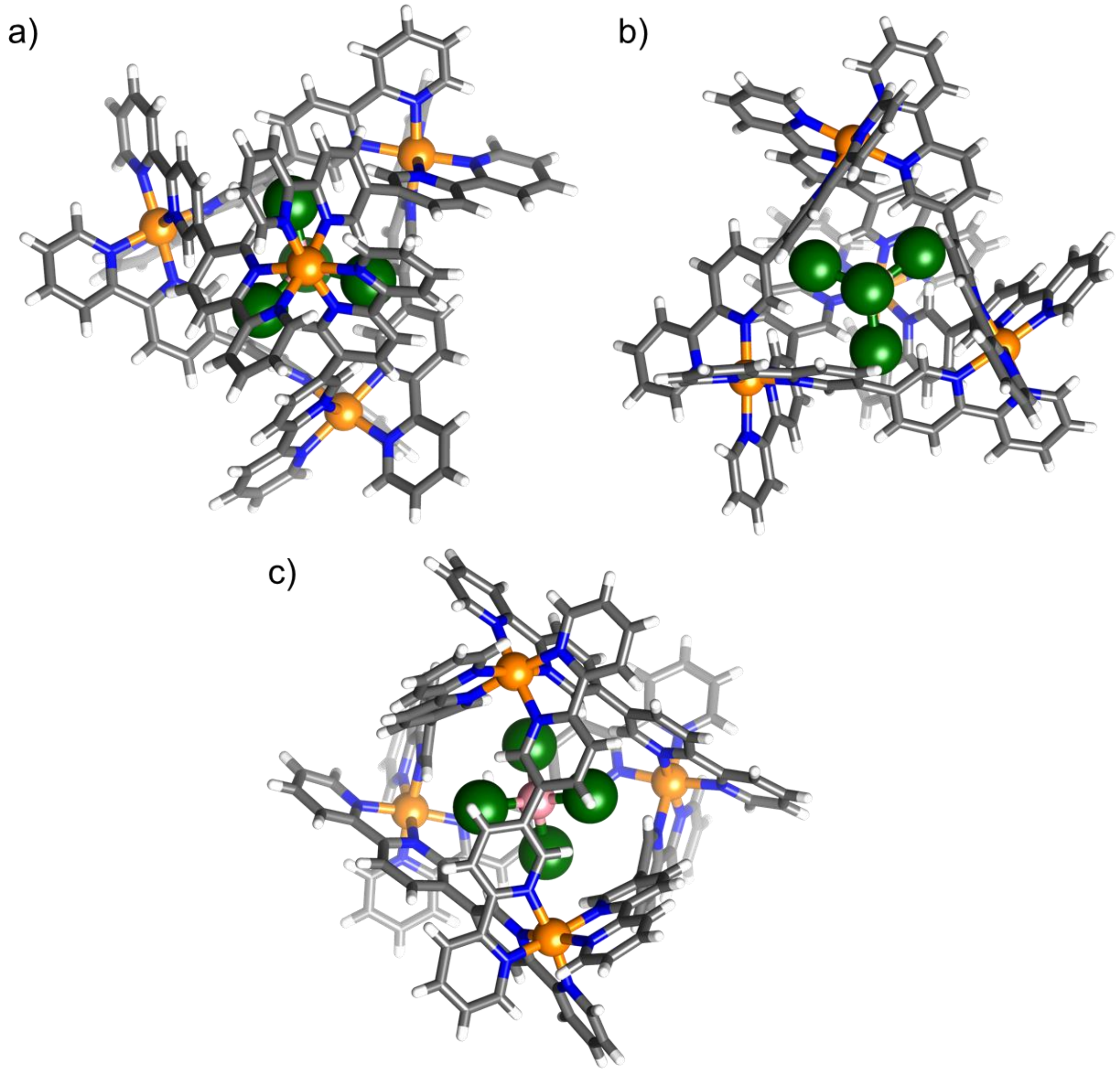

Fig. S29 - (a)-(b) Orthogonal views of complex **6** down a vertex of the cage and through the portal, illustrating the position of the $[CoBr_4]^{2-}$ guest which sits in an inverted tetrahedron with respect to the host cage, with the halide ions pointing out of the cage portals. (c) Side-view of **6** highlighting the connectivity of the ligand in the cage. Colour code: Same as Fig. S27, Br = dark green.

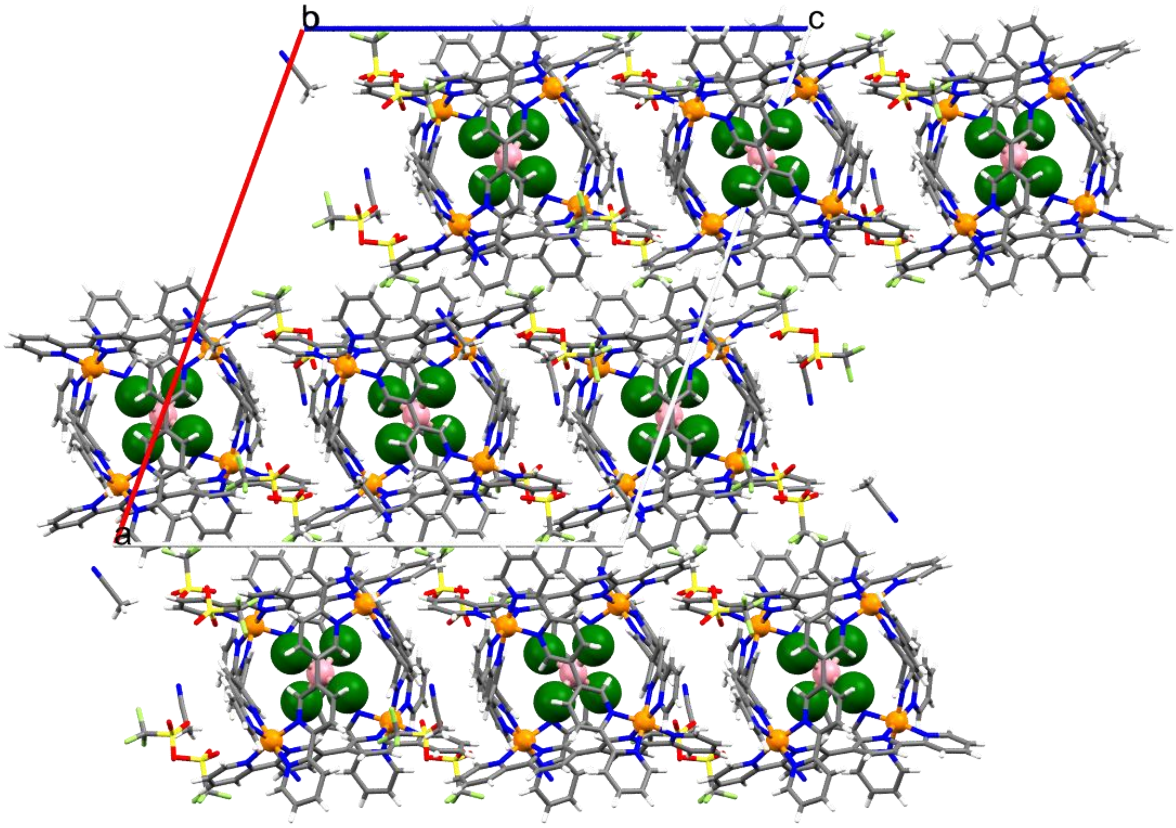

Fig. S30 - Packing of **6** (*P2₁/c*) viewed down the b-axis, illustrating the alternating rows of cages with an encapsulated $[CoBr_4]^{2-}$ followed by a row of triflate anions connecting the cages. Colour code as Fig. S29.

$[Ni^{II}Cl_4 \subset Ni^{II}_4\mathbf{L}_6](OTf)_6$ (**7**)

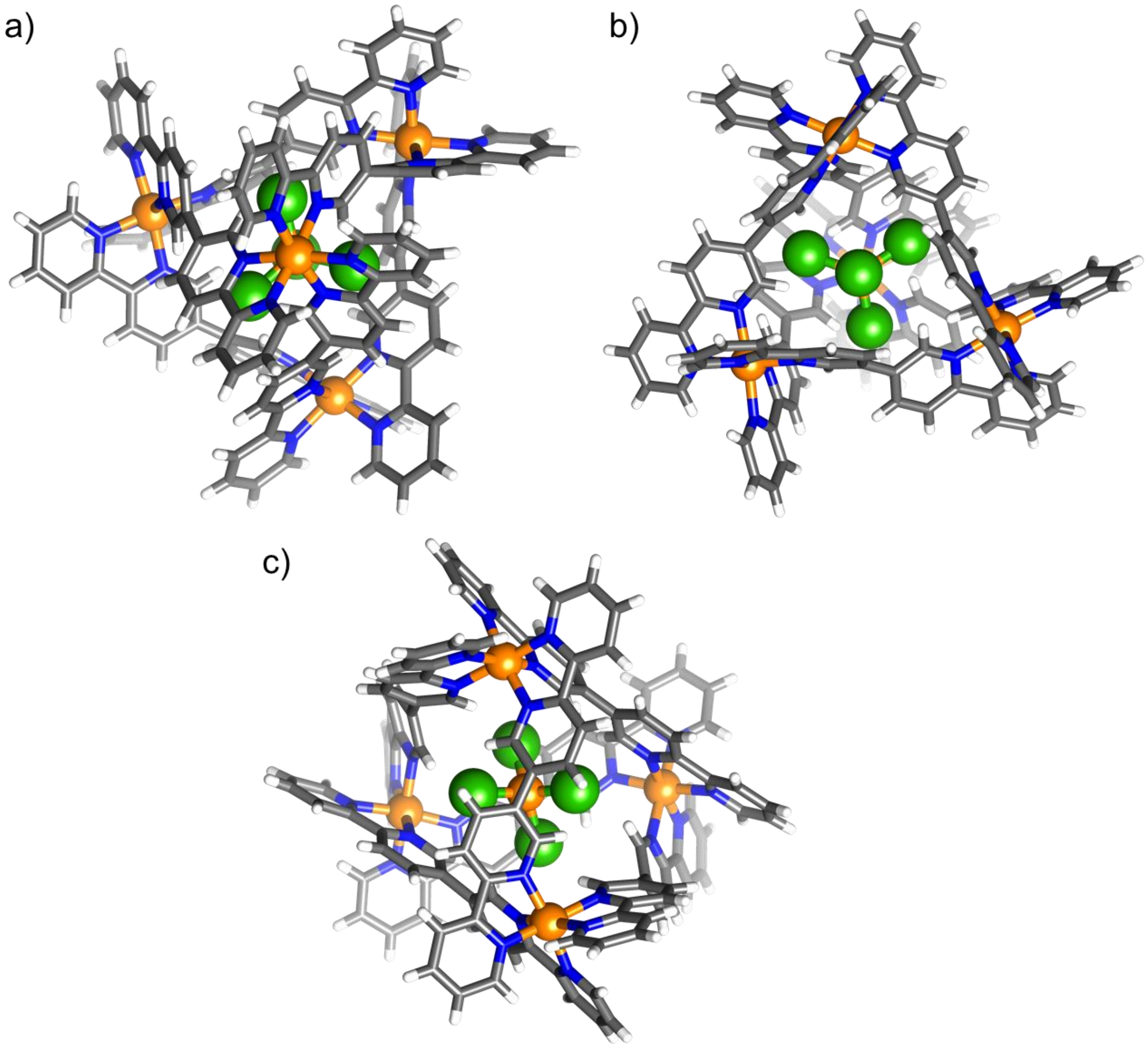

Fig. S31 - (a)-(b) Orthogonal views of complex **7** down a vertex of the cage and through the portal, illustrating the position of the $[NiCl_4]^{2-}$ guest which sits in an inverted tetrahedron with respect to the host cage, with the halide ions pointing out of the cage portals. (Fig. 2a and b in main text). Side-view of **7** highlighting the connectivity of the ligand in the cage. Colour code: Same as Fig. S18, Cl = green.

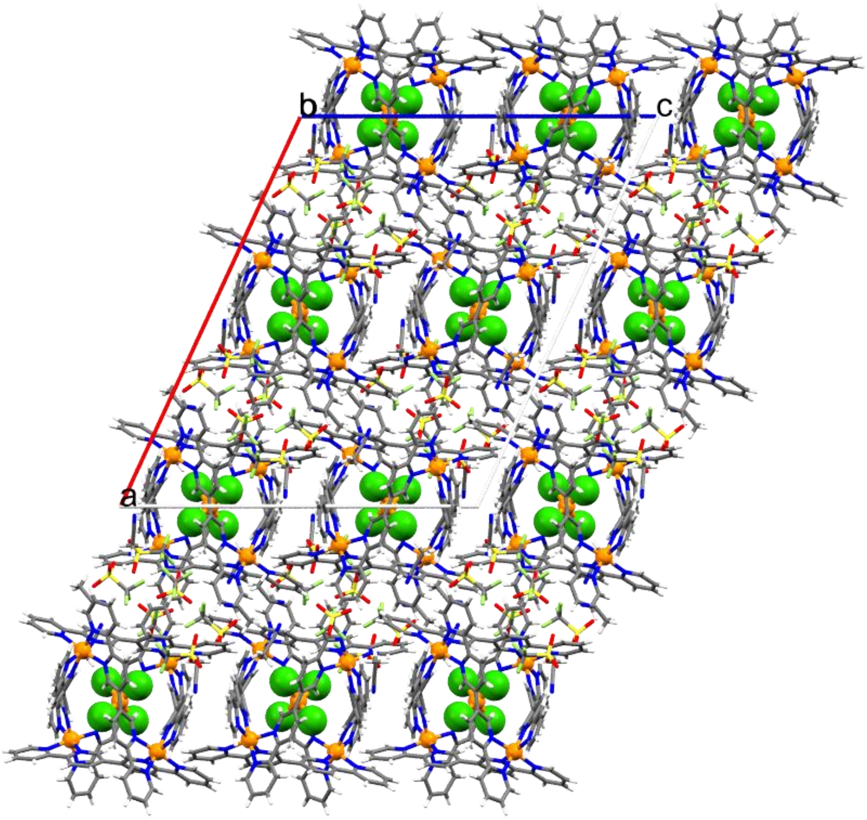

Fig. S32 - Packing of **7** ($C2/c$) viewed down the b-axis, illustrating the alternating rows of cages with an encapsulated $[NiCl_4]^{2-}$ followed by a row of triflate anions connecting the cages. Colour code as Fig. S31.

$[Cu^{II}Br_4 \subset Ni^{II}_4\mathbf{L}_6](OTf)_6$ (**8**)

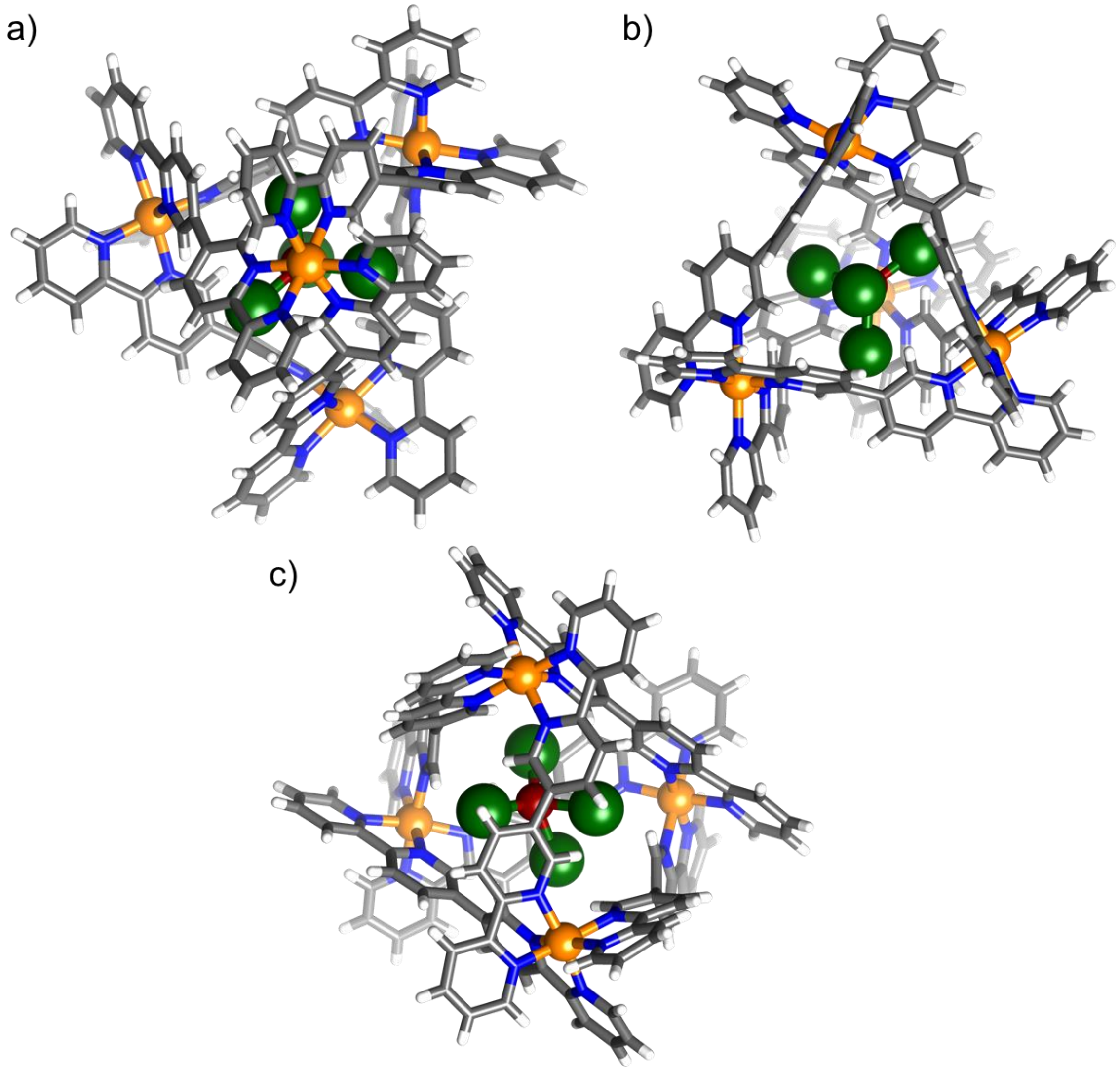

Fig. S33 - (a)-(b) Orthogonal views of complex **8** down a vertex of the cage and through the portal, illustrating the position of the $[CuBr_4]^{2-}$ guest which sits in an inverted tetrahedron with respect to the host cage, with the halide ions pointing out of the cage portals. (c) Side-view of **8** highlighting the connectivity of the ligand in the cage. Colour code: Same as Fig. S18, Br = dark green, Cu = dark red.

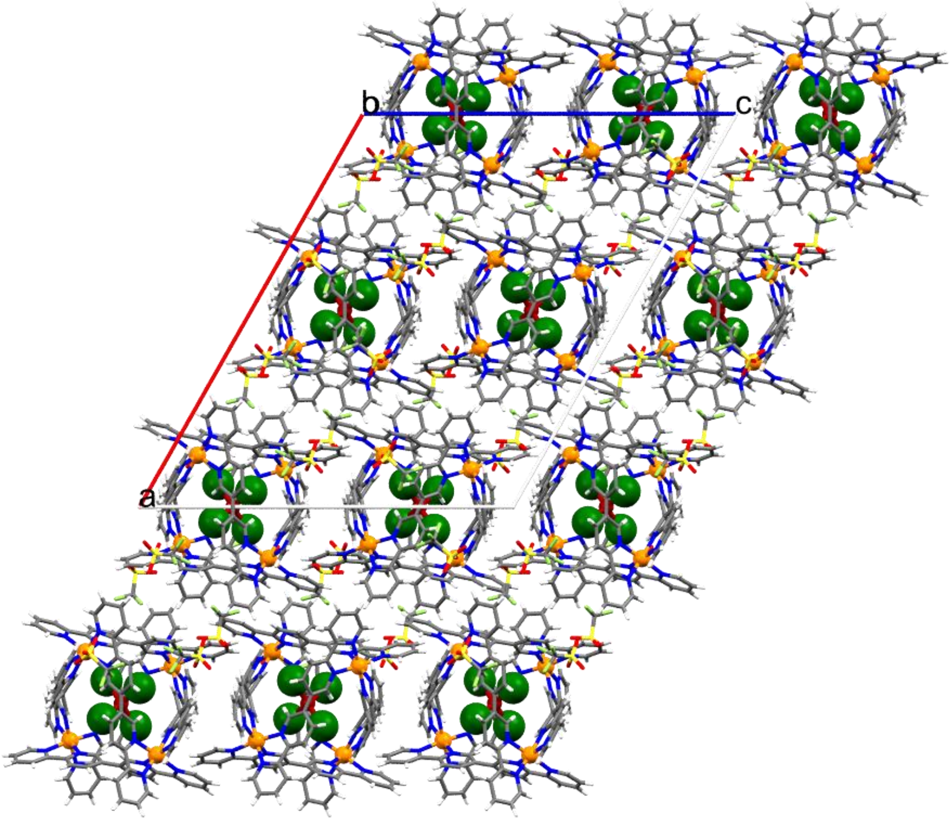

Fig. S34 - Packing of **8** (*C*2/*c*) viewed down the b-axis, illustrating the alternating rows of cages with an encapsulated $[CuBr_4]^{2-}$ followed by a row of triflate anions connecting the cages. Colour code as Fig. S33.

6 Magnetometry

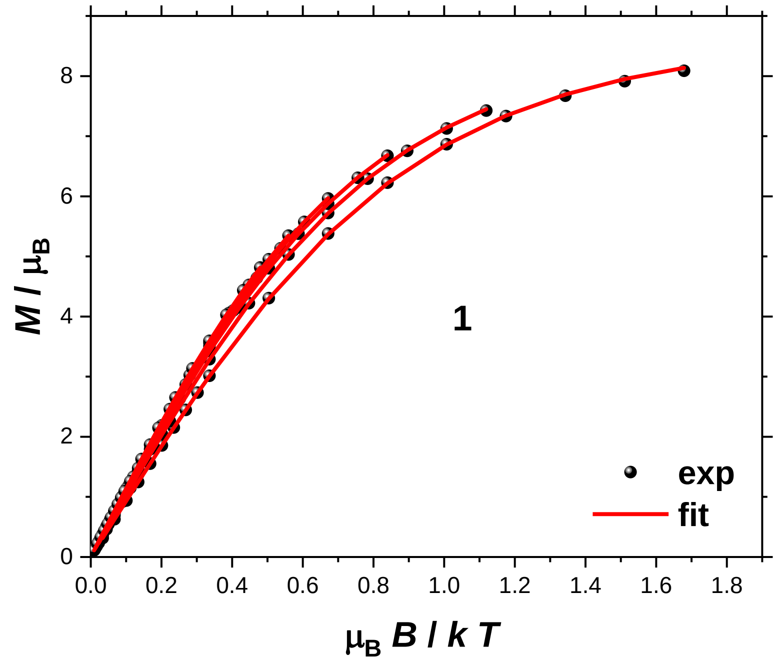

Fig. S35 - Reduced magnetisation plot of **1** in the 2–7 K temperature range and 0–5 T field range, highlighting weak anisotropy by the limited nesting of the curves.

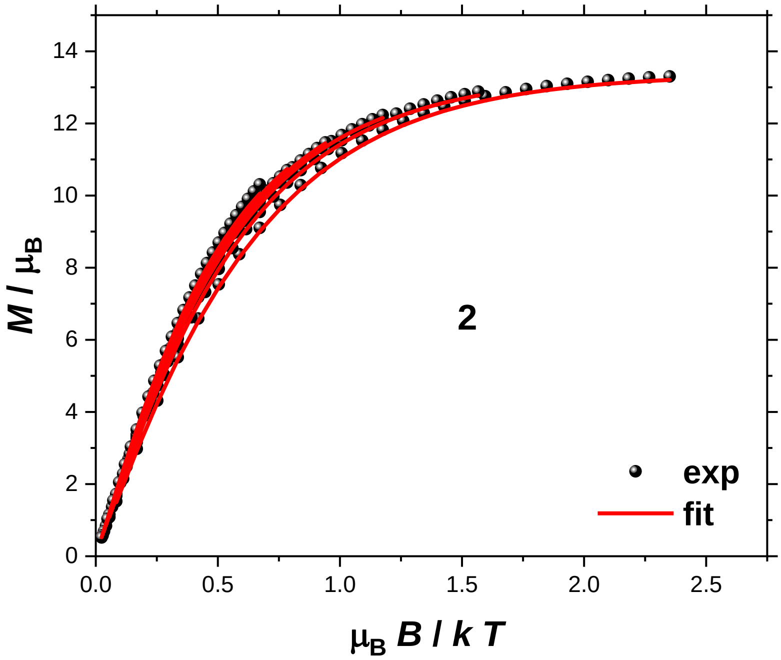

Fig. S36 - Reduced magnetisation plot of **2** in the 2–7 K temperature range and 0–7 T field range, highlighting weak anisotropy by the limited nesting of the curves.

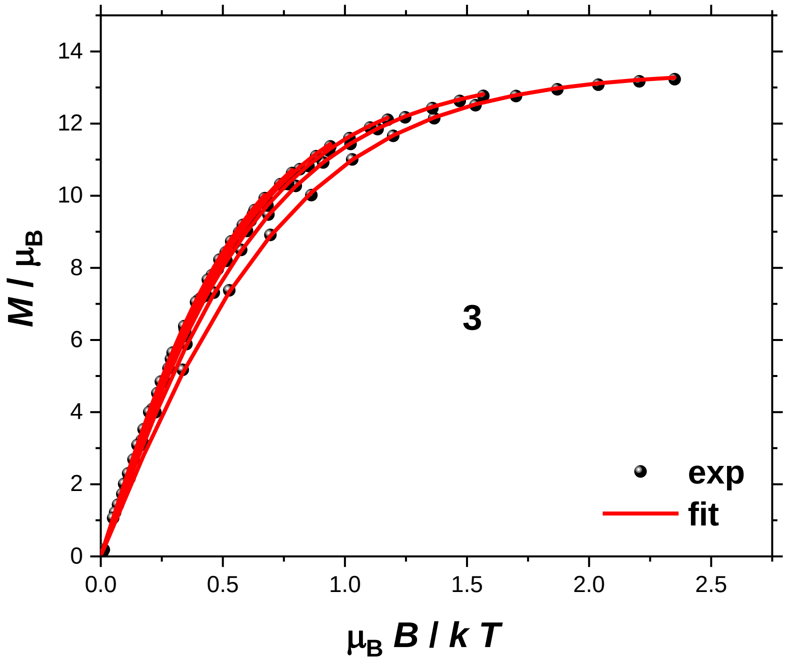

Fig. S37 - Reduced magnetisation plot of **3** in the 2–7 K temperature range and 0–7 T field range, highlighting weak anisotropy by the limited nesting of the curves.

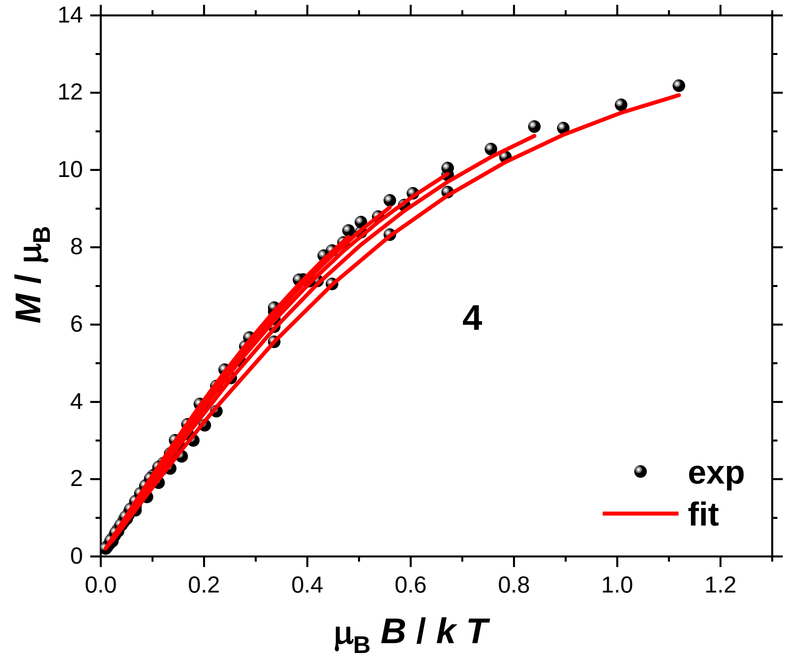

Fig. S38 - Reduced magnetisation plot of **4** in the 3–7 K temperature range and 0–5 T field range, highlighting weak anisotropy by the limited nesting of the curves.

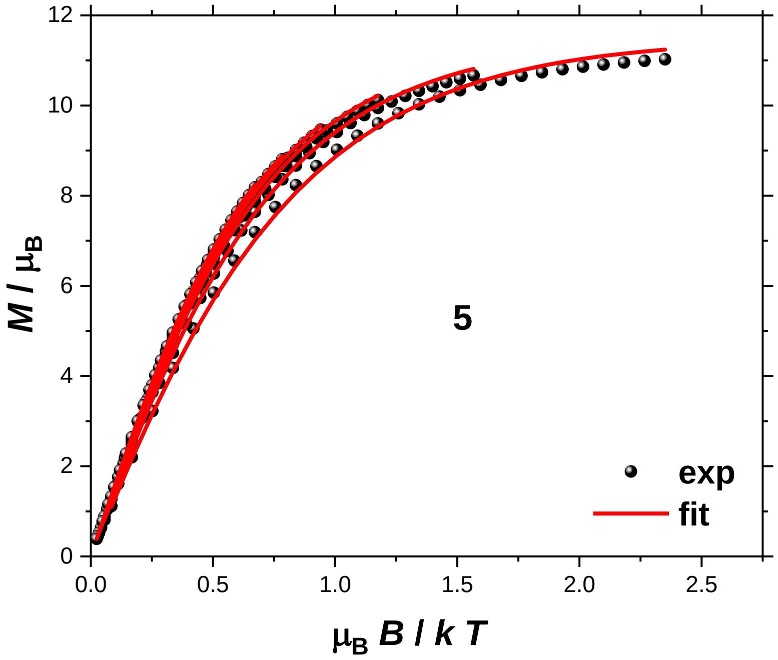

Fig. S39 - Reduced magnetisation plot of **5** in the 2–7 K temperature range and 0–7 T field range, highlighting weak anisotropy by the limited nesting of the curves.

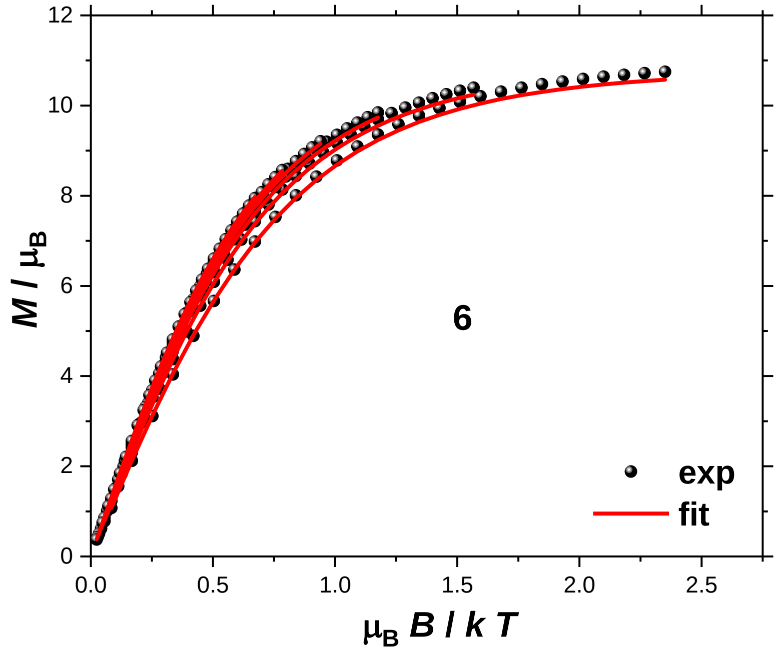

Fig. S40 - Reduced magnetisation plot of **6** in the 2–7 K temperature range and 0–7 T field range, highlighting weak anisotropy by the limited nesting of the curves.

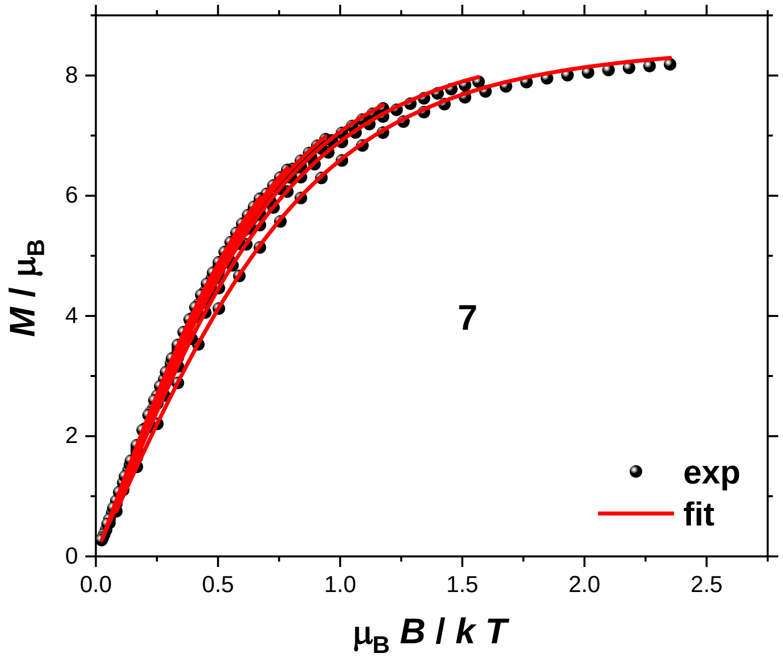

Fig. S41 - Reduced magnetisation plot of **7** in the 2–7 K temperature range and 0–7 T field range, highlighting weak anisotropy by the limited nesting of the curves.

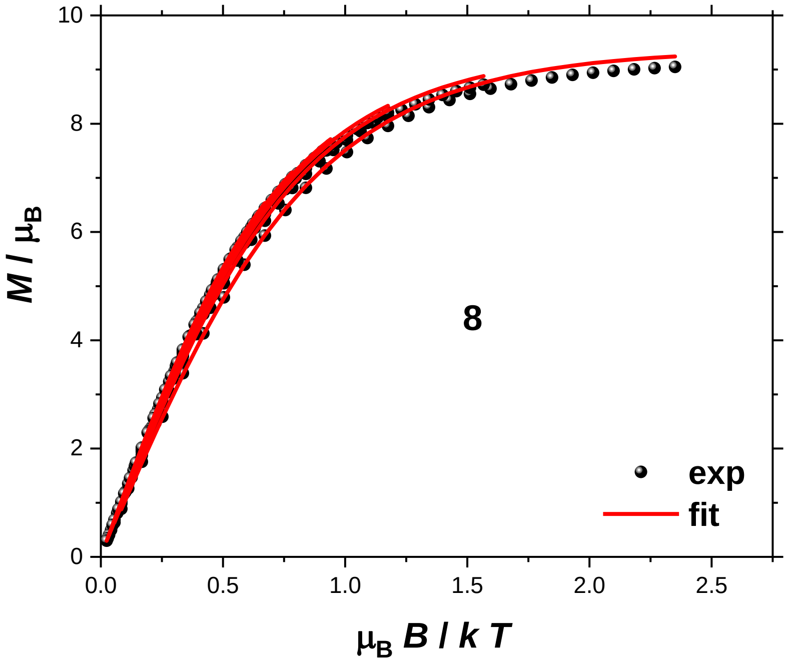

Fig. S42 - Reduced magnetisation plot of **8** in the 2–7 K temperature range and 0–7 T field range, highlighting weak anisotropy by the limited nesting of the curves.

7 Computational Details

Ab initio method: All single point multi-configuration SCF calculations were carried on the X-ray geometries using the ORCA 4.0.1 program code.[15] Spin-Hamiltonian (SH) parameters were computed from CASSCF/NEVPT2 methodology. NEVPT2 (N-electron valence state perturbation theory) calculations were performed in combination with the CASSCF (complete active space self-consistent field) wavefunction to recover the dynamic electron correlation. While calculating the on-site SH parameters on certain paramagnetic ions the remaining paramagnetic centres were masked with diamagnetic ions (*e.g*. Zn^{II}). While calculating the SH parameters on the guest molecule, the host cage atoms were replaced with CHELPG charges obtained from UKS/BP86 DFT calculations. Douglas-Kroll-Hess Hamiltonian (second-order) was considered for the scalar relativistic corrections. DKH- version of contracted def2- basis sets- DKH-def2-TZVP for the Cu, Ni, Co, Fe, Mn, Cl and Br atoms; DKH-def2-TZVP(-f) for N and DKH-def2-SVP for the rest of the atoms were used during the calculations. For the starting orbitals a UKS/BP86 DFT calculation was performed and the resulting quasi-restricted orbitals (QROs) were used in the following configuration interaction step. Active space was chosen as CAS(n,5), where n is the number of electrons in the valence d-orbitals of the metals. The active orbitals were optimised with 5 doublets for Cu(II), 10 triplets and 15 singlets for Ni(II), 10 quartets and 40 doublets for Co(II); 1 sextet and 24 quartets for Fe(III) and Mn(II) species. Spin-orbit coupling effects were included from the quasi-degenerate perturbation theory (QDPT) approach with spin-orbit mean field (SOMF) operator. Final spin-Hamiltonian parameters such as *g*-factors, *D* and *E* parameters were determined from effective Hamiltonian approach (EHA).[16]

DFT method: To obtain magnetic exchange interactions between the paramagnetic metal centres, single point Density Functional Theory (DFT) calculations were performed on the X-ray structures using the Gaussian 09 program.[17] Broken symmetry methodology was employed using the fragmentation method to obtain the magnetic coupling constants.[18] The unrestricted B3LYP functional was used with Ahlrich's all electron triple zeta valence (TZV) basis set for all atoms.[19,20] Wavefunction reoptimisation was performed after the SCF convergence to check the stability of the wavefunction. Geometry optimisation was also carried out for the anionic $[NiCl_4]^{2-}$ guest with the B3LYP/TZVP level of theory and basis set to compare the change in

geometry and zero-field splitting before and after the insertion to the cage. The isotropic coupling constant J was computed from the following pairwise interaction formula.[21]

$$J = \frac{E_{BS} - E_{HS}}{2(2S_1S_2 + S_2)}$$

Table S6 - Spin density values obtained from the uB3LYP/TZV level of theory on the metal ions in complexes **1–8** (excluding complex **7**).

Complexes 1-8 (Excluding complex 7)	HS Spin density (ρ^{α}-ρ^{β})	BS1 Spin density (ρ^{α}-ρ^{β})	BS2 Spin density (ρ^{α}-ρ^{β})	BS3 Spin density (ρ^{α}-ρ^{β})
Ni1, Ni2, Ni3, Ni4	1.64,1.65,1.64,1.65	-1.64, 1.65, 1.64, 1.65	-1.64, -1.65, 1.64, 1.65	-1.64, 1.65, -1.64, 1.65
Ni1, Ni2, Ni3, Ni4, Mn	1.63,1.65,1.63,1.65, 4.70	1.63,1.65,1.63,1.65, -4.70	-1.63,1.65,1.63,1.65, 4.70	-1.63, -1.65,1.63,1.65, 4.70
Ni1, Ni2, Ni3, Ni4, Fe	1.64,1.65,1.64,1.65,3.85	1.65,1.65,1.65,1.65, -3.85	-1.65,1.65,1.64,1.65,3.85	-1.65, 1.65, -1.65,1.65,3.85
Ni1, Ni2, Ni3, Ni4, Fe	1.64,1.65,1.64,1.65,3.76	1.65,1.65,1.65,1.65, -3.76	-1.65,1.65,1.64,1.65,3.76	-1.65,1.65, -1.65,1.65,3.76
Ni1, Ni2, Ni3, Ni4, Co	1.65,1.65,1.65,1.65,2.59	1.65,1.65,1.65,1.65, -2.59	-1.65,1.65,1.65,1.65, 2.59	-1.65,1.65, -1.65,1.65, 2.59
Ni1, Ni2, Ni3, Ni4, Co	1.65,1.64,1.64,1.65,2.56	1.65,1.64,1.65,1.65, -2.56	-1.65,1.64,1.64,1.65,2.56	-1.65, -1.64,1.64,1.65,2.56
Ni1, Ni2, Ni3, Ni4, Cu	1.65,1.66,1.66,1.65,0.39	1.65,1.66,1.66,1.65, -0.39	-1.65,1.66,1.66,1.65, 0.39	-1.65, -1.66,1.66,1.65, 0.39

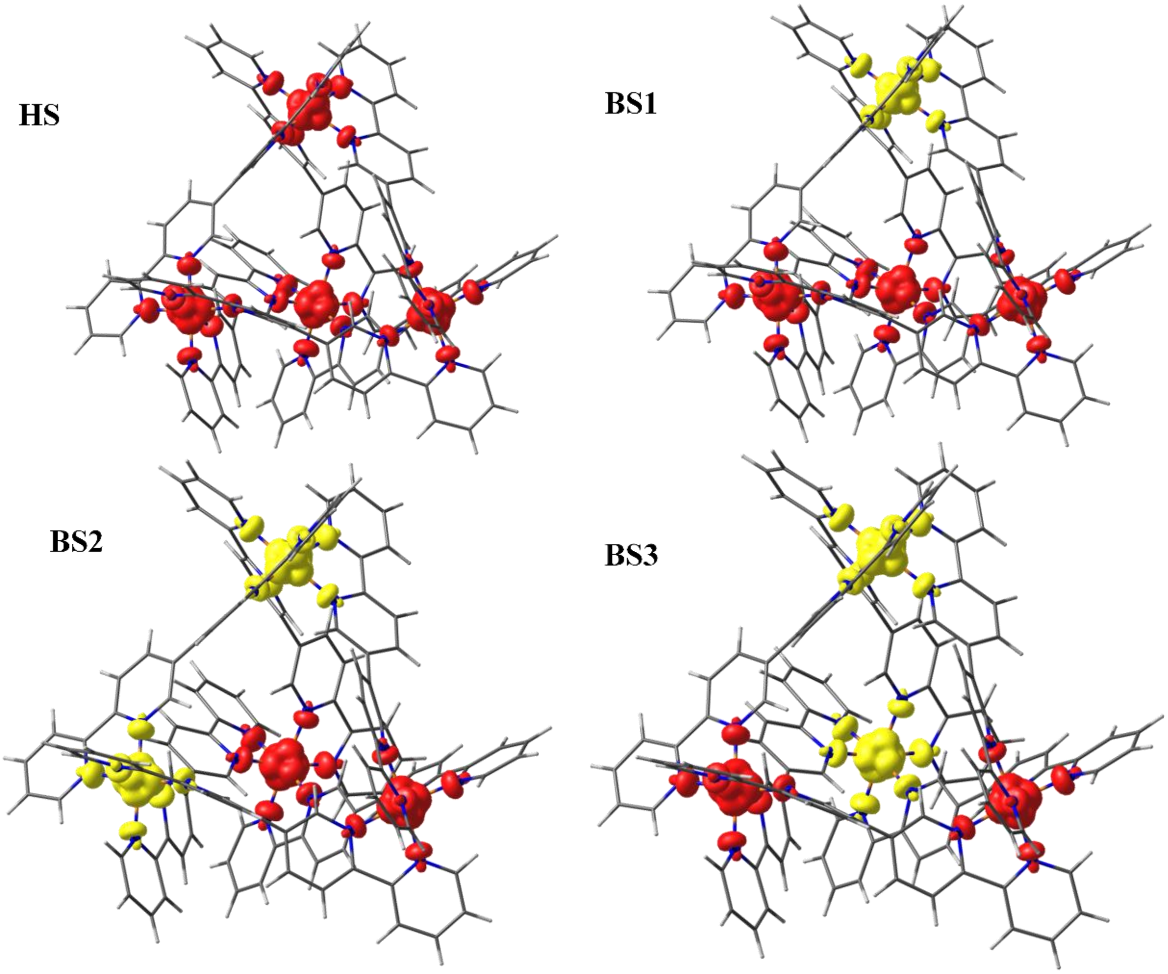

Fig. S43 - High spin and four broken symmetry spin density plots for complex **1** obtained from the uB3LYP/TZV level of theory. The iso-surface cut-off was chosen to be 0.006 $e^-/Bohr^3$.

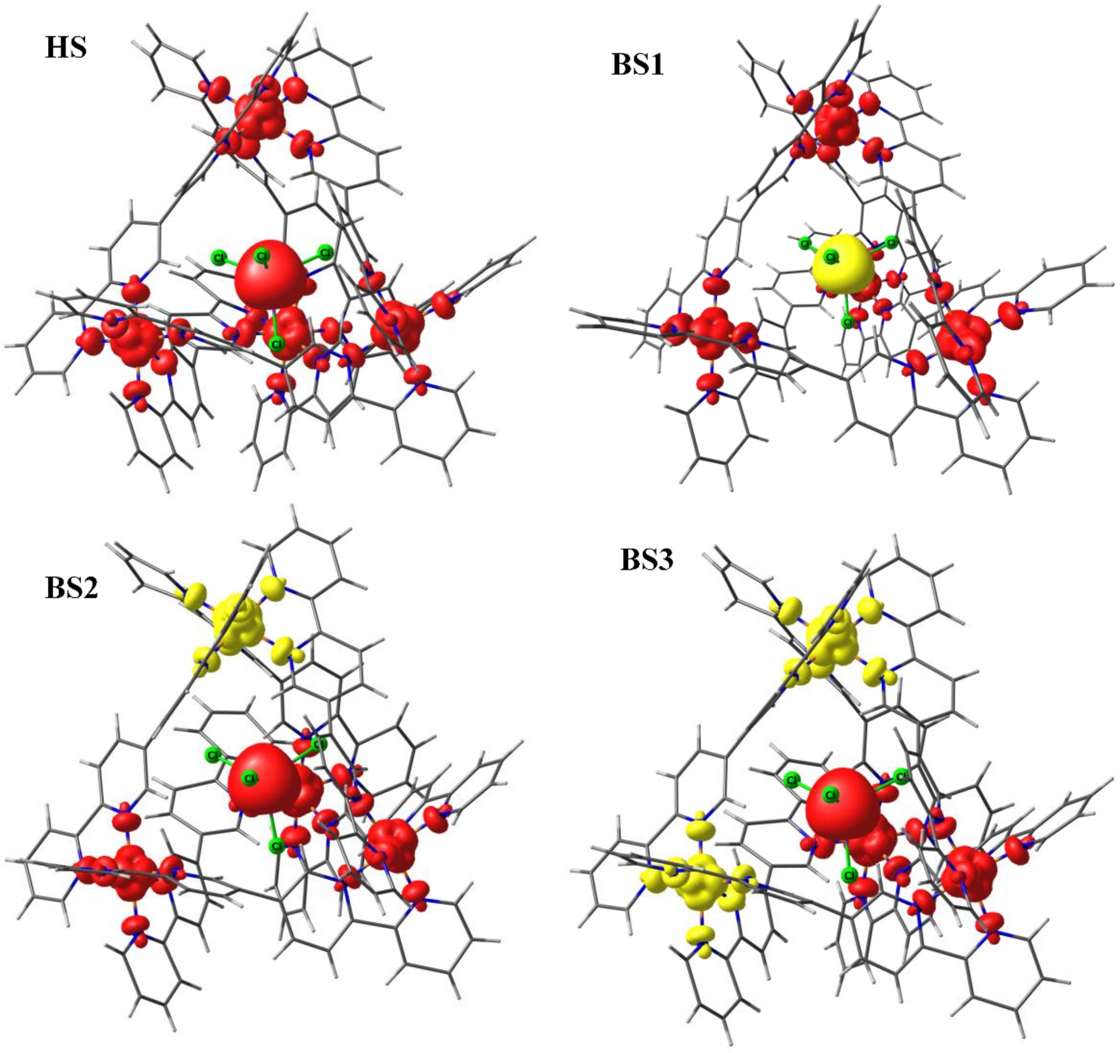

Fig. S44 High spin and four broken symmetry spin density plots for complex **2** obtained from the uB3LYP/TZV level of theory. The iso-surface cut-off was chosen to be 0.006 $e^-/Bohr^3$.

Table S7 - Multi-determinant electronic configurations and their respective contributions towards the *D* and *E* parameters for the $[NiCl_4]^{2-}$ guest in complex **7**.

$[NiCl_4]^{2-}$ ligand field states	NEVPT2 transition energies (cm^{-1})	Electronic configuration	Contribution to *D* (cm^{-1})	Contribution to *E* (cm^{-1})
1	0.0	$(d_{x^2-y^2})^2(d_{z^2})^2(d_{xy})^2(d_{yz})^1(d_{xz})^1$ (86%)	0.0	0.0
2	619	$(d_{x^2-y^2})^2(d_{z^2})^2(d_{xy})^1(d_{yz})^2(d_{xz})^1$ (47%)<br>$(d_{x^2-y^2})^1(d_{z^2})^2(d_{xy})^2(d_{yz})^2(d_{xz})^1$ (31%)	143.8	143.7
3	1495	$(d_{x^2-y^2})^2(d_{z^2})^2(d_{xy})^1(d_{yz})^1(d_{xz})^2$ (46%)<br>$(d_{x^2-y^2})^1(d_{z^2})^2(d_{xy})^2(d_{yz})^1(d_{xz})^2$ (29%)	73.7	-73.6
4	2703	$(d_{x^2-y^2})^1(d_{z^2})^2(d_{xy})^2(d_{yz})^2(d_{xz})^1$ (44%)<br>$(d_{x^2-y^2})^2(d_{z^2})^2(d_{xy})^1(d_{yz})^2(d_{xz})^1$ (26%)	61.8	-61.6
5	3778	$(d_{x^2-y^2})^1(d_{z^2})^2(d_{xy})^2(d_{yz})^1(d_{xz})^2$ (45%)<br>$(d_{x^2-y^2})^2(d_{z^2})^2(d_{xy})^1(d_{yz})^1(d_{xz})^2$ (24%)	46.3	46.0

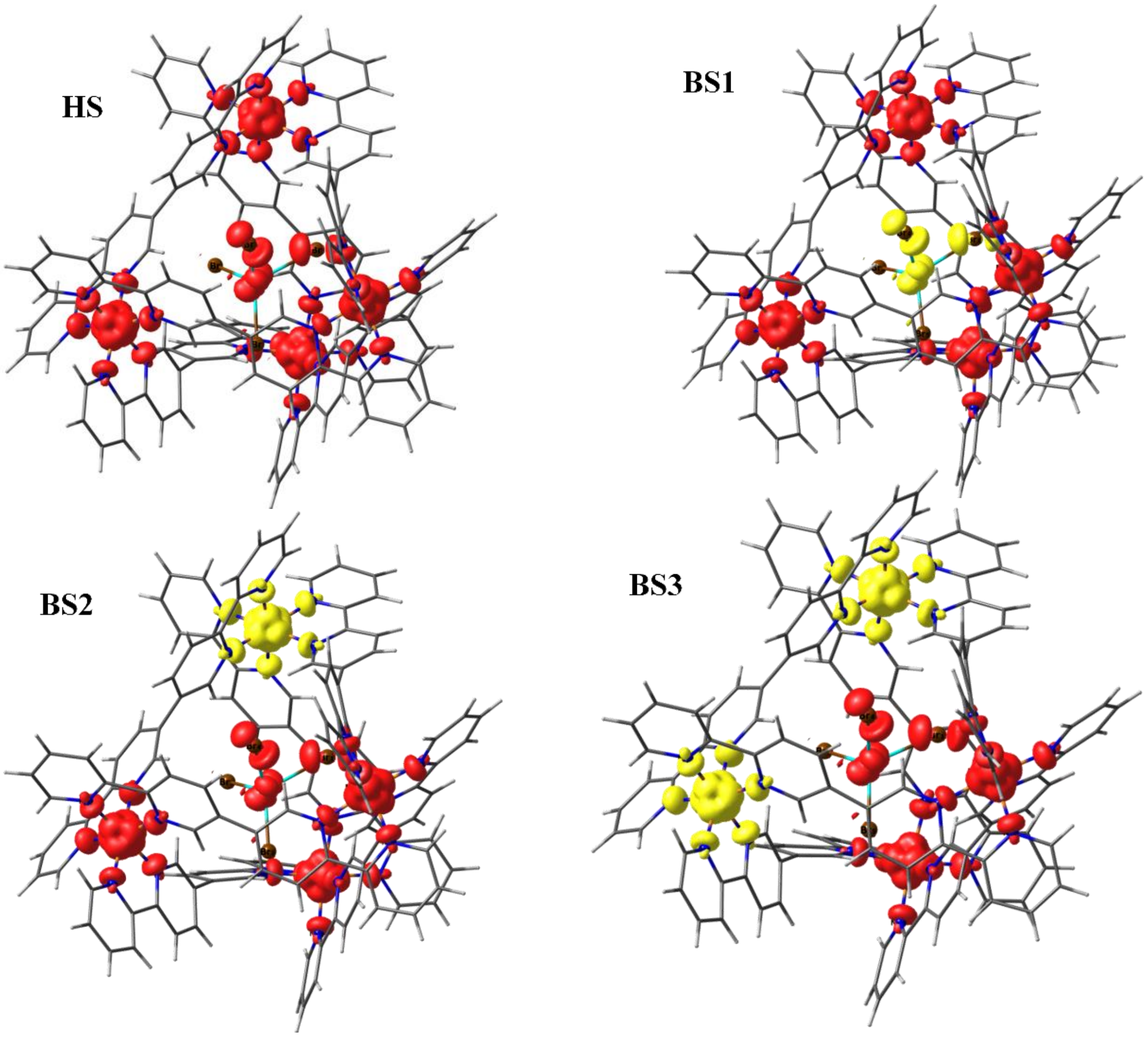

Fig. S45 - High spin and four broken symmetry spin density plots for complex **8** obtained from the uB3LYP/TZV level of theory. The iso-surface cut-off was chosen to be 0.006 $e^{-}/Bohr^{3}$.

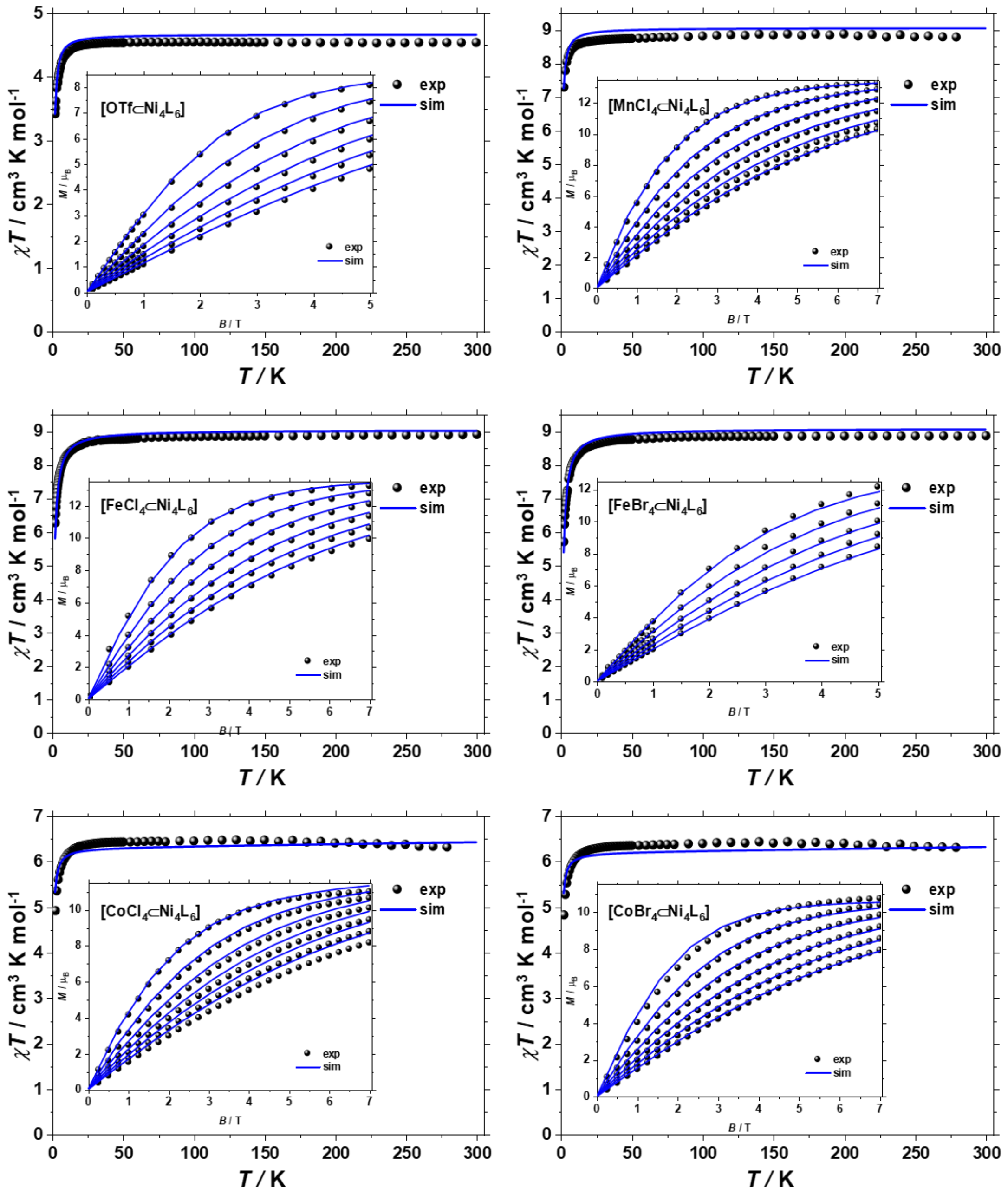

Fig. S46 - Comparison of experimental (black symbols) and theoretically computed (blue lines) χT vs T and M vs B data for complexes **1**-**6**.

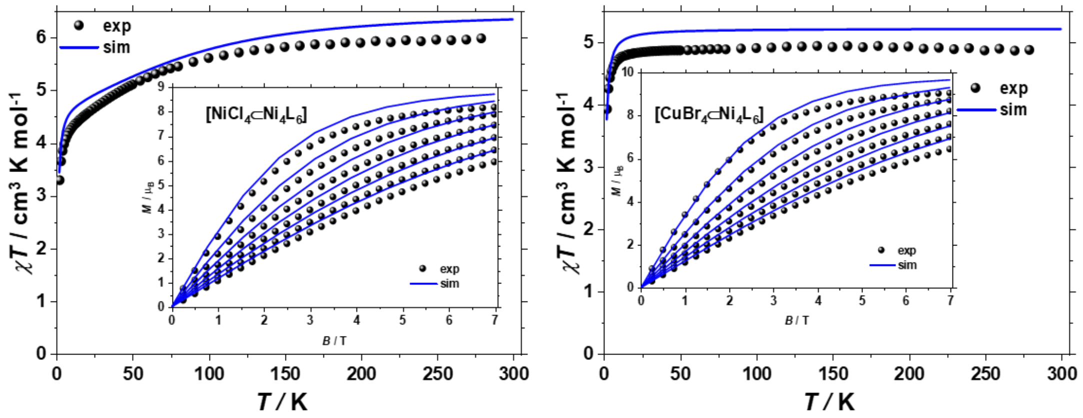

Fig. S47 - Comparison of experimental (black symbols) and theoretically computed (blue lines) χT vs T and M vs B data for complexes **7** and **8**

8 References

1 S. Y. Tyree, *Inorganic Syntheses*, John Wiley & Sons, Inc., Hoboken, NJ, USA, 1967, vol. 9.

2 J. Chen, M. Kuss-Petermann and O. S. Wenger, *Chem. - A Eur. J.*, 2014, **20**, 4098–4104.

3 B. J. Cosier and A. M. Glazer, *J. Appl. Crystallogr.*, 1986, **19**, 105–107.

4 G. M. Sheldrick, *Acta Crystallogr. Sect. A Found. Crystallogr.*, 2015, **71**, 3–8.

5 O. V. Dolomanov, L. J. Bourhis, R. J. Gildea, J. A. K. Howard and H. Puschmann, *J. Appl. Crystallogr.*, 2009, **42**, 339–341.

6 G. M. Sheldrick, *Acta Crystallogr. Sect. C Struct. Chem.*, 2015, **71**, 3–8.

7 A. L. Spek, *Acta Crystallogr. Sect. C Struct. Chem.*, 2015, **71**, 9–18.

8 A. L. Spek, *J. Appl. Crystallogr.*, 2003, **36**, 7–13.

9 N. T. Johnson, P. G. Waddell, W. Clegg and M. R. Probert, *Crystals*, 2017, **7**, 360.

10 H. Nowell, S. A. Barnett, K. E. Christensen, S. J. Teat and D. R. Allan, *J. Synchrotron Radiat.*, 2012, **19**, 435–441.

11 G. Winter, D. G. Waterman, J. M. Parkhurst, A. S. Brewster, R. J. Gildea, M. Gerstel, L. Fuentes-Montero, M. Vollmar, T. Michels-Clark, I. D. Young, N. K. Sauter and G. Evans, *Acta Crystallogr. Sect. D Struct. Biol.*, 2018, **74**, 85–97.

12 P. Evans, in *Acta Crystallographica Section D: Biological Crystallography*, International Union of Crystallography, 2006, vol. 62, pp. 72–82.

13 R. W. Grosse-Kunstleve, N. K. Sauter, N. W. Moriarty and P. D. Adams, *J. Appl. Crystallogr.*, 2002, **35**, 126–136.

14 M. Miklitz and K. E. Jelfs, *J. Chem. Inf. Model.*, 2018, **58**, 2387–2391.

15 F. Neese, *Wiley Interdiscip. Rev. Comput. Mol. Sci.*, 2012, **2**, 73–78.

16 R. Maurice, R. Bastardis, C. de Graaf, N. Suaud, T. Mallah and N. Guihéry, *J. Chem. Theory Comput.*, 2009, **5**, 2977–2984.

17 M. J. Frisch, G. W. Trucks, H. B. Schlegel, G. E. Scuseria, M. A. Robb, J. R.

Cheeseman, G. Scalmani, V. Barone, G. A. Petersson, H. Nakatsuji, X. Li, M. Caricato, A. V Marenich, J. Bloino, B. G. Janesko, R. Gomperts, B. Mennucci, H. P. Hratchian, J. V Ortiz, A. F. Izmaylov, J. L. Sonnenberg, D. Williams-Young, F. Ding, F. Lipparini, F. Egidi, J. Goings, B. Peng, A. Petrone, T. Henderson, D. Ranasinghe, V. G. Zakrzewski, J. Gao, N. Rega, G. Zheng, W. Liang, M. Hada, M. Ehara, K. Toyota, R. Fukuda, J. Hasegawa, M. Ishida, T. Nakajima, Y. Honda, O. Kitao, H. Nakai, T. Vreven, K. Throssell, J. A. Montgomery Jr., J. E. Peralta, F. Ogliaro, M. J. Bearpark, J. J. Heyd, E. N. Brothers, K. N. Kudin, V. N. Staroverov, T. A. Keith, R. Kobayashi, J. Normand, K. Raghavachari, A. P. Rendell, J. C. Burant, S. S. Iyengar, J. Tomasi, M. Cossi, J. M. Millam, M. Klene, C. Adamo, R. Cammi, J. W. Ochterski, R. L. Martin, K. Morokuma, O. Farkas, J. B. Foresman and D. J. Fox, *Gaussian 09, Revision D.01, Gaussian, Inc., Wallingford CT*, 2013.

18 L. Noodleman, *J. Chem. Phys.*, 1981, **74**, 5737–5743.

19 A. Schäfer, C. Huber and R. Ahlrichs, *J. Chem. Phys.*, 1994, **100**, 5829–5835.

20 A. Schäfer, H. Horn and R. Ahlrichs, *J. Chem. Phys.*, 1992, **97**, 2571–2577.

21 E. Ruiz, J. Cano, S. Alvarez and P. Alemany, *J. Comput. Chem.*, 1999, **20**, 1391–1400.
